# Velvet domain protein VosA represses the zinc cluster transcription factor SclB regulatory network for *Aspergillus nidulans* asexual development, oxidative stress response and secondary metabolism

**DOI:** 10.1371/journal.pgen.1007511

**Published:** 2018-07-25

**Authors:** Karl G. Thieme, Jennifer Gerke, Christoph Sasse, Oliver Valerius, Sabine Thieme, Razieh Karimi, Antje K. Heinrich, Florian Finkernagel, Kristina Smith, Helge B. Bode, Michael Freitag, Arthur F. J. Ram, Gerhard H. Braus

**Affiliations:** 1 Department of Molecular Microbiology and Genetics and Goettingen Center for Molecular Biosciences (GZMB), University of Goettingen, Goettingen, Germany; 2 Molekulare Biotechnologie, Fachbereich Biowissenschaften, Goethe Universität Frankfurt, Frankfurt am Main, Germany; 3 Institute of Molecular Biology and Tumor Research (IMT), Philipps-University, Marburg, Germany; 4 Department of Biochemistry and Biophysics, Centre for Genome Research and Biocomputing, Oregon State University, Corvallis, OR, United States of America; 5 Institute of Biology, Molecular Microbiology and Biotechnology, Leiden University, Leiden, The Netherlands; The University of North Carolina at Chapel Hill, UNITED STATES

## Abstract

The NF-κB-like velvet domain protein VosA (viability of spores) binds to more than 1,500 promoter sequences in the filamentous fungus *Aspergillus nidulans*. VosA inhibits premature induction of the developmental activator gene *brlA*, which promotes asexual spore formation in response to environmental cues as light. VosA represses a novel genetic network controlled by the *sclB* gene. SclB function is antagonistic to VosA, because it induces the expression of early activator genes of asexual differentiation as *flbC* and *flbD* as well as *brlA*. The SclB controlled network promotes asexual development and spore viability, but is independent of the fungal light control. SclB interactions with the RcoA transcriptional repressor subunit suggest additional inhibitory functions on transcription. SclB links asexual spore formation to the synthesis of secondary metabolites including emericellamides, austinol as well as dehydroaustinol and activates the oxidative stress response of the fungus. The fungal VosA-SclB regulatory system of transcription includes a VosA control of the *sclB* promoter, common and opposite VosA and SclB control functions of fungal development and several additional regulatory genes. The relationship between VosA and SclB illustrates the presence of a convoluted surveillance apparatus of transcriptional control, which is required for accurate fungal development and the linkage to the appropriate secondary metabolism.

## Author summary

Velvet domain proteins of filamentous fungi are structurally similar to Rel-homology domains of mammalian NF-κB proteins. Velvet and NF-κB proteins control regulatory circuits of downstream transcriptional networks for cellular differentiation, survival and stress responses. Velvet proteins interconnect developmental programs with secondary metabolism in fungi. The velvet protein VosA binds to more than ten percent of the *Aspergillus nidulans* promoters and is important for the spatial and temporal control of asexual spore formation from conidiophores. A novel VosA-dependent genetic network has been identified and is controlled by the zinc cluster protein SclB. Although zinc cluster proteins constitute one of the most abundant classes of transcription factors in fungi, only a small amount is characterized. SclB is a repression target of VosA and both transcription factors are part of a mutual control in the timely adjusted choreography of asexual sporulation in *A*. *nidulans*. SclB acts at the interphase of asexual development and secondary metabolism and interconnects both programs with an adequate oxidative stress response. This study underlines the complexity of different hierarchical levels of the fungal velvet protein transcriptional network for developmental programs and interconnected secondary metabolism.

## Introduction

Velvet domain transcription factors interconnect fungal developmental programs and secondary metabolism and affect a significant part of differential gene expression during development in comparison to vegetative growth [1]. The majority of the fungal target genes of velvet domain proteins, which bind to promoters of thousands of genes by their Rel homology-like domain, is yet elusive [2,3]. This fungal protein family is highly conserved in ascomycetes and basidiomycetes [4,5].

The velvet proteins VosA (viability of spores A) and VelB (velvet-like B) can form homodimers as well as the VosA-VelB heterodimer to repress or activate gene expression [2,6–9]. VosA represses *brlA* (*br**ist**l**e* *A*) expression encoding a master regulator for the initiation of conidia formation, which are the asexual spores of the fungus. VosA-VelB later activates within conidia the gene encoding the transcription factor VadA (VosA/VelB-activated developmental gene), which downregulates *brlA* expression to allow the maturation of viable conidia [7]. Full suppression of conidiation during vegetative growth of the hyphae require direct binding of VosA and a second *brlA*-repressor, NsdD (never in sexual development D) to the *brlA* promoter [2,8,9]. Growth of fungal filaments after the germination of spores is in the first hours not responsive to external signals, because developmental regulatory genes are not expressed. De-repression of *brlA* accompanies the achievement of developmental competence of fungal hyphae approximately 18 to 20 h post germination [8,10]. This derepression is characterized by delocalization of VosA and NsdD from the *brlA* promoter, which allows the Flb proteins (fluffy low *b**rlA*) FlbB, FlbC, FlbD and FlbE to activate *brlA* expression [8,9,11–15]. A second layer of conidiation repression during vegetative growth is carried out by SfgA (suppressor of *f**lu**G*), which negatively regulates expression of the *flb* genes. FluG (fluffy G) accumulates to a certain threshold during ongoing vegetative growth, which removes the repressive effects of SfgA upon conidiation [16,17].

The Flb proteins activate *brlA* in two distinct cascades: FlbB/FlbE→FlbD→*brlA* and FlbC→*brlA* [11–15,18,19]. The fifth Flb protein, FlbA, regulates development in an indirect manner by antagonizing a G-protein mediated repression of conidiation, and thereby represses vegetative growth [20–22]. The C2H2 transcription factor BrlA activates *abaA* (*aba**cus* *A*) in the mid phase of conidiation [23]. AbaA activates *wetA* (*wet**-white* *A*) in the late phase of conidiation, which is necessary for the synthesis of conidiospore wall components [4,24,25]. VosA is involved in time tuning of conidiation: it represses *brlA* until developmental competence is achieved and is activated by AbaA and WetA downstream of BrlA during late asexual growth [4,26]. VosA regulates conidiospore viability during ongoing spore formation in Aspergilli through activation of genes which products are important for spore maturation [4,6,27–29]. VosA and VelB are important for trehalose biogenesis [4,27]. Trehalose is a storage compound, which supports conidiospore viability and germination [30–32].

Velvet domain proteins couple fungal differentiation programs to specific secondary metabolisms for sexual or asexual development and a fifth of the genome is differentially expressed during development in comparison to vegetative growth [1,33]. Velvet domain proteins are located at the interface between development and secondary metabolism control [33–36]. *A*. *nidulans* is able to produce several secondary metabolites (SMs), such as penicillins, sterigmatocystin, benzaldehydes, emericellamides, orcinol and diorcinol, diindoles, austinol and dehydroaustinol [37–43]. SM genes are often clustered in fungal genomes. Those gene clusters are controlled by cluster-specific transcription factors and master regulators, which interconnect SM biosynthesis and developmental programs in response to environmental cues, such as light [33,41,44,45]. A key element of this interconnection is the velvet complex, comprising the velvet proteins VeA and VelB and the methyltransferase LaeA [27,33,46–50]. Velvet proteins regulate secondary metabolite gene clusters, as well as downstream master regulators, such as the well conserved MtfA (Master transcription factor A) [43,51,52]. Their regulatory versatility suggests a complex hierarchy of multiple control layers of genetic networks mutually controlled by distinct transcription factors.

The zinc cluster (C6) protein SclB acts as activator of a genetic network, which was characterized by genome-wide transcriptional analyses and which represents a novel downstream-target for inhibition of the velvet domain protein VosA in the fungal model organism *A*. *nidulans*. SclB interconnects the formation of asexual spores and the enzymatic as well as non-enzymatic responses upon oxidative stress to a distinct secondary metabolism.

## Results

The DNA-binding velvet protein VosA enriched approximately 1,500 *A*. *nidulans* promoters in chromatin immunoprecipitations combined with whole-genome tiling-oligonucleotide arrays (ChIP-on-CHIP) [2]. One of the VosA controlled regulatory target genes was *AN0585*, which we named SclB, because it corresponds to *A*. *niger scl-2* (*scl**erotia-like* *2*). The *scl-2* mutation was originally generated by UV-mediated random DNA damage and provided this fungus with reduced asexual sporulation, formation of sclerotic-like structures and impaired secondary metabolism [53]. We analyzed the respective *A*. *nidulans* SclB controlled regulatory network located downstream of VosA to explore whether there are connections to developmental programs and secondary metabolism.

**Fig 1:**
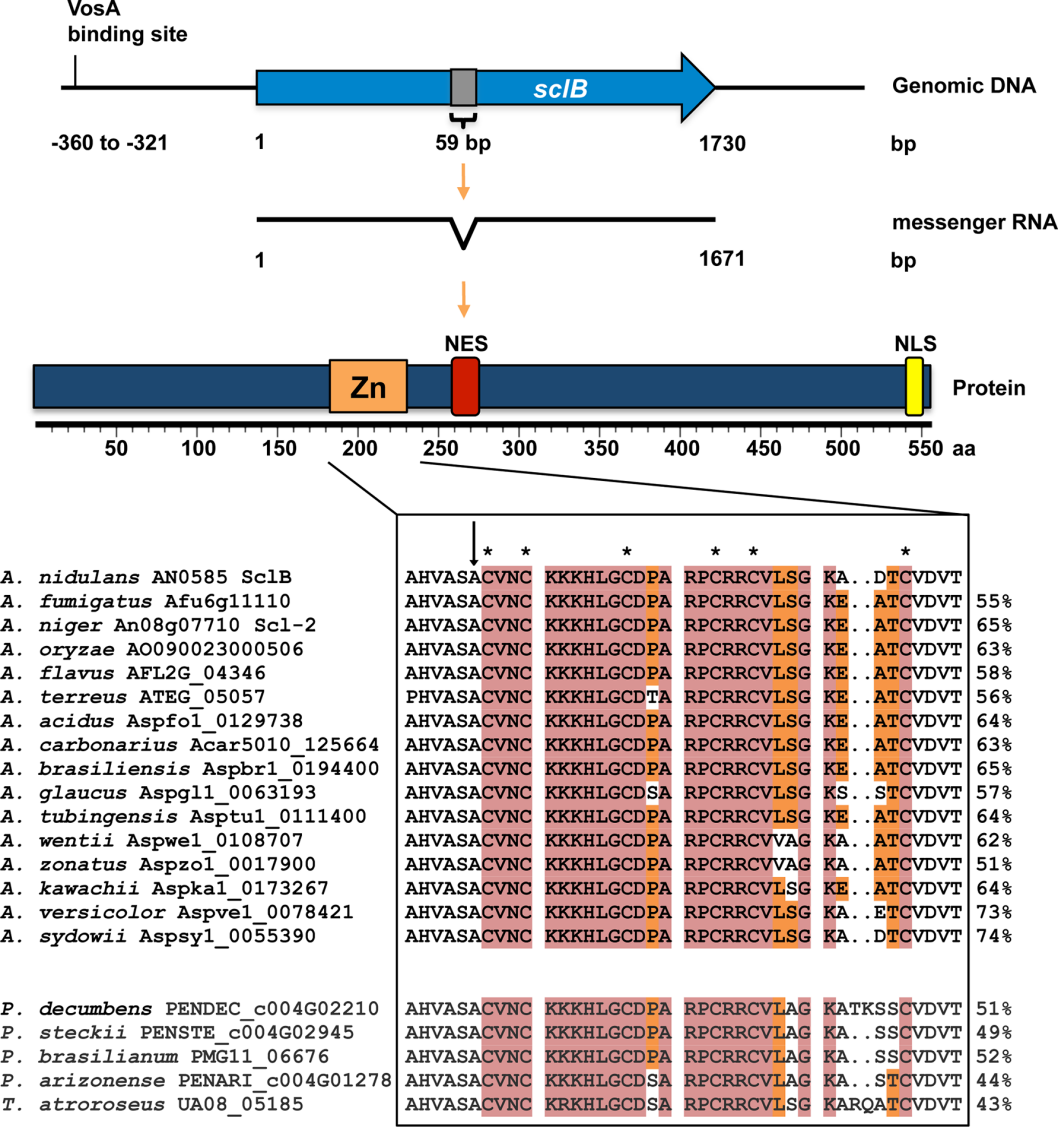
*sclB* encodes the conserved C6 transcription factor SclB. Upper part: Graphical representation of *sclB* (*AN0585*) and its gene product. The VosA binding site [2] is indicated. The grey box represents an intron, bp = base pairs, aa = amino acids, Zn = Zn(II)_2_-Cys_6_ fungal-type DNA-binding domain, NLS = nuclear localization signal, NES = nuclear export site. Lower part: Multiple amino acid sequence alignments of the Zn domain of SclB orthologs from several Aspergilli, *Penicillium* and *Talaromyces* spp. Sequence similarities to SclB from *A*. *nidulans* determined with EMBOSS Needle [54–56] are given on the right side (%). Asterisks mark the cysteine residues of the C6 domain, the arrow indicates the conserved antecedent alanine residue. Red = absolutely conserved among all indicated sequences, orange = conserved in > 50% of indicated sequences.

The SclB *AN0585* open reading frame (ORF) of *A*. *nidulans* comprises 1,730 nucleotides with one intron of 59 nucleotides for a deduced 60 kDa protein of 556 amino acids (Fig 1). SclB showed an amino acid sequence similarity of 65% to *A*. *niger* An08g07710 (Scl-2), 63% to *A*. *oryzae* AO090023000506 and 55% to *A*. *fumigatus* Afu6g11110 (EMBOSS Needle analysis: [54–56]). Orthologs in Aspergilli as well as many fungal families could be identified with high conservation of the C6 domain (BLAST and Phylogeny.fr: [57–59] (Fig 1 and S1 Fig). This conserved C6 domain of SclB (InterProScan [60] in InterPro database [61]) comprises a Zn(II)_2_Cys_6_ zinc cluster fungal-type DNA-binding domain, which is present in one of the most abundant groups of fungal transcription factors [62]. The C6 domain CX_2_CX_6_CX_5_CX_2_CX_8_C architecture of SclB is only found in 19 (approx. 5.7%) out of 332 C6 proteins of *A*. *nidulans* [63,64]. AcuM and ClrB are involved in cellulolytic, iron acquisition and gluconeogenesis pathways and are the only proteins of this architectural group, which have been analyzed so far [65,66]. Most C6 proteins in *A*. *nidulans* exhibit a CX_2_CX_6_CX_6_CX_2_CX_6_C or CX_2_CX_6_CX_5_CX_2_CX_6_C architecture [67].

A nuclear localization signal (NLS) between amino acids positions 541 and 550 (cNLS Mapper: [68]; NucPred: [69]) and a nuclear export sequence (NES) between positions 259 and 273 (LocNES: [70]; NetNES 1.1: [71]) are predicted and support a possible function as a transcriptional regulator.

### SclB influences development in combination with secondary metabolism and stress response

A Δ*sclB* strain was generated to analyze the differences in gene expression in the absence of *sclB* compared to *A*. *nidulans* wildtype. The complete *sclB* ORF in this Δ*sclB* strain was exchanged with a recyclable marker cassette leaving only a small six site as scar (100 nucleotides) after recycling [72].

**Fig 2:**
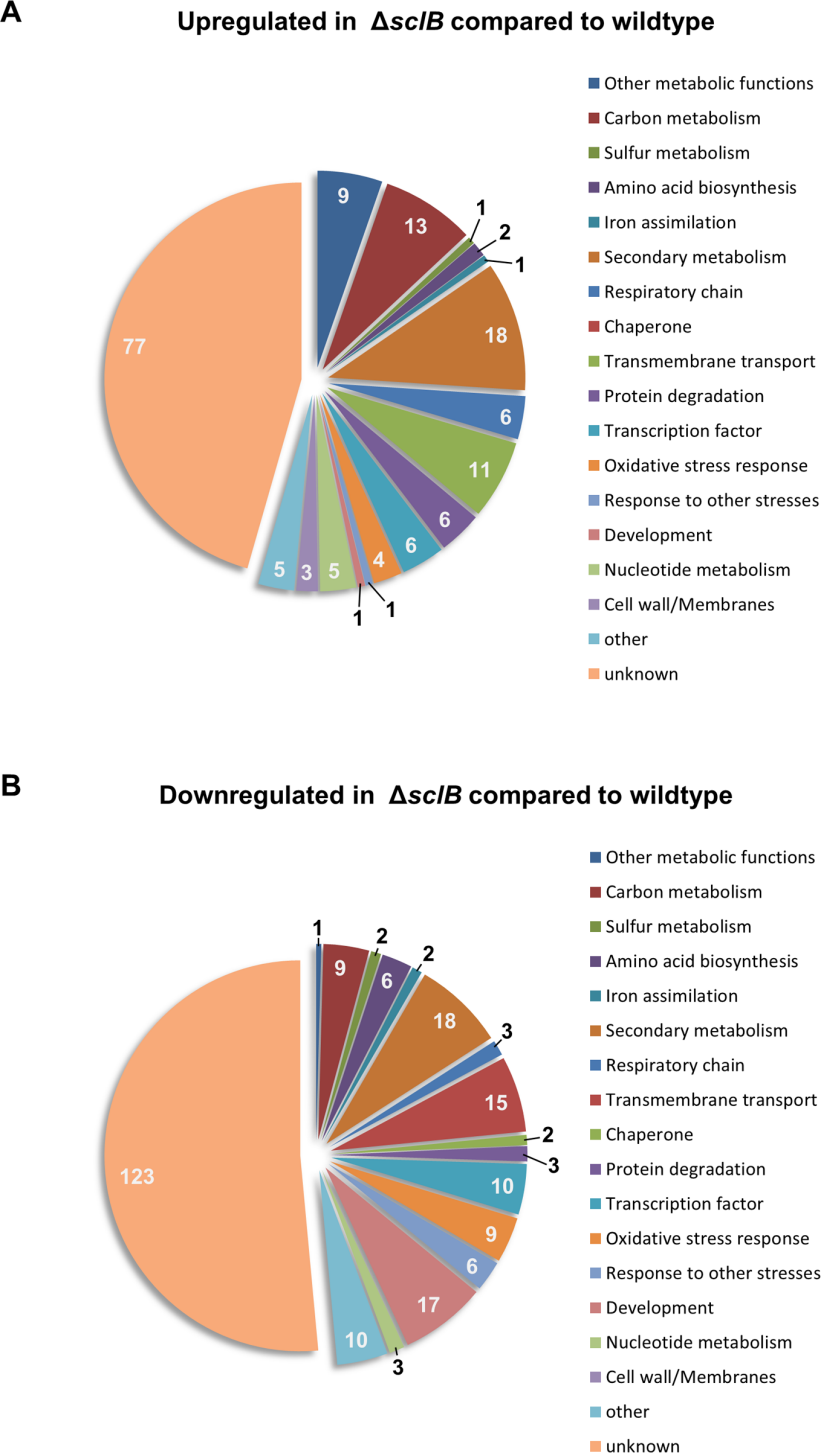
Genome-wide transcriptional analyses of genes influenced by SclB in *A*. *nidulans*. Genes that showed at least two fold change (FC) (Log2 FC of at least 1) in their expression in Δ*sclB* compared to wildtype were divided into the group of A) upregulated (169) and B) downregulated (239) genes. Each group was categorized according to putative functions gathered from the Aspergillus Genome Database (AspGD) [64] and the Fungal and Oomycete Genomics Resources Database (FungiDB) [73]. Raw data can be found in S1 Table. Numbers of genes assigned to respective categories are indicated.

RNA of wildtype, Δ*sclB* and a *sclB* complemented (*sclB* comp) strain were extracted from submerged cultures grown for 24 h under constant agitation and sequenced to compare genome-wide transcriptional changes in the presence or absence of *sclB*. The reintroduction of the *sclB* ORF fully complemented all effects on transcription in the Δ*sclB* strain resulting in transcriptomes comparable to wildtype. 169 genes were significantly increased and 239 were significantly decreased in Δ*sclB* compared to wildtype with a threshold of at least two fold for upregulation or downregulation (Log2 fold change (FC) of at least 1) (S1 Table). Analyses employing the Aspergillus Genome Database (AspGD) [64] and the Fungal and Oomycete Genomics Resources Database (FungiDB) [73] were conducted to categorize these genes into functional groups (Fig 2). 13 genes were assigned to carbon metabolism, one to sulfur metabolism and 9 to other metabolic functions of the genes upregulated in Δ*sclB* compared to wildtype. Genes connected to secondary metabolism constitute the largest group (18) with an assigned function. Several genes related to the respiratory chain (6) or transmembrane transport (11) were also upregulated in Δ*sclB* compared to wildtype. Four genes were assigned to the response to oxidative stress and one is assigned to menadione induced stress. One gene of the group of upregulated genes in Δ*sclB* compared to wildtype is linked to development.

The largest group among the genes downregulated in Δ*sclB* compared to wildtype with an assigned function is related to secondary metabolism (18). Other large groups are constituted of genes connected to development (17) or transmembrane transport (15). Several genes related to carbon metabolism (9), sulfur metabolism (2) or amino acid biosynthesis (6) were found as well amongst the downregulated genes in Δ*sclB* compared to wildtype, as well as genes related to the response to oxidative stress (9) or to other stresses (6).

Members of eight different SM gene clusters were amongst the genes upregulated and 10 amongst the genes downregulated in Δ*sclB* compared to wildtype (Table 1 and S1 Table). This equals approximately 25% of all predicted secondary metabolite gene clusters in *A*. *nidulans* (Table 1 and S1 Table) [74]. Genes encoding backbone enzymes of four of these clusters were upregulated (*AN3396*, *AN3252*, *AN6784* and *AN1242*) and six were downregulated (*AN6236*, *AN9244*, *AN8383 AN2064*, *AN9226* and *AN2924*). This equals approximately 14% of all backbone enzymes of secondary metabolite gene clusters in *A*. *nidulans* [74].

**Table 1:** Secondary metabolite gene cluster members, which expression is influenced by SclB. Genes were assigned to secondary metabolite gene clusters according to a comprehensive secondary metabolite gene cluster annotation published by Inglis and collaborators [74].

Cluster name	Identified members	Regulated in Δ*sclB* / WT
Microperfuranone cluster	*AN3396*, *AN3395*, *AN3394*	*upregulated*
Penicillin cluster	*AN2622*	*upregulated*
pkf cluster	*AN3226*	*upregulated*
pkb cluster	*AN6450*	*upregulated*
AN3252 cluster	*AN3252*, *AN3253*, *AN3254*, *AN3255*	*upregulated*
xptA-containing cluster	*AN6784*	*upregulated*
AN1242 cluster	*AN1242*	*upregulated*
Monodictyphenone cluster	*AN10023*, *AN0146*	*upregulated*
AN6236 cluster	*AN6236*	*downregulated*
AN12331 cluster	*AN7837*	*downregulated*
Austinol cluster 1	*AN9243*, *AN9244*, *AN9253*	*downregulated*
Austinol cluster 2	*AN8383*	*downregulated*
AN2064 cluster	*AN2064*	*downregulated*
AN9226 cluster	*AN9226*	*downregulated*
inp cluster	*AN3502*	*downregulated*
AN2924 cluster	*AN2924*	*downregulated*
Derivative of Benzaldehyde1 and F9775 hybrid cluster 1	*AN7907*	*downregulated*
Emericellamide cluster	*AN2549*	*downregulated*

Taken together, a significant part of the transcriptome is differentially expressed when the Δ*sclB* strain was compared to wildtype, with even 1.5 times more genes with decreased than with increased transcription. Most differentially regulated genes, for which a function could be assigned, are related to secondary metabolism and genes related to development. Another large part of genes differently regulated in the absence of *sclB* compared to the wildtype situation are genes related to stress response, especially of the response towards oxidative stresses.

### *sclB* gene expression accelerates and increases conidiation of *A*. *nidulans*

The *A*. *niger scl-2* mutant forms reduced numbers of conidiophores and structures similar to sclerotia [53], whereas a deletion of the *sclB* orthologous gene in *A*. *fumigatus* (*Afu6g11110*) did not result in any obvious phenotype when grown on minimal medium (S2 Fig). Transcriptomic analyses of the Δ*sclB* strain compared to wildtype in *A*. *nidulans* suggested that SclB is involved in asexual development (Fig 2 and S1 Table).

**Fig 3:**
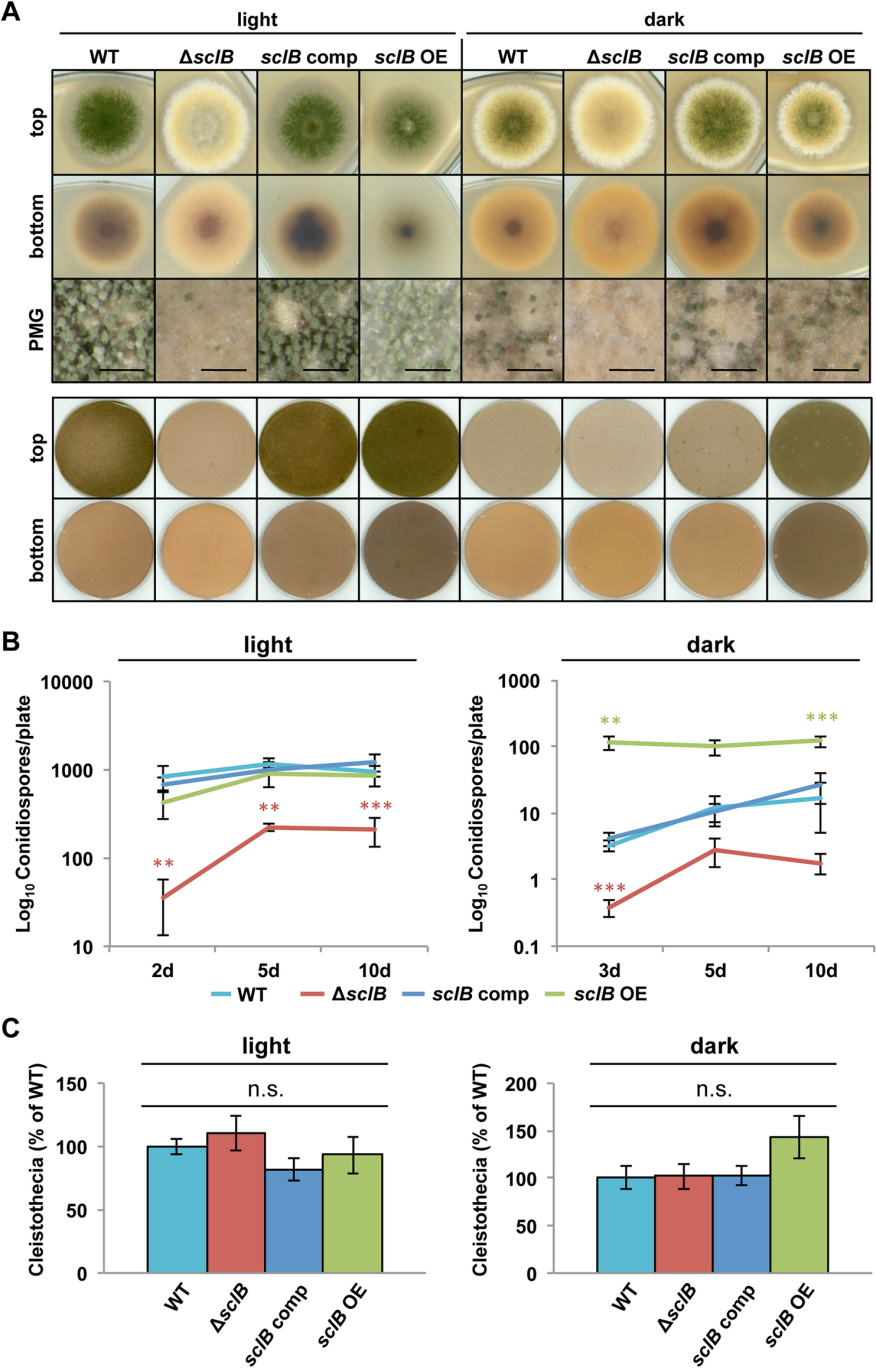
*sclB* accelerates and increases conidiation of *A*. *nidulans*. A) Comparison of wildtype *A*. *nidulans* (WT), deletion of *sclB* (Δ*sclB*), *sclB* complemented (comp) by reintroducing the *sclB* ORF into Δ*sclB* and overexpressed (*sclB* OE) strains, which were point inoculated (upper part) or plated (lower part) and grown under asexual (light) or sexual (dark) inducing conditions for 3 d. PMG = photomicrograph, black bars = 200 μm. B) The same strains were plated and grown in light or dark for up to 10 d. Conidiospore numbers per plate were determined after 2 or 3, 5 and 10 d of growth (**P<0.005, ***P<0.001). C) Cleistothecia of indicated strains were quantified from plated cultures after 8 d grown in light (left hand side) or dark (right hand side). Cleistothecia amounts formed by WT were set to 100%, n.s. = not significant.

The growth and differentiation of the Δ*sclB* mutant strain was examined during light and unlimited oxygen supply promoting asexual spore formation in comparison to cultivation in dark with limited oxygen supply supporting sexual development (Fig 3A). *A*. *nidulans* wildtype forms high numbers of conidiophores carrying asexual spores in light and produces lower numbers of asexual spores in dark after a delay of several days [1]. The absence of *sclB* leads to a significantly decreased formation of conidiophores during asexual or sexual development, compared to wildtype (Fig 3A and 3B). This phenotype of the *A*. *nidulans* Δ*sclB* strain was fully restored by re-introducing either the *sclB* ORF into Δ*sclB* (*sclB* comp) or the *sclB* ortholog from *A*. *fumigatus* (*Afu6g11110*) sharing 55% similarity, indicating functional conservation (S2 Fig).

Quantification of conidiospore formation in light revealed that the Δ*sclB* strain produced less than 5% of the asexual spores produced by the wildtype after two days and reached a maximum of approximately 20% of the wildtype conidia after 10 days. *A*. *nidulans* reduces conidiophore formation during growth in the dark and favors cleistothecia formation. The Δ*sclB* strain produced significantly less conidiospores during growth in the dark in comparison to light suggesting that light control of development is independent of SclB. Overexpression of *sclB* (*sclB* OE) under control of a nitrate-inducible promoter (^P^*niaD*) further increases asexual spore formation in the dark, when the wildtype produced only low amounts of conidia (Fig 3A).

Sexual development includes nest formation and the differentiation of cleistothecia as closed fruiting bodies, which is increased in the dark and reduced in light. Cleistothecia formation is similar in the Δ*sclB* strain in comparison to wildtype and additional control strains suggesting that SclB control is rather targeting asexual than sexual development (Fig 3C).

The *sclB* OE strain increased the production of conidiophores significantly when grown under inhibiting and delaying conditions in the dark under limited oxygen supply, when the wildtype only produced small amounts of conidiophores and the formation of cleistothecia is favored (Fig 3). This effect in the *sclB* OE strain is even more pronounced when instead of point inoculated colonies leading to radial zones of different ages [75]; (Fig 3A upper part), plated colonies emerging from separated germinating spores were monitored. Plated colonies form a coherent mycelium due to hyphal fusion through anastomosis tubes, and are of same age at every spot (Fig 3A lower part, Fig 3B and 3C) [76,77].

These data indicate that SclB is required for significant, efficient and accelerated conidiophore formation of *A*. *nidulans*.

### *sclB* gene expression is repressed by VosA

**Fig 4:**
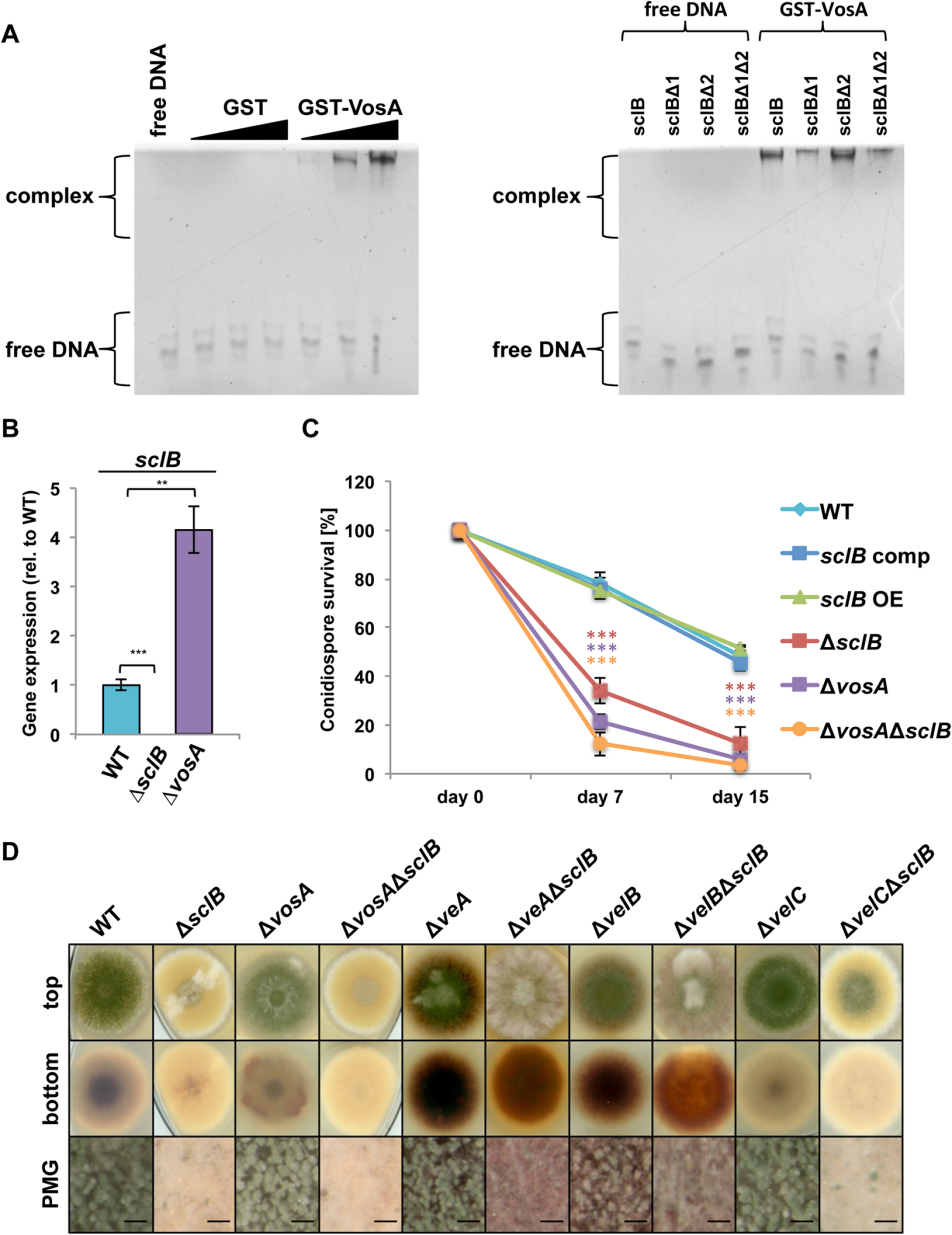
VosA binds to the *sclB* promoter and represses transcription of the gene. A) Electrophoretic mobility shift assay (EMSA) employing GST-VosA with the 40 base pair (bp) probe of the VosA binding motif upstream of *sclB* (left side). DNA and protein were used in molar ratios of 1:0.3, 1:1 and 1:3. Protein-DNA complex formation of GST-VosA and the DNA probe indicate VosA binding to this region upstream of *sclB*. Free DNA and free GST are shown as negative controls. GST-VosA specifically binds nine bps in the proposed *sclB* promoter region (right side). Two putative VosA binding motifs were identified in the 40 bp probe of the *sclB* promoter region. DNA probes missing either region (sclBΔ1 or sclBΔ2) or both regions (sclBΔ1Δ2) showed that VosA specifically binds to region 1, spanning bps -337 to -329 in front of the *sclB* open reading frame. Same amounts of protein and respective DNA probe were used in each lane. One DNA probe per lane was used. B) *sclB* is upregulated in the absence of *vosA* in asexually grown colonies. RNA was extracted from cultures grown under submerged culture conditions for 24 h on a rotary shaker at 37°C and subsequently shifted on solid MM plates and grown for 24 h in light to induce asexual development. Expression of *brlA* in WT was set to 1; normalized against expression of the three reference genes *h2A*, *15S rRNA* and *gpdA* (** *P*<0.005, *** *P*<0.001). C) Conidiospores show a rapid loss in viability in the absence of *vosA* or *sclB*. 200 spores per strain were plated after zero, seven and 15 d and initial spore forming units were set to 100%. Error bars represent standard error of means of n ≥ 5 biological replicates (*** *P*<0.001). D) *sclB* is epistatic towards *vosA*. Single and double knock out mutants of *sclB* and the *velvet* genes were constructed. Strains were point inoculated and grown for 3 d in light. Black bars = 200 μm, PMG = photomicrograph.

ChIP-on-Chip experiments showed that VosA binds the *sclB* promoter *in vivo* approximately 311 bp upstream of the *sclB* ORF [2]. Promoter walking electrophoretic mobility shift assays (EMSAs) revealed that VosA binds a 40 bp region of the *sclB* promoter (marked in Fig 1). EMSAs of this region and purified VosA protein verified dosage-dependent VosA binding *in vitro* (Fig 4A). In the EMSA protein-DNA complexes run high in the gels and free DNA runs in the lower part. Possible formation of GST-VosA dimers might lead to binding of more than one DNA molecule at the same time. Two putative binding sequences were identified in this region and mutations for both of them, in which the respective putative binding sequence was deleted, showed that VosA specifically binds nine bps, spanning -337 to -329 in front of the *sclB* ORF (Fig 4A). A *vosA* deletion mutant (Δ*vosA*) was constructed to analyze the impact of VosA upon *sclB* gene expression. Transcription levels of *sclB* were monitored in wildtype and Δ*vosA* strain with quantitative real-time PCR (qRT-PCR). *sclB* transcription is upregulated in the absence of *vosA* in asexually grown colonies 24 h post induction of development (Fig 4B). This indicates a repressing effect of VosA towards *sclB* expression during asexual development. This is in accordance with transcriptomic data showing an upregulation of *sclB* gene expression in conidiospores of a Δ*vosA* strain in comparison to wildtype published by Park and co-workers [78].

AbaA and WetA activate *vosA* during late asexual development. VosA together with VelB is necessary for trehalose biogenesis to support spore viability [4,6]. Spore viability was compared in Δ*sclB* and *sclB* OE strains on solid minimal medium. Conidiospores of the Δ*sclB* strain showed a rapid loss in spore viability compared to spores of wildtype, *sclB* comp and *sclB* OE strains after seven days and thereafter (Fig 4C). A similar loss in spore viability was found for the Δ*vosA* strain, whereas conidiospores of the Δ*vosA*Δ*sclB* double mutant strain showed further diminished viability after seven days and thereafter.

The Δ*vosA* single mutant produces grey-greenish conidiospores with decreased viability [4] (Fig 4D). The Δ*vosA*Δ*sclB* double deletion strain supports an epistatic interaction of *sclB* towards *vosA*, because it showed the Δ*sclB* single mutant phenotype of reduced conidia formation with low spore viability (Fig 4C and 4D). These findings place the gene encoding SclB genetically downstream of the gene for VosA. VosA binds upstream of *sclB* and represses *sclB* gene expression.

VosA acts as homodimer or forms with VelB or VelC the heterodimers VosA-VelB or VosA-VelC [6,79], which fulfill different functions in fungal development and interconnected secondary metabolism. Double deletions of *sclB* and *velB* or *velC*, respectively, were created to discriminate between SclB functions downstream of the VosA-VosA homodimer or the VosA-VelB and VosA-VelC heterodimers. *veA* was included into these analyses, because VeA competes with VosA for VelB and forms the VeA-VelB heterodimer. The Δ*veA* and Δ*velB* single mutants are unable to form cleistothecia on minimal medium and are misregulated in secondary metabolism producing dark reddish pigments [6,33,52] (Fig 4D). The Δ*sclB*Δ*veA* and Δ*sclB*Δ*velB* double mutants both show additive phenotypes with impaired asexual and sexual development. The loss of cleistothecia formation of the Δ*veA* and Δ*velB* single mutant is combined with increased amounts of aerial hyphae without conidia and significantly smaller greenish colony centers representing conidiophores. This indicates a SclB function for conidiophores independently of the VeA or VelB governed pathways for fruiting bodies and the corresponding secondary metabolism. The Δ*velC* single mutant shows an almost wildtype-like phenotype on minimal medium combined with increased amounts of conidiophores [79]. The Δ*sclB*Δ*velC* double deletion strain shows an intermediate phenotype with a colony similar to the Δ*sclB* phenotype combined with an increased greenish colony center for conidiophores. Therefore, SclB functions independently of the velvet protein heterodimers VosA-VelB or VosA-VelC and is primarily a repression target of the VosA homodimer.

### SclB activates conidiation through regulation of *brlA* gene expression

**Fig 5:**
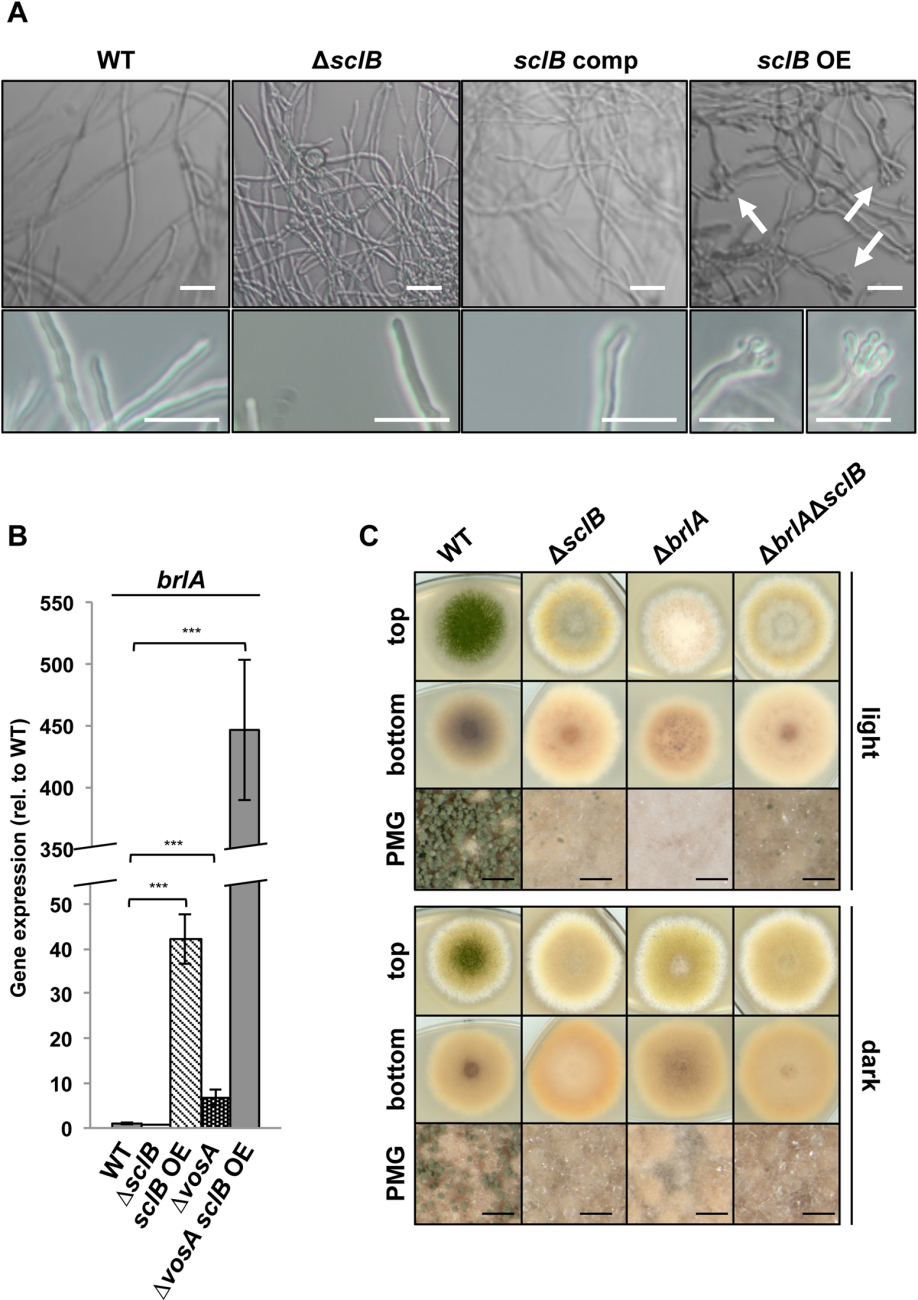
SclB is a major regulator of asexual development. A) Photomicrographs of vegetatively grown wildtype (WT), Δ*sclB*, *sclB* comp and *sclB* OE strains. Strains were grown for 18 h in submerged cultures on a rotary shaker at 37°C. White arrows indicate conidiophores. White bars = 20 μm. B) Relative gene expression of *brlA* in WT, Δ*sclB*, *sclB OE*, Δ*vosA*, Δ*vosA* in *sclB* OE background during vegetative growth determined by qRT-PCR. Expression of *brlA* in WT was set to 1; normalized against *h2A*, *15S rRNA* and *gpdA* expression (*** *P*<0.001). Strains were grown for 24 h in submerged cultures. The axis of ordinates is non-continuous to provide visibility of values below 50. C) WT, Δ*brlA*, Δ*sclB* and the double mutant strains were point inoculated and grown for 3 d in light or dark at 37°C. The shown phenotypes support that *sclB* is epistatic to *brlA*, and that the SclB protein acts upstream of BrlA.

SclB functions downstream of VosA and its absence leads to decreased conidiophore formation, whereas the *sclB* OE strain produces increased numbers of conidiophores during sexual development. This indicates that SclB is an activator of conidiophore formation. Strains were grown in liquid minimal medium to test whether an overexpression of *sclB* is sufficient to induce development under vegetative conditions. Growth in submerged cultures suppresses development in *A*. *nidulans* and results in solely vegetative growth of the wildtype (Fig 5A). No conidiophores were found in wildtype, Δ*sclB* or *sclB* comp strains grown in submerged cultures. In contrast, the *sclB* OE strain forms conidiophores after 18 h of growth in submerged cultures (Fig 5A).

VosA represses gene expression of the master regulator-encoding *brlA*, and a Δ*vosA* strain forms conidiophores when grown in submerged culture conditions [4]. The expression of *brlA* was examined in the *sclB* OE mutant during vegetative growth. Strains were grown under submerged conditions what hinders asexual development in the wildtype. The wildtype only expresses basal levels of *brlA* under these conditions. In contrast, mRNA levels of *brlA* are highly upregulated in the presence of high amounts of SclB in the *sclB* OE strain (Fig 5B). VosA represses *brlA* during vegetative growth and *brlA* gene expression was upregulated in the Δ*vosA* strain grown under submerged culture conditions as well (Fig 5B) [4,8]. Expression of *brlA* in a Δ*vosA* mutant in the *sclB* OE background was tested to examine, whether SclB is able to activate *brlA* gene expression. Whereas *brlA* expression was already upregulated about 40 times in *sclB* OE compared to wildtype, the Δ*vosA sclB* OE mutant showed even more than 400 times upregulation compared to wildtype (Fig 5B). This additional upregulation indicates that SclB is able to activate *brlA* expression in the absence of *vosA*.

Activation of the conidiation pathway is inhibited by the repressors VosA and NsdD during vegetative growth, which are released from the *brlA* promoter when the fungus becomes developmentally competent [4,8,9]. SfgA represses conidiation indirectly by regulating the genes for the Flb factors [16,80]. Expression levels of *sfgA*, *nsdD* and *vosA* were analyzed by qRT-PCR in *sclB* mutant strains to exclude the possibility that SclB influences the conidiation pathway by downregulating gene expression of these repressors (S3A Fig). Gene expression of none of these repressor genes is altered in Δ*sclB* or *sclB* OE strains in comparison to wildtype. This demonstrates that SclB does not control the conidiation pathway through repression of its repressor genes. Taken together, the presented data indicate that SclB is an activator of the conidiation pathway through the *brlA* activator gene.

The Δ*brlA* bristle mutant phenotype of primarily stalks with diminished conidia (Fig 5C) is distinctly different from the Δ*sclB* phenotype. The Δ*sclB*Δ*brlA* double mutant resembles the Δ*sclB* single mutant, supporting an epistasis of *sclB* towards *brlA* (Fig 5C). This underlines a function of SclB upstream of *brlA* in developmental programs. In addition, epistasis of *sclB* and *abaA*, a downstream factor of *brlA* [81], was analyzed. Δ*abaA* forms brownish conidiophores with intermittent tumefactions, which are distinctly decreased in number [82] (S3B Fig). The Δ*sclB*Δ*abaA* mutant shows the Δ*sclB* single mutant phenotype but has lost the greenish colony center (S3B Fig). This shows that *sclB* is epistatic to *abaA* and corroborates the finding that SclB activates the conidiation cascade upstream of its major regulator BrlA.

### SclB activates the conidiation pathway at *brlA* and several upstream regulatory control genes

**Fig 6:**
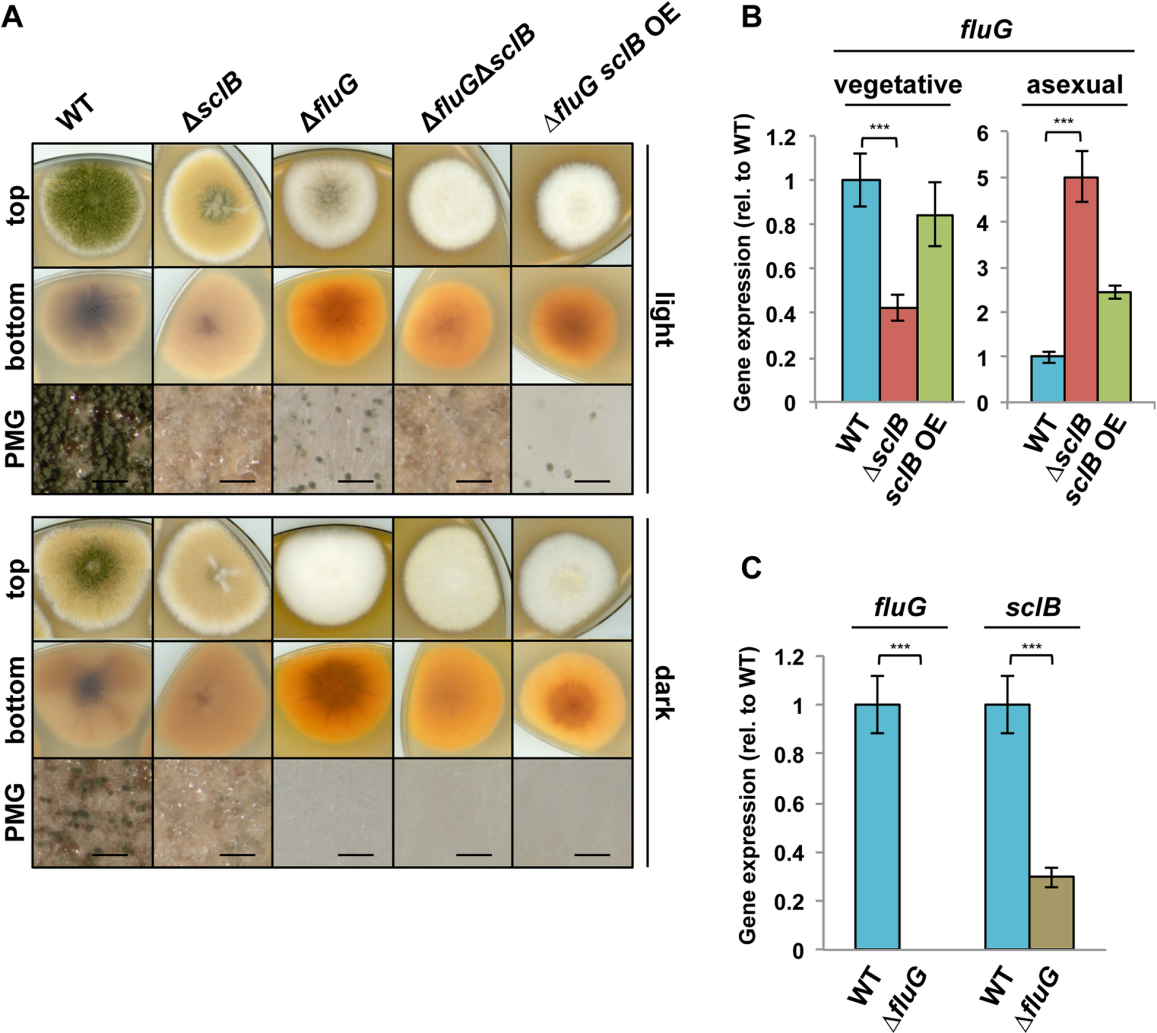
SclB functions downstream of FluG. A) Indicated strains were point inoculated and grown for 3 d in light or dark at 37°C. *sclB* OE does not rescue Δ*fluG* suggesting that *fluG* is epistatic to *sclB*. B) qRT-PCR of RNA, which was extracted from cultures grown under submerged conditions on a rotary shaker at 37°C and harvested after 24 h (vegetative) or subsequently shifted onto solid MM plates and grown for 24 h at 37°C in light (asexual). The *fluG* expression is downregulated in the absence of *sclB* during vegetative growth and upregulated during asexual growth (*** *P*<0.001). C) qRT-PCR of vegetatively grown cultures of Δ*fluG* shows that *sclB* expression is decreased in the absence of *fluG*. Normalized against *h2A*, *gpdA* and *15s rRNA* (*** *P*<0.001).

An increased *brlA* expression directly leads to spore formation from vesicle-like structures [83], whereas *sclB* OE activating *brlA* expression forms conidiophores under submerged culture conditions. Upstream activators of *brlA* were analyzed to examine whether SclB activates further regulatory genes of asexual development upstream of *brlA*. FluG is a key upstream activator of the conidiation pathway and acts as a time-dependent repressor of the conidiation-repressor SfgA [8,16,17]. The deletion of *fluG* leads to drastically reduced conidiation and a fluffy whitish phenotype with low amounts of conidiophores and high amounts of aerial hyphae [17] (Fig 6A). The back of the colony shows a light orange color indicating an alteration in secondary metabolite production. *sclB* was knocked out in the Δ*fluG* strain to analyze epistatic interactions. The Δ*fluG*Δ*sclB* double mutant strain shows an additive phenotype with large amounts of aerial hyphae, but completely failed to produce conidiophores (Fig 6A). In addition, the orange color was less bright. The Δ*fluG* phenotype was not rescued by an overexpression of *sclB* (Fig 6A). This indicates a function of the SclB protein downstream of FluG or the FluG-SfgA pathway. The *sclB* gene is presumably not a direct downstream target of FluG-mediated gene activation, as *sclB* OE could not rescue the loss of *fluG*. Transcription of *fluG* was increased in qRT-PCR analyses from vegetatively grown *ΔsclB* strain (Fig 6B). This corroborates that SclB does not function as activator of *fluG* gene expression. SclB might have repressing effects upon *fluG* expression during late asexual development (spore maturation), because *fluG* expression is upregulated in the absence of *sclB* during asexual growth after 24 h in comparison to wildtype (Fig 6B). The *sclB* gene expression is decreased in the absence of *fluG* as well, suggesting regulatory feedback loops or cross talk between both factors and their corresponding genes (Fig 6C).

**Fig 7:**
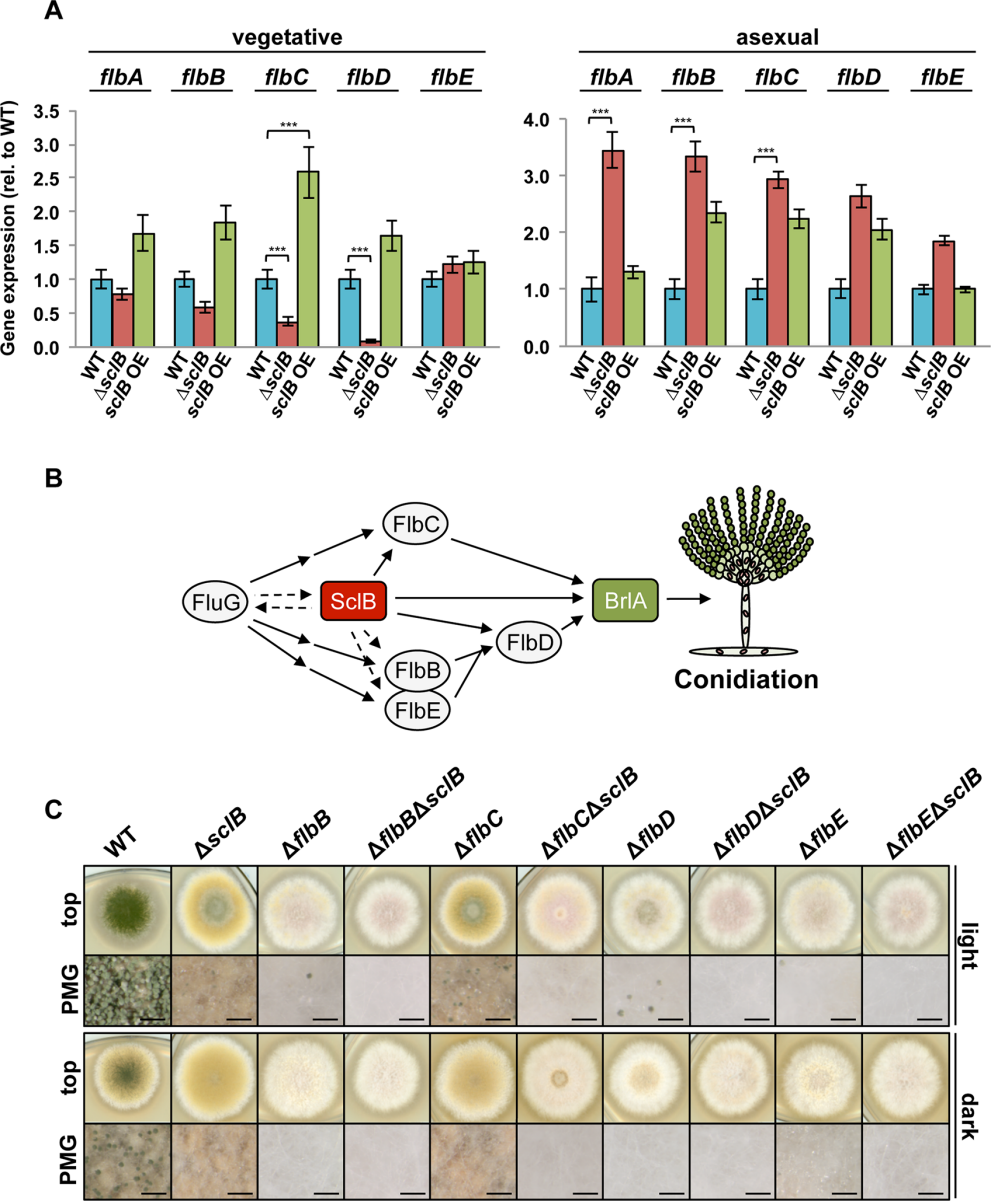
SclB regulates gene expression of early conidiation-activator encoding *flbC* and *flbD*. A) qRT-PCR shows that *flbC* gene expression is downregulated in the absence of *sclB* and upregulated in the *sclB* OE strain during submerged vegetative growth (left). *flbD* gene expression is downregulated in Δ*sclB* as well. Expression of *flbA*, *flbB* and *flbC* is significantly upregulated in Δ*sclB* during growth on solid MM in light (right) (***P<0.001). B) Relationship between upstream factors of BrlA in the conidiation pathway. SclB activates *flbC*, *flbD* and *brlA* gene expression. C) Single and double mutants of the *flb* genes and *sclB* were point inoculated and grown for 3 d in light or dark. Δ*flbC* and Δ*sclB* show similar phenotypes. All Δ*flb*Δ*sclB* mutants fail to form conidiophores, as shown in photomicrographs (PMG). Black bars = 200 μm.

The Flb factors, which act downstream of FluG, activate *brlA* in two cascades: FlbB/FlbE→FlbD→BrlA and FlbC→BrlA [11–15,18,80] (Fig 7). Genome-wide transcriptional ana-lysis showed that *flbC* and *flbD* transcript levels are significantly lower in Δ*sclB* compared to wildtype during late vegetative growth when the fungus reached the state of developmental competence (S1 Table). Transcription of *flbB–E* was analyzed in more detail through qRT-PCR measurements. *flbD* gene expression is distinctly lower in submerged cultures in the absence of *sclB* compared to wildtype (Fig 7). Moreover, *flbC* is downregulated in Δ*sclB* after 24 h of vegetative growth in submerged cultures, but upregulated in the *sclB* OE strain, compared to wildtype. This is in agreement with the data obtained in genome-wide transcriptomics (S1 Table). Transcription of *flbB* and *flbE* is not significantly differentially regulated in the *sclB* mutants compared to wildtype in qRT-PCR analyses. Nevertheless, expression profiles of both, *flbB* and *flbE* in *sclB* mutants resemble these of *flbC* and *flbD* in their tendencies, indicating regulatory effects of SclB upon these factors as well. These analyses suggest an activating role of SclB towards the Flb cascade upstream of *brlA* and specifically towards *flbC* and *flbD* during late vegetative growth at the onset of conidiation.

Transcription of *flbB*, *flbC* and *flbD* is upregulated in the absence of *sclB* compared to wildtype after 24 h of asexual growth. Similarly, the *flbA* gene for an RGS (Regulator of G protein Signaling) domain protein indirectly supporting conidiation [84], is upregulated during asexual growth in the absence of *sclB* but not during vegetative growth. These findings indicate that SclB regulation of the conidiation cascade is part of a timely adjusted choreography of asexual development.

Single and double knock out strains of the *flb* genes were created to further investigate the genetic relationship between *sclB* and the *flb* genes. All *flb* single deletions showed fluffy phenotypes [85] that are distinctly different to the Δ*sclB* phenotype (Fig 7C). Only Δ*flbC* is an exception with a phenotype similar to Δ*sclB*, which is in agreement with the finding that SclB activates *flbC* gene expression. Double deletions of *sclB* and each of the *flb* genes showed phenotypes with a complete abolishment of conidiophores (Fig 7C). The Δ*flbC*Δ*sclB* strain resembles the phenotypes of the other Δ*flb*Δ*sclB* strains, indicating that SclB functions upstream of both parts of the Flb cascade and underlines the finding that SclB activates *flbC* and *flbD*. *sclB* OE is not sufficient to restore the wildtype phenotype in *flb* knock out strains, showing that SclB acts upstream of the Flb factors (S4 Fig). Taken together, these findings demonstrate that SclB activates not only *brlA* but also both Flb cascades through the activation of *flbC* and *flbD*, which both merge and further activate *brlA*.

### SclB regulates emericellamides, austinol and dehydroaustinol secondary metabolite production

Genome-wide analysis of SclB’s influence on gene expression suggests that approximately 25% of all SM gene clusters in *A*. *nidulans* are misregulated in the absence of *sclB* compared to wildtype (Table 1 and S1 Table). The SclB-regulated interconnection of asexual development and secondary metabolism was examined in more detail by comparing SMs from *sclB* mutant and wildtype strains. Extracellular SMs were extracted with ethyl acetate from wildtype and the *sclB* mutant strains either grown for 48 h vegetatively or three and seven days under conditions inducing asexual or sexual development in wildtype.

**Fig 8:**
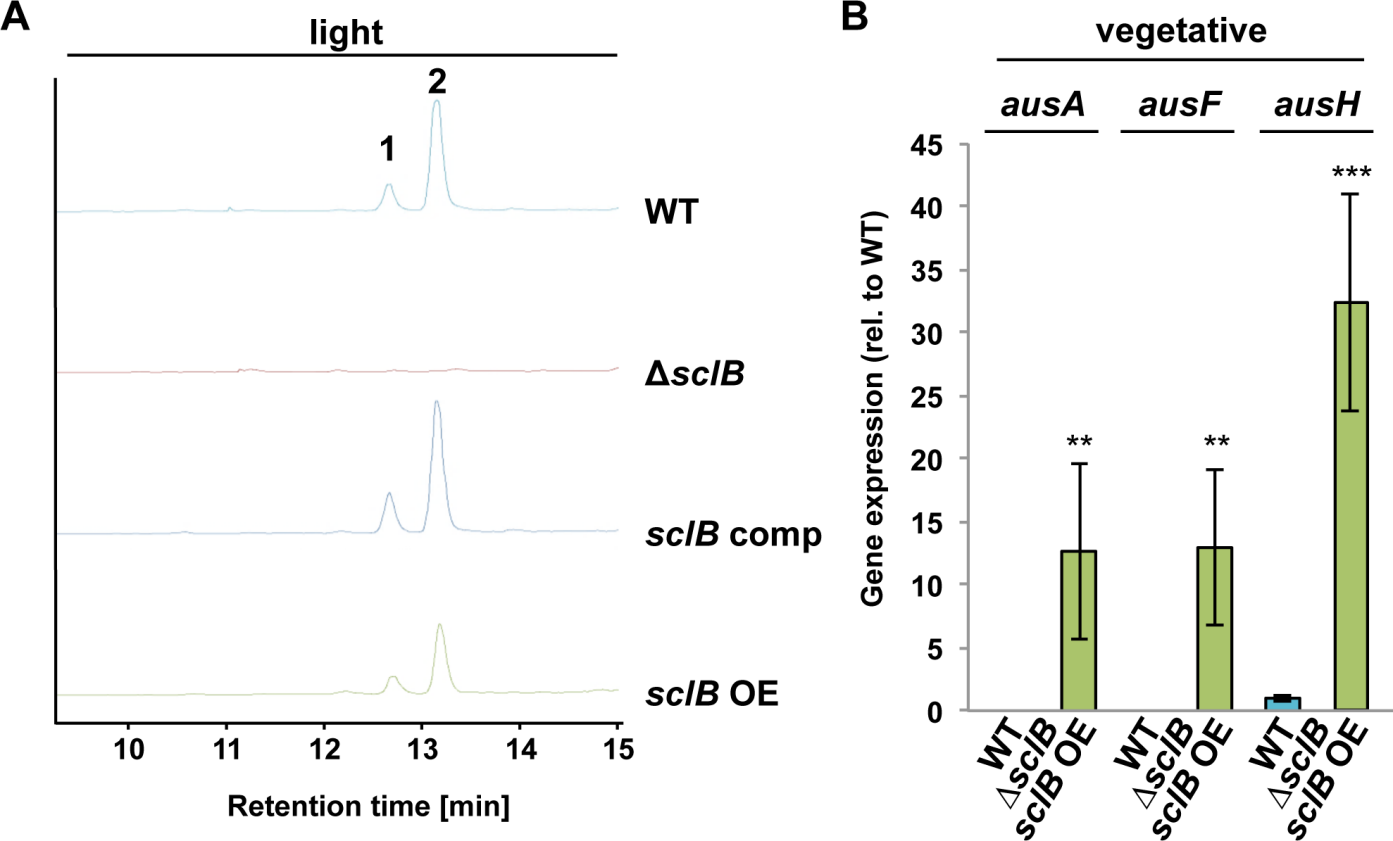
SclB regulates austinol and dehydroaustinol production. A) Wildtype (WT), the *sclB* comp and *sclB* OE strains produce austinol (1) and dehydroaustinol (2) during asexual growth as determined by high performance liquid chromatography (HPLC). Both compounds are absent in samples from Δ*sclB*. Secondary metabolites were extracted from cultures grown for 3 days in light on solid MM plates at 37°C. Employed detector = ELSD B) qRT-PCR shows that *sclB* OE upregulates *ausA*, *ausF* and *ausH* gene expression during submerged growth. *ausA* and *ausF* are not expressed in WT and none of the three tested genes is expressed in Δ*sclB* during these growth conditions (**P<0.005, ***P<0.001).

High-performance liquid chromatography (HPLC) revealed that the wildtype as well as the *sclB* OE strain, but not the Δ*sclB* strain, produce austinol and dehydroaustinol after three and seven days of asexual growth in light. Both compounds were identified in samples extracted from wildtype, the *sclB* complemented strain and the *sclB* OE strain according to their masses and UV/VIS absorption maxima (Figs 8A and S5) [86]. *ausA*, coding for a polyketide synthase producing the intermediate 3,5-dimethyl orsellinic acid, and *ausF*, required for the synthesis of both austinol and dehydroaustinol [39] are not expressed during vegetative growth in wildtype and Δ*sclB*, but in the *sclB* OE strain (Fig 8B). A third SM producing gene *ausH*, which is necessary for austinol and dehydroaustinol production, was basally expressed in wildtype, but not in Δ*sclB*, whereas the *sclB* OE strain showed upregulation of *ausH* transcription (Fig 8B). This is in accordance with transcriptomic data indicating that backbone enzymes of both austinol clusters are downregulated in the absence of *sclB* compared to wildtype (Table 1 and S1 Table). This indicates that SclB activates expression of the austinol gene cluster during vegetative growth.

**Fig 9:**
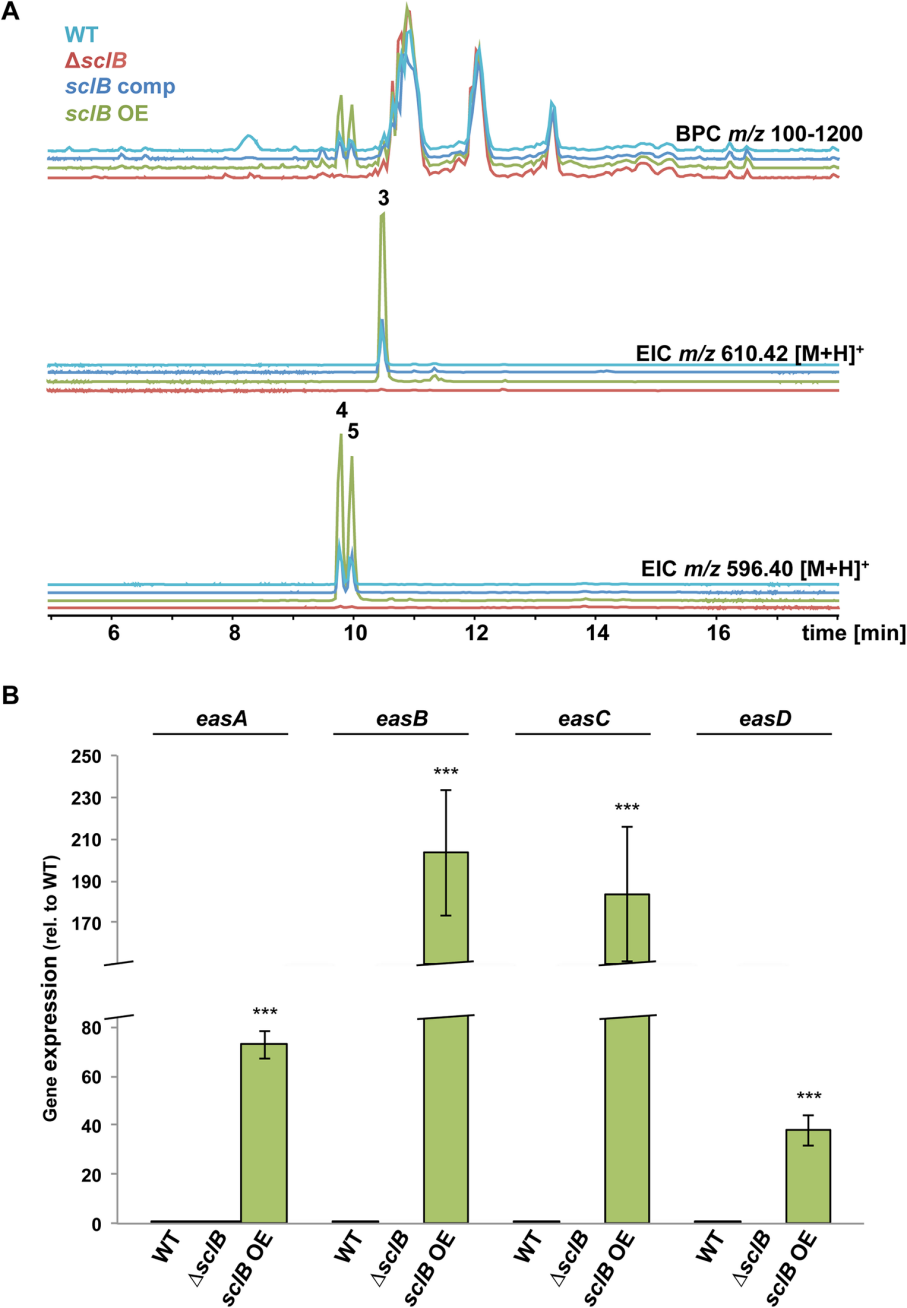
Overexpression of *sclB* leads to increased production of emericellamides and upregulation of emericellamide cluster genes. A) Liquid chromatography coupled to mass spectrometry reveals that the *sclB* OE strain exhibits increased production of emericellamide A (3), C (4) and D (5) compared to wildtype (WT) and *sclB* comp during vegetative growth. The Δ*sclB* strain only produces traces of these emericellamides. Depicted is the base peak chromatogram (BPC, upper part) and extracted ion chromatograms (EIC, lower part) of *m/z* 610.42 [M+H]^+^ (3) and *m/z* 596.40 [M+H]^+^ (4 and 5), respectively. Strains were grown for 48 h in submerged culture conditions and extracellular secondary metabolites were extracted with ethyl acetate. B) qRT-PCR shows that *sclB* OE upregulates *easA*, *easB*, *easC* and *easD* gene expression in comparison to wildtype (WT) during vegetative growth (*** *P*<0.001). None of these genes is expressed in Δ*sclB* under these culture conditions, with the exception of *easA*, which is basally expressed.

HPLC coupled to a qToF mass spectrometer revealed that the *sclB* OE strain produces increased amounts of emericellamide A, C and D [87] during vegetative growth (Figs 9A and S6). The Δ*sclB* strain produces only traces of these compounds under tested growth conditions and no fragmentation for emericellamide A and D could be obtained from mass spectrometry (Fig 9A and S6). Expression of the four genes of the emericellamide gene cluster, *easA* to *easD*, was analyzed in vegetatively grown cultures. *easA* and *easD* are basally expressed in wildtype. Only *easA*, but not *easB*, *easC* or *easD*, was basally expressed in the Δ*sclB* strain. In contrast, all four genes are upregulated in *sclB* OE (Fig 9B). Furthermore, *easD* was significantly downregulated in genome-wide transcriptomic analysis in the absence of *sclB* compared to wildtype (S1 Tab). This shows that SclB acts as activator of the *eas* gene cluster and is necessary for emericellamide biosynthesis.

Taken together, SclB activates the expression of SM clusters for emericellamides, austinol and dehydroaustinol during vegetative growth.

### SclB activates the oxidative stress response

**Fig 10:**
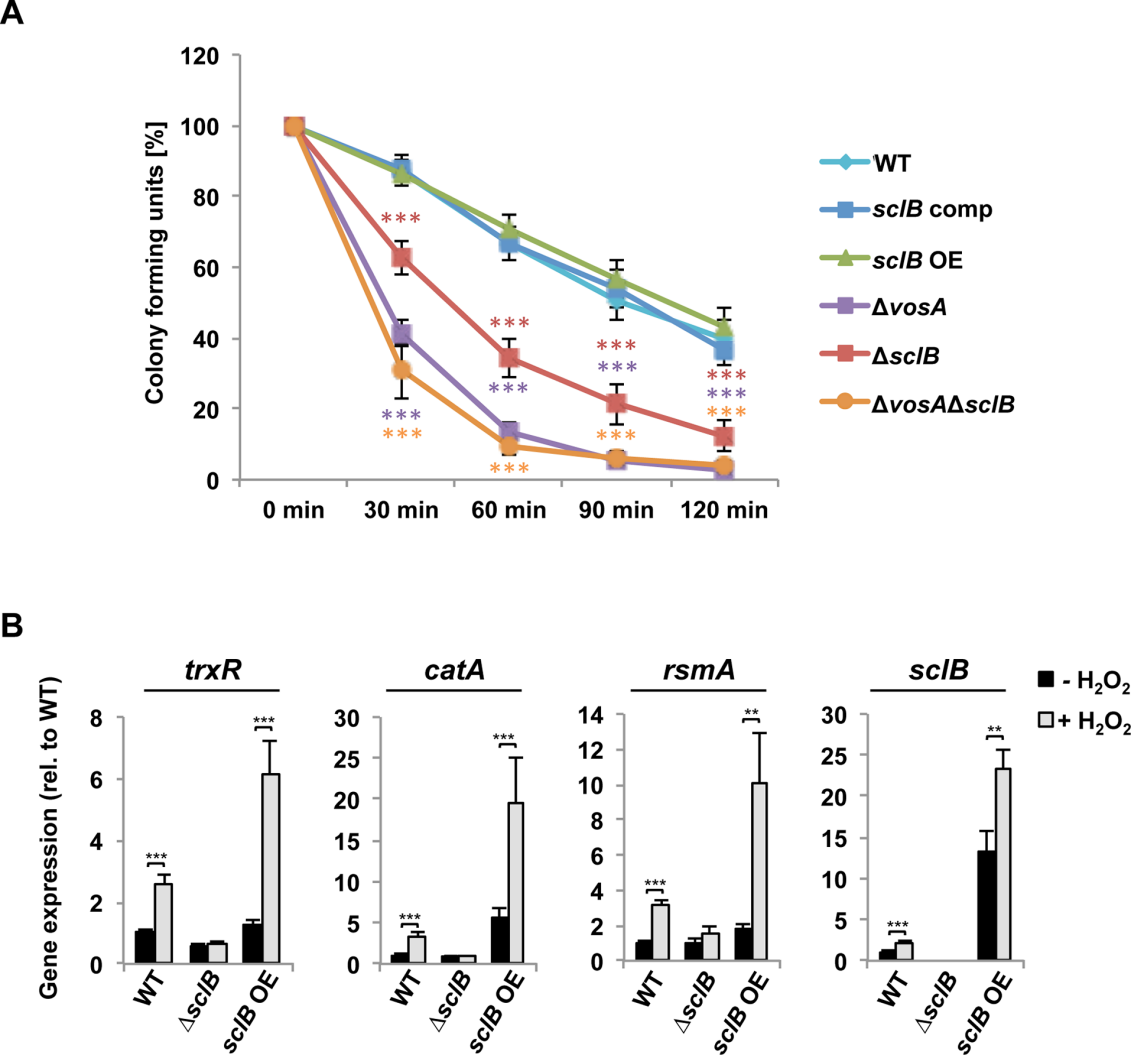
SclB regulates the oxidative stress response in *A*. *nidulans* in the presence of H_2_O_2_. A) Conidiospores of Δ*sclB*, Δ*vosA* and Δvos*A*Δ*sclB* strains show decreased survival in the presence of H_2_O_2_ compared to spores of wildtype (WT), *sclB* comp and *sclB* OE strains. Conidiospores were incubated with 100 mM H_2_O_2_ and approximately 200 spores plated at indicated time points. Error bars represent standard error of the mean of n ≥ 5 biological replicates (***P<0.001). B) qRT-PCR indicates that expression of *trxR*, *catA*, *rsmA*, as well as *sclB* itself is induced upon H_2_O_2_ stress in WT and *sclB* OE but not in Δ*sclB*. Strains were grown vegetatively for 24 h and subsequently liquid cultures were incubated for 30 min with (grey boxes) or without (black boxes) 5 mM H_2_O_2_ (**P<0.005, ***P<0.001).

The adaptive response to oxidative stress is required for fungal development as endogenous signal and is an important determinant for fungal fitness in corresponding environmental conditions [40,88]. SclB is involved in the regulation of spore viability (Fig 4C) and genome-wide transcriptional analyses show that several genes related to the response to oxidative stress are differentially expressed when *sclB* is absent (Fig 2 and S1 Table). Conidiospore survival was tested during H_2_O_2_ induced oxidative stress to analyze whether SclB is involved in the regulation of the oxidative stress response as well. Conidiospores of the wildtype, the complemented and the *sclB* OE strain show a linear loss in spore viability over time in the presence of 100 mM H_2_O_2_ (Fig 10A). In contrast, conidiospores of the Δ*sclB* strain show a more rapid loss in viability over time in the presence of 100 mM H_2_O_2_. Conidiospores from wildtype, *sclB* comp and *sclB* OE strains showed survival rates of approximately 86% after 30 min of H_2_O_2_ treatment, conidiospores of the Δ*sclB* strain showed only 62% survival. At the same time point conidiospores of the Δ*vosA* and the Δ*vosA*Δ*sclB* strains showed even further reduced viability of only 40% (Δ*vosA*) and 30% (Δ*vosA*Δ*sclB*), respectively. Similar differences were measured over the whole time period of examination. This suggests that SclB positively regulates the oxidative stress response in *A*. *nidulans*.

To investigate this further, expression of genes of the oxidative stress response was tested in submerged cultures in the presence or absence of H_2_O_2_. The glutathione and the thioredoxin system are important parts of the oxidative stress response [89–91]. The thioredoxin system is encoded by *trxA* (thioredoxin) and *trxR* (thioredoxin reductase) [90]. *trxA* was especially induced upon treatment with H_2_O_2_ in the *sclB* OE strain (S7 Fig). *trxR* is induced in wildtype in the presence of H_2_O_2_ but not induced in the Δ*sclB* strain (Fig 10B). It is also downregulated in the absence of *sclB* during unstressed growth (S1 Table). The *sclB* OE strain stressed with H_2_O_2_ shows an increased *trxR* upregulation compared to wildtype (Fig 10B). *glrA* encodes the glutathione reductase [92,93], which regulation was not dependent on the presence of *sclB* (S7 Fig). The *catA* gene, encoding the spore-specific catalase A, is upregulated in wildtype but not induced in Δ*sclB* in presence of H_2_O_2_ (Fig 10B). Expression of *catA* in the *sclB* OE strain is already upregulated during unstressed growth.

Several transcription factors are involved in the response to oxidative stress. *napA* encodes the most prominent oxidative stress regulator in *A*. *nidulans*. *napA* gene expression was not found to be significantly regulated under applied conditions (S7 Fig). RsmA is involved in the regulation of SMs and in oxidative stress response [91,94]. *rsmA* expression is around three fold induced in wildtype when H_2_O_2_ stress is applied (Fig 10B). In *sclB* OE the induction of *rsmA* expression in the presence of H_2_O_2_ is even higher (almost six fold), whereas *rsmA* expression is not induced by H_2_O_2_ in the Δ*sclB* strain. *sclB* itself is upregulated in wildtype and in *sclB* OE upon addition of H_2_O_2_ in comparison to unstressed situation (Fig 10B).

Taken together, these data suggest that SclB is involved in the regulation of the oxidative stress response in *A*. *nidulans* and specifically acts as a positive regulator of enzyme encoding genes, such as *catA* and thioredoxin genes, as well as the transcription factor-encoding gene *rsmA*.

### SclB is a nuclear localized protein and interacts with RcoA

C6 proteins are typical fungal transcription factors. *In silico* analyses predicted SclB to be localized in the nucleus as determined by CELLO [95] and WoLF PSORT [96]. SclB was fused N- and C-terminally to sGFP to examine subcellular localization *in vivo* (S8A Fig). The predicted molecular mass of both versions of the SclB GFP-fusion proteins is 87.46 kDa. Sizes of both fusion proteins determined by western hybridization are slightly higher than bioinformatically predicted (S8B Fig), indicating posttranslational modifications. Treatment of GFP-SclB crude extracts with Lambda phosphatase resulted in a band shift on a western blot, suggesting that SclB is phosphorylated during vegetative growth (S8C Fig). NetPhos 3.1 [97] predicted 28 codons for possible phosphorylation sites (score value between 0 and 1, cut off >0.7). LC-MS/MS analyses revealed three phosphorylated SclB residues S327, T464 and S506 in samples derived from vegetatively grown cultures, supporting that SclB is phosphorylated during vegetative filamentous growth (S9A Fig). However, mutation of these residues and two serines adjacent to S506 (S504 and S505) to alanine to mimic constant dephosphorylation (*sclB*^S327A,T464A,S506A^) or aspartic acid to mimic constant phosphorylation (*sclB*^S327D,T464D,S506D^) did not result in any obvious phenotype (S9B Fig) and the function of these phosphorylation sites therefore remains elusive.

**Fig 11:**
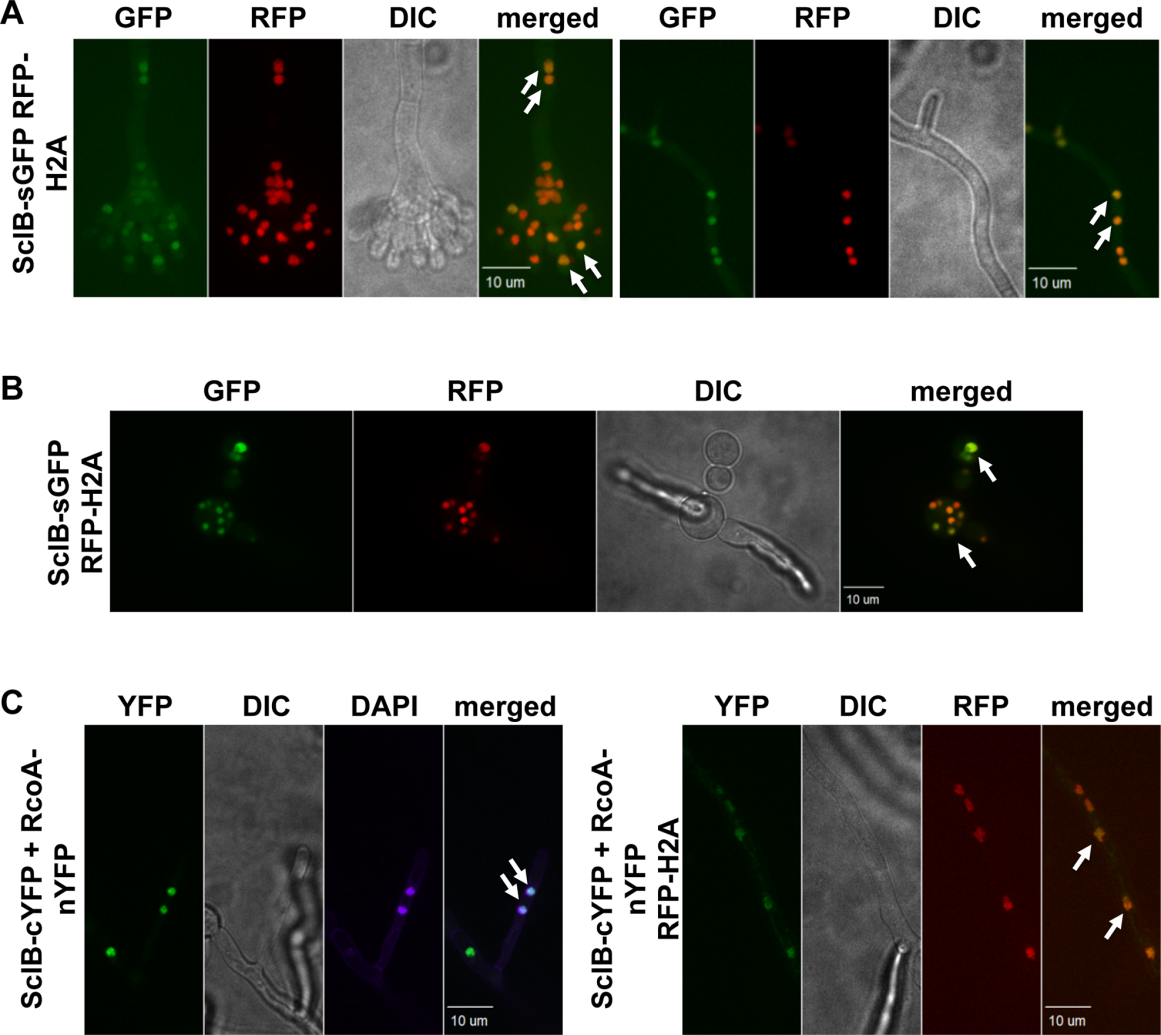
SclB is a nuclear protein. A) Fluorescence microscopic images of a strain expressing a SclB-GFP fusion protein under the native *sclB* promoter and mRFP-H2A to visualize nuclei. SclB-GFP fusion proteins are localized in nuclei of hyphae and conidiophores (white arrows). Strains were inoculated on solid MM and incubated for 24 h at 37°C in light. B) SclB-GFP fusion protein can be detected in nuclei (white arrows) of growing germlings. C) A strain expressing SclB and one half of a split YFP and RcoA, fused to the other half of the split YFP was constructed and grown for 36 h in liquid MM at 30°C. White arrows indicate fluorescence signals of the joint split YFP, what indicates a direct interaction of SclB and RcoA *in vivo*.

Both, the N- and C-terminal GFP fusion of SclB was expressed under control of the native *sclB* promoter and could complement the loss of *sclB*, demonstrating, that the fusion proteins are functional (S9A Fig). Fluorescence microscopy revealed a subcellular localization of both versions of the SclB fusion protein in nuclei of hyphae during all growth conditions tested (vegetatively, asexually and sexually grown) as well as in conidiospores (Fig 11A) and germlings (Fig 11B) indicating permanent nuclear localization of SclB.

GFP-trap pull downs with both, the N- and C-terminally tagged SclB versions, were conducted to investigate possible interactions of SclB with other proteins. These pull downs were conducted with cultures grown vegetatively, asexually and sexually and pulled down proteins were analyzed with LC-MS/MS. The majority of identified proteins are uncharacterized (S2 Table). Four importins were identified: the essential karyophorin KapF (importin) was identified solely in samples of vegetatively grown cultures, whereas KapJ was identified in samples from strains grown in submerged cultures, as well as in light. KapB and KapI were identified in samples grown in light or dark. Together with a predicted NES and a predicted NLS, this indicates specific control of nuclear localization for SclB.

RcoA was found in samples grown in submerged cultures and in the dark, conditions inducing sexual development in the wildtype. Furthermore, it was identified in samples grown in light, but below threshold. RcoA acts as transcriptional repressor and the RcoA-SsnF co-repressor-complex, which corresponds to yeast Tup1-Ssn6, is essential for growth in Aspergilli [98–101]. Bimolecular fluorescence complementation experiments (Bi-FC) were performed to verify direct interaction of SclB and RcoA *in vivo*. Strains were constructed for these experiments, which express fusion proteins, where one half of a split YFP (cYFP) was fused to SclB and the other half (nYFP) to RcoA [102]. Two additional strains, expressing either SclB-cYFP and free nYFP or RcoA-nYFP and free cYFP, served as controls (S9D Fig). Only a signal of the joint YFP halves, indicating a physical interaction of SclB and RcoA, could be identified in nuclei of hyphae (Fig 11C). This indicates that SclB can interact directly with RcoA *in vivo* and might execute some of its regulatory roles in developmental programs, secondary metabolism and oxidative stress response as a heterodimer.

## Discussion

The velvet domain protein VosA of *Aspergillus nidulans* binds more than a thousand fungal promoters and affects a substantial part of the transcriptome. One of these genes encodes the novel zinc cluster transcription factor SclB. VosA inhibits the expression of the *sclB* gene, which results in a slowdown and a decrease in asexual spore formation and a reduced production of secondary metabolites such as austinol, dehydroaustinol and emericellamides. SclB is not part of the fungal light response, which promotes the asexual program, but supports the cellular response upon H_2_O_2_ induced oxidative stress. SclB has a dual function as transcriptional activator for asexual development, but also as a repressor, presumably in combination with the repressor subunit RcoA, which we could identify as interacting partner. A genome-wide transcriptional analysis revealed that direct or indirect effects caused by the absence of the *sclB* gene result in more than 400 differentially expressed genes compared to wildtype (S1 Table). 1.5 times as many of these genes are downregulated, as upregulated, in the absence of *sclB*. A large group of these genes are related to metabolic processes, as carbon or sulphur metabolism, or transporter activity. This most likely is a consequence of the distorted development of the Δ*sclB* mutant. On the other hand, several secondary metabolite and developmental genes including asexual regulatory genes as *flbC* or *flbD*, and *rodA* or *dewA* required for asexual spore formation are differentially regulated when SclB is not present in the cell. This suggests that SclB regulates asexual development and interconnected secondary metabolism in *A*. *nidulans*. SclB is localized in nuclei of germlings, conidiophores and hyphae. Four karyophorins were identified as putative interaction partners of SclB under different growth conditions and suggest a complex nuclear entry or exit control. SclB is phosphorylated at at least three residues during vegetative growth, but the function of these posttranslational modifications is yet elusive.

**Fig 12:**
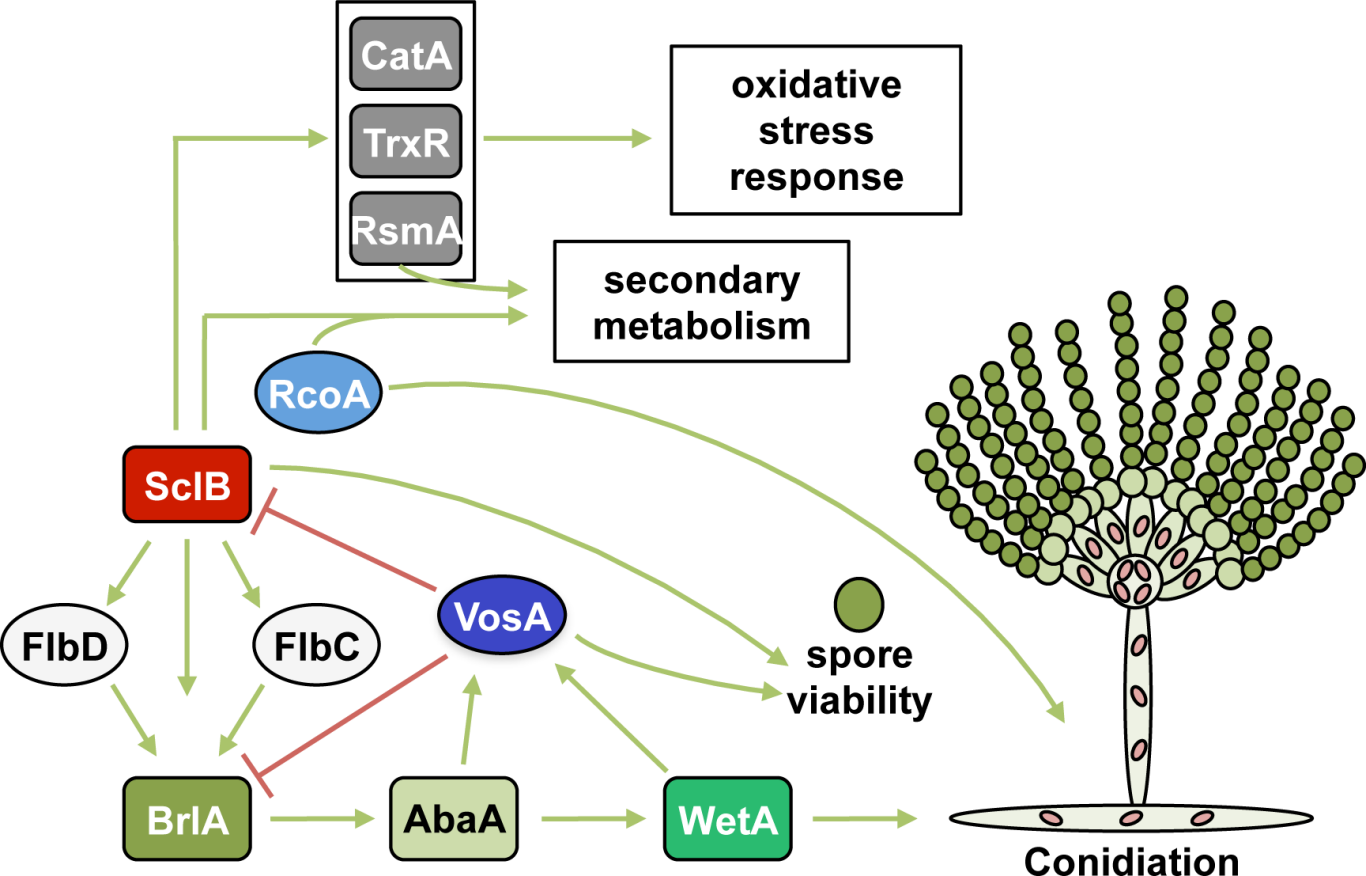
SclB is an activator of asexual development and secondary metabolites in *A*. *nidulans*. A model depicting the regulatory roles of SclB in *A*. *nidulans* is presented. SclB activates conidiation through regulation of *brlA*, *flbC* and *flbD*. SclB is repressed by VosA and both factors support conidiospore viability. SclB acts as activator of secondary metabolites, such as emericellamides, austinol and dehydroaustinol, and fulfills parts of its functions in conjunction with RcoA. SclB acts as regulator of the oxidative stress response and activates gene expression of *catA*, *trxR* and *rsmA*, thereby regulating the oxidative stress response and secondary metabolism. Green arrows indicate positive, red lines negative regulations.

Asexual spore formation requires the formation of the FluG protein. SclB accelerates an efficient formation of the asexual conidia in the absence of VosA by activating at least three regulatory genes downstream of FluG. Such an additional activator of conidiation had been predicted (Fig 7B) [11]. SclB increases *flbC* and *flbD* expression. The resulting FlbC and FlbD proteins as well as SclB activate the major asexual activator encoding gene *brlA*. The formation of the BrlA protein is necessary for the transition from stalk like aerial hyphae into mature conidiophores (Fig 12) [83].

The molecular control mechanism by which VosA inhibits asexual differentiation is complex. VosA does not only repress the formation of the *sclB* gene product that acts as activator of the conidiation cascade, but also represses *brlA* itself during vegetative growth. De-repression only takes place, when the fungus obtains developmental competence and is triggered within a time window by the appropriate external signals for conidia formation [4,8]. In the further course of ongoing asexual development, the *vosA* gene is activated by the BrlA-downstream factors AbaA and WetA. The VosA velvet domain protein represses again the *brlA* and *sclB* genes and fulfils together with the VelB velvet domain protein its function to support spore viability [4,8,26]. SclB supports spore viability as well. One possible explanation might be that *sclB* gene expression is repressed by the VosA-VosA homodimer, which also represses *brlA* expression, whereas spore viability might be a regulatory function of the VosA-VelB heterodimer.

SclB is not involved in the light control of *A*. *nidulans*, but is part of the response towards H_2_O_2_ induced oxidative stress. An internal oxidative stress signal caused by reactive oxygen species (ROS) serves as developmental signal in fungi and requires an appropriate fast and potent protective response [40,88,103]. ROS homeostasis therefore is crucial for the proceeding of asexual development. SclB activates elements of the fungal oxidative stress response including the thioredoxin system or *catA* for the spore specific catalase [89,90,104–106]. In addition, SclB activates the expression of the transcription factor RsmA during oxidative stress, which plays a similar dual role as SclB, because it is also part of the control of oxidative stress response and of secondary metabolism [91,94,107].

The SclB-mediated control for secondary metabolism includes several possible links to asexual differentiation. It is necessary for austinol, dehydroaustinol and emericellamide production and acts as activator of emericellamide, austinol and dehydroaustinol production through regulation of their gene clusters. An adduct of dehydroaustinol and diorcinol is able to overcome the conidiation defect of a Δ*fluG* mutant suggesting that they are involved in the FluG signal, which is crucial for the initiation of asexual development [108]. Orsellinic acid and the orsellinic acid-related diorcinol were also produced in high amounts in a Δ*csnE* mutant compared to wildtype [40]. CsnE is part of the conserved COP9 signalosome (CSN) which controls the specificity of ubiquitin E3 cullin RING ligases for the protein degradation in the 26S proteasome [109,110]. CSN is required for the link between sexual development and the appropriate secondary metabolism, light control and the protection against oxidative stress [111–113]. The SclB function is involved in the alternative differentiation program. SclB connects asexual development to its specific secondary metabolism and also acts at the interphase to the response to oxidative stress.

SclB interacts with RcoA *in vivo*. RcoA is a WD40 repeat protein, which regulates developmental programs and is required for the production of the mycotoxin sterigmatocystin as a member or the aflatoxin family [5,100,114,115]. A loss of *rcoA* in *A*. *nidulans* results in poor colony growth, impaired conidiation and the production of an orange pigment as indication of a misregulated secondary metabolism [100]. RcoA is part of the conserved SsnF-RcoA co-repressor complex corresponding to Ssn6-Tup1 in yeast, which represses numerous genes [99–101,116,117]. Target genes are repressed by several mechanisms such as through interacting with DNA-binding proteins and RNA polymerase II, through competition for promoter binding with other transcription factors, but also through histone acetylation and nucleosome positioning [118–122]. It is unclear whether there is only an RcoA-SclB heterodimer in the *A*. *nidulans* cell or whether SclB also interacts with RcoA-SsnF, because SsnF [99] could not be identified as putative SclB interaction partner. The exact molecular function of the SclB-RcoA interaction in the timely choreography of conidiation is unknown and might include as well activating as inhibiting control mechanisms during ongoing asexual development and its link to secondary metabolism and an oxidative stress response.

Zinc cluster DNA-binding proteins belong to the most abundant transcription factors in the fungal kingdom [62]. SclB is present in nearly all Aspergilli and especially its C6 DNA-binding domain is highly conserved. Most C6 proteins are involved in either i) primary or secondary metabolism or ii) developmental programs [67]. SclB rather acts as global regulator and interconnects asexual development, secondary metabolism and the response to oxidative stress. Its C6 domain exhibits an uncommon architecture that is only found in less than 6% of all C6 proteins in *A*. *nidulans*. Other characterized *A*. *nidulans* C6 proteins with the same architecture as SclB function specifically in primary metabolic programs (S7 Table) [65,66]. Scl-2 is the SclB counterpart of *A*. *niger*. Loss of the *sclB* ortholog in *A*. *niger* results in reduced conidiation and impaired secondary metabolism [53]. This indicates similar regulatory effects in conidiation and secondary metabolism of *A*. *niger* Scl-2 and *A*. *nidulans* SclB. Wildtype *A*. *niger* cells form sclerotia as resting structures under very defined conditions [53,123]. Scl-2 also acts as a sclerotia repressor, because a corresponding *scl-2* mutant strain produces sclerotia-like structures under conditions where the wildtype does not form these structures. SclB of *A*. *nidulans* is not a repressor of the formation of cleistothecia. Sclerotia have similarities with the sexual fruiting bodies of *A*. *nidulans* with the major difference that they are not linked to a sexual meiosis programme. These different control functions suggest that different fungi might have rewired the control of gene expression of this transcription factor in different developmental networks and contexts.

The proposed *sclB* ortholog of *A*. *fumigatus* (*Afu6g11110*) rescues the *A*. *nidulans* Δ*sclB* phenotype, which suggests that the molecular function of *sclB* therefore is conserved between *A*. *nidulans* and *A*. *fumigatus*. Some SclB functions might have changed in *A*. *fumigatus*, because it is dispensable for conidiation in this opportunistic human pathogen. Alternatively, a second redundant factor might compensate the effects of a *sclB* deletion, which is in agreement with other findings supporting that the conidiation cascade of *A*. *fumigatus* exhibits significant differences to its counterpart in *A*. *nidulans*. Deletion of *fluG* leading to diminished numbers of conidiophores in *A*. *nidulans* does not result in an obvious asexual phenotype in *A*. *fumigatus* [124,125] and functions of WetA, AbaA, velvet proteins or several Flb factors have changed [29,126].

Taken together, the VosA repression target SclB controls a novel genetic network in *A*. *nidulans*, which links conidiation to secondary metabolism and the response to oxidative stress. Further studies will broaden our understanding of the interconnection and complex mutual control of developmental programs and the production of bioactive molecules in response to environmental conditions and stresses in filamentous fungi. This is especially important, as a vast amount of bioactive natural products are still unknown and might have deleterious as well as beneficial potential to humans [38,127,128]. The SclB genetic network is a sub-network of the velvet domain network, which bridges secondary metabolism and development in fungi. In contrast, other known subnetworks of VosA, as BrlA regulating the conidiation cascade, are more specialized for a specific program. This study shows that velvet domain subnetworks include different categories as encaptic as BrlA, as well as independently acting elements as SclB. The amount of putative SclB targets and its congeneric as well as independent or even antithetic functions to VosA suggest that SclB, downstream of VosA, itself regulates a large network of downstream genes. VosA binds to more than thousand gene promoters and this network further extends through transcription factors as SclB that act themselves as master regulators.

## Material and methods

### Strains and growth conditions

AGB551 (*veA*+) was used as *A*. *nidulans* wildtype. Afs35 was used as *A*. *fumigatus* wildtype. Wildtype and mutant strains (see S3 Table) were grown in minimal medium (MM) (1% glucose, 7 mM KCl, 2 mM MgSO_4_, 70 mM NaNO_3_, 11.2 mM KH_2_PO_4_, 0.1% trace element solution pH 5.5 [129]) supplemented with 0.1% pyridoxine-HCl, 5 mM uridine, 5 mM uracil or 4-aminobenzoic acid, when needed. Strains were grown for two days on solid MM containing 2% agar in light at 37°C and two day old spores were harvested for further experiments. For synchronized growth strains were grown in submerged cultures for 24h and subsequently shifted onto solid agar plates. *Escherichia coli* strains (S4 Table) were grown on solid lysogeny broth (LB) [130] medium (1% tryptone, 0.5% yeast extract, 1% NaCl) or in liquid LB shaking on a rotary shaker at 37°C. 100 mg/ml ampicillin was added to prevent plasmid loss.

### Genomic DNA extraction

For extraction of genomic DNA strains were grown over night (o/n) in liquid cultures. Mycelia was harvested through Miracloth filters, frozen in liquid nitrogen and ground with a table mill. Ground mycelia was mixed with 500 μl genomic DNA lysis buffer [131] and incubated 15 min at 65°C. Subsequently mycelia solution was mixed with 100 μl 8 M potassium acetate and centrifuged for 15 min at 13,000 rpm at room temperature (RT). Supernatant was mixed with 100 μl 8 M potassium acetate and centrifuged for 15 min at 13,000 rpm at RT. Supernatant was mixed with 300μl isopropanol and centrifuged 10 min at 13000 rpm at RT. Pellets were washed twice with 70% ethanol and dried at 42°C before resolving in H_2_O at 65°C.

### Plasmid construction and preparation

DNA fragments for plasmid constructions were amplified with PCR from *A*. *nidulans* FGSC A4 or *A*. *fumigatus* Afs35 genomic DNA, respectively, and cloned into pBluescript SK(+) using the Geneart Seamless Cloning and Assembly kit, the Seamless PLUS Cloning and Assembly Kit and the Seamless Cloning and Assembly Enzyme Mix (Invitrogen) or via fusion PCR and subsequent cloning into pBluescript SK(+) with the CloneJET PCR Cloning Kit (Thermo Scientific) or via employment of T4 ligase (Thermo Scientific) according to manufacturer’s instructions.

Plasmids were amplified in *E*. *coli* and extracted with the Qiaprep Spin Miniprep Kit (Qiagen) or the NucleoSpin Plasmid Miniprep Kit (Macherey-Nagel) according to manufacturer’s instructions.

For the production of the plasmids pME4304 and pME4305 the pyrithiamine resistance cassette (ptrA) of pSK485 [72] was replaced by the nourseothricin resistance cassette (natR) from plasmid pNV1 [132] (primer pair JG846/847) or the phleomycin resistance cassette (phleoR) from plasmid pME3281 [133] (primer pair JG848/849), respectively, by usage of the Seamless Cloning and Assembly Kit (Invitrogen). Both cassettes additionally carried one half of the *Pme*I restriction site at both ends. The recyclable marker cassettes from pME4304 and pME4305 are called natRM and phleoRM, respectively, in the following. The recyclable marker cassette from pSK485 is called ptrARM in the following.

### Construction of plasmid pME4575 and Δ*sclB* strain in *A*. *nidulans*

For production of pME4575, the 2.7 kb long 5’ and 2.2 kb long 3’ region of the *sclB* (*AN0585*) gene were amplified with primer pairs kt208B/214 and kt211/224, respectively, and together with the natRM cassette cloned into the *Eco*RV multiple cloning site of pBluescript SK(+), employing the Seamless Cloning and Assembly Kit (Invitrogen). The deletion cassette was subsequently excised with *Mss*I and transformed into AGB551, resulting in the strain AGB1007.

### Construction of plasmid pME4578 and *sclB* OE strain in *A*. *nidulans*

For production of pME4578, the 1.3 kb nitrate-inducible promoter (^P^*niaD*), amplified with primer pair kt251/252, the *sclB* open reading frame (ORF) itself and a small part of the 3’ region (1.8 kb), amplified with kt241/253, the *sclB* 5’ region (kt208b/214) and the natRM cassette were cloned into pBluescript SK(+), employing the Seamless Cloning and Assembly Kit (Invitrogen). The ^P^*niaD*::*sclB* construct was subsequently excised with *Mss*I and transformed into AGB551, resulting in AGB1008.

### Construction of plasmids pME4576 and 4579 and GFP-fusion strains of SclB in *A*. *nidulans*

For production of pME4576, *sgfp* was amplified from pME4292 with primers kt229/SR18 and, together with the *sclB* ORF and its 5’ flanking region (4.4 kb, primers kt208b/228), the *sclB* 3’ region (primers kt211/224) and the natRM cassette was cloned into pBluescript SK(+), employing the Seamless Cloning and Assembly Kit (Invitrogen). Subsequently, the *sclB*::*sgfp* construct was excised from pME4576 with *Mss*I and transformed into AGB1007 resulting in AGB1009. Successful transformation at the correct locus was verified by Southern hybridization.

For production of pME4579, the 5’ flanking region of *sclB* (primers kt209/307), *sgfp* (primers SR120/121), *sclB* ORF (primers kt230/231), the phleoRM cassette and the *sclB* 3’ flanking region (primers kt211/225) were cloned into pBluescript SK(+), employing the Seamless Cloning and Assembly Kit (Invitrogen). Subsequently, the *sgfp*::*sclB* construct was excised from pME4579 with *Mss*I and transformed into AGB1007, obtaining AGB1010.

The plasmid pME3173 was transformed into AGB1009 and AGB1010, resulting in AGB1012 and AGB1013, respectively, to facilitate the visualization of nuclei. pME3173 was transformed into AGB551 resulting in AGB1014 to obtain a suitable negative control for microscopy.

### Contruction of plasmid pME4577 and the *sclB* complementation strain in *A*. *nidulans*

For production of pME4577, the *sclB* ORF and its 5’ UTR (4.4 kb, primers kt208b/231), the *sclB* 3’ UTR (primers kt211/224) and the phleoRM cassette were cloned into pBluescript SK(+), employing the Seamless Cloning and Assembly Kit (Invitrogen). The *sclB* complementation cassette was excised from pME4577 with *Mss*I and cloned into AGB1007, resulting in AGB1011.

### Construction of plasmids pME4581 and pME4582, and strains: Δ*fluG* and the *fluG*/*sclB* double mutants in *A*. *nidulans*

For production of pME4581, 1 kb of the *fluG* 5’ flanking region (primers kt341/342), 1 kb of the 3’ flanking region (primers kt343/364) and the phleoRM cassette were cloned into the *EcoR*V restriction site of pBluescript SK(+), employing the Seamless Cloning and Assembly Kit (Invitrogen). The *fluG* deletion cassette was excised from pME4581 with *Mss*I and integrated into AGB551, AGB1007 and AGB1008, resulting in AGB1016, AGB1017 and AGB1018, respectively.

### Construction of plasmid pME4589 and strains: Δ*brlA* and the *brlA*/*sclB* double mutants in *A*. *nidulans*

For production of pME4589, 1.7 kb of the *brlA* 5’ region (primers kt487/488), 1.2 kb of the *brlA* 3’ region (primers kt489/490) and the phleoRM cassette were cloned into pBluescript SK(+), employing the Seamless Cloning and Assembly Kit (Invitrogen). The Δ*brlA* cassette was excised from pME4589 with *Mss*I and integrated into AGB551 and AGB1007, resulting in AGB1031 and AGB1032, respectively.

### Construction of plasmid pME4591 and strains: Δ*flbB* and the *flbB*/*sclB* double mutants in *A*. *nidulans*

For production of pME4591, 1.2 kb of the *flbB* 5’ region (primers kt515/516), 1 kb of the *flbB* 3’ (primers kt517/518) and the phleoRM cassette were cloned into pBluescript SK(+), employing the Seamless Cloning and Assembly Kit (Invitrogen). The Δ*flbB* cassette was excised from the pME4591 with *Mss*I and integrated into AGB551, AGB1007 and AGB1008, resulting in AGB1035, AGB1036 and AGB1037, respectively.

### Construction of plasmid pME4593 and strains: Δ*flbC* and the *flbC*/*sclB* double mutants in *A*. *nidulans*

For production of pME4593, 1.2 kb of the *flbC* 5’ region (primers kt519/520), 1 kb of the *flbC* 3’ region (primers kt521/522) and the phleoRM cassette were cloned into pBluescript SK(+), employing the Seamless Cloning and Assembly Kit (Invitrogen). The Δ*flbC* cassette was excised from pME4593 with *Mss*I and integrated into AGB551, AGB1007 and AGB1008, resulting in AGB1039, AGB1040 and AGB1041.

### Construction of plasmid pME4595 and strains: Δ*flbD* and the *flbD*/*sclB* double mutants in *A*. *nidulans*

For production of pME4595, 1.1 kb of the *flbD* 5’ region (primers kt523/524), 1.2 kb of the *flbD* 3’ region (primers kt525/526) and the phleoRM cassette were cloned into pBluescript SK(+), employing the Seamless Cloning and Assembly Kit (Invitrogen). The Δ*flbD* cassette was excised from pME4595 with *Mss*I and integrated into AGB551, AGB1007 and AGB1008, resulting in AGB1043, AGB1044 and AGB1045, respectively.

### Construction of plasmid pME4597 and strains: Δ*flbE* and the *flbE*/*sclB* double mutants in *A*. *nidulans*

For production of pME4597, 1.3 kb of the *flbE* 5’ region (primers kt527/528), 1.1 kb of the respective 3’ region (primers kt529/530) and the phleoRM cassette were cloned into pBluescript SK(+), employing the Seamless Cloning and Assembly Kit (Invitrogen). The Δ*flbE* cassette was excised from pME4597 with *Mss*I and integrated into AGB551, AGB1007 and AGB1008, resulting in AGB1047, AGB1048 and AGB1049.

### Constructions of plasmids pME4599, pME4600 and pME4601, and and Bi-FC strain construction for interaction studies of SclB with RcoA in *A*. *nidulans*

For Bi-FC plasmid construction, *sclB* and *rcoA* were amplified from cDNA instead of genomic DNA. The bidirectional nitrate-inducible promoter was excised from pME4607 in a two-step digestion with *Mss*I and *Smi*I and both, the pME4607 backbone vector and the nitrate inducible promoter were utilized for all Bi-FC constructs.

For production of pME4599, the *sclB* (primers kt407/415) and *rcoA* ORFs (primers kt409/418) were fused to *ceyfp* (primers kt416/417) and *neyfp* (primers kt421/422), respectively by fusion PCR [134]. Subsequently, *sclB*::*ceyfp*, *rcoA*::*neyfp* and the bidirectional nitrate-inducible promoter were cloned into the pME4607 backbone vector, employing the Seamless Cloning and Assembly Kit (Invitrogen). pME4599 was ectopically integrated into AGB1007 resulting in AGB1051 and AGB1014, resulting in AGB1052.

For production of pME4601, free *ceyfp* (primers kt416/SR195), *rcoA*::*neyfp* and the bidirectional nitrate-inducible promoter were cloned into the pME4607 backbone vector, employing the Seamless Cloning and Assembly Kit (Invitrogen). pME4601 was introduced into AGB551 and AGB1014, resulting in AGB1054 and AGB1056, respectively. For production of pME4600, free *neyfp* (primers kt422/SR193), *sclB*::*ceyfp* and the nitrate-inducible promoter were cloned into the pME4607 backbone vector, employing the Seamless Cloning and Assembly Kit (Invitrogen). pME4600 was introduced into AGB551 and AGB1014, resulting in AGB1053 and AGB1055, respectively.

### Construction of plasmids pME4574 and strains for Δ*veA* and *veA*/*sclB* double mutant strains of *A*. *nidulans*

For production of pME4574, the *veA* 5’ (primers JG863/985) and 3’ (primers JG865/866) regions and the natRM cassette were cloned into pBluescript SK(+), employing the Seamless Cloning and Assembly Kit (Invitrogen). The Δ*veA* construct was excised from pME4574 with *Mss*I and transformed into AGB551 resulting in AGB1066. The Δ*sclB* cassette from pME4575 was integrated into AGB1066, resulting in AGB1067.

### Construction of plasmids pME4605 and strains for Δ*velB* and *velB*/*sclB* double mutant strains of *A*. *nidulans*

For production of pME4605, the *velB* 5’ (primers SR05/06) and 3’ (primers SR07/08) regions and the natRM cassette were cloned into pBluescript SK(+), employing the Seamless Cloning and Assembly Kit (Invitrogen). The Δ*velB* construct was excised from pME4605 with *Mss*I and transformed into AGB551 resulting in AGB1064. The Δ*sclB* cassette from pME4575 was integrated into AGB1064, resulting in AGB1065.

### Construction of plasmids pME4602 and strains for Δ*velC* and *velC*/*sclB* double mutant strains of *A*. *nidulans*

For production of pME4602, the *velC* 5’ (primers kt203/145) and 3’ (primers kt146/204) regions and the natRM cassette were cloned into pBluescript SK(+), employing the Seamless Cloning and Assembly Kit (Invitrogen). The Δ*velC* construct was excised from pME4602 with *Mss*I and transformed into AGB551 resulting in AGB1062. The Δ*sclB* cassette from pME4575 was integrated into AGB1062, resulting in AGB1063.

### Construction of plasmids pME4603 and strains for Δ*vosA*, the *vosA*/*sclB* double mutant and *sclB* OE in Δ*vosA* strains of *A*. *nidulans*

For production of pME4603, the *vosA* 5’ (primers SR11/12) and 3’ (primers SR13/14) regions and the natRM cassette were cloned into pBluescript SK(+), employing the Seamless Cloning and Assembly Kit (Invitrogen). The Δ*vosA* construct was excised from pME4603 with *Mss*I and transformed into AGB551 and AGB1007, resulting in AGB1057 and AGB1058, respectively. pME4578 was integrated into AGB1057, resulting in AGB1059.

### Construction of plasmids pME4606 and the Δ*sclB* strain in *A*. *fumigatus*

For production of pME4606, the *sclB* 5’ (primers kt215/221) and 3’ (primers kt218/226) flanking regions and the ptrARM were cloned into pBluescript SK(+), employing the Seamless Cloning and Assembly Kit (Invitrogen). The Δ*sclB* cassette was excised from pME4606 with *Mss*I and integrated into Afs35, resulting in AfGB129.

### Construction of plasmid pME4580 and pME4610 and strain *sclB*^S327A,T464A,S504-506A^ and *sclB*^S327D,T464D,S504-506D^ in *A*. *nidulans*

For production of pME4580, the *sclB* 5’ region and the *sclB* ORF, the *sclB* 3’region and the phleoRM marker cassette were cloned into pBluescript SK(+), employing the Seamless Cloning and Assembly Kit (Invitrogen): the first 1 kb part of the *sclB* ORF together with its 1.9 kb 5’ region was amplified with primers kt209/430, introducing the first mutation in the gene product (S327A). The next 431 bp of the *sclB* ORF were amplified with primers kt431/432, introducing the mutation T464A in the gene product. Adjacent 135 bp were amplified with the primer pair kt433/434 and the last 172 bp of the *sclB* ORF were amplified with the primer pair 442/231, introducing S504-506A in the gene product. The mutated *sclB* ORF and its 5’ adjacent region were fused in a series of fusion PCRs [134] from these four sequences. The complete mutated *sclB* ORF and its 5’ region, the *sclB* 3’ adjacent region (primers kt211/225) and the phleoRM cassette were cloned into pBluescript SK(+) in a seamless cloning reaction. The *sclB*^S327A,T464A,S504-506A^ cassette was excised from pME4580 with *Mss*I and integrated into AGB1007, resulting in AGB1015.

Similarly, the sc*lB*^S327D,T464D,S504-506D^ plasmid pME4610 was constructed using primers kt209/651, kt652/653, kt654/655 and kt657/696. The *sclB*^S327D,T464D,S504-506D^ cassette was excised from pME4610 with *Mss*I and integrated into AGB1007, resulting in AGB1147.

### Construction of plasmid pME4587 and strains: Δ*abaA* and the *abaA*/*sclB* double mutants in *A*. *nidulans*

For production of pME4587, 1.5 kb of the *abaA* 5’ region (primers kt354/355), the phleoRM cassette and 1.4 kb of the *abaA* 3’ region (primers kt356/363) were cloned into pBluescript SK(+), employing the Seamless Cloning and Assembly Kit (Invitrogen). The Δ*abaA* cassette was excised from pME4587 with *Mss*I and integrated into AGB551 and AGB1007, resulting in AGB1028 and AGB1029, respectively.

### Construction of plasmid pME4609 and a strain expressing *AfusclB* in *A*. *nidulans* Δ*sclB* background

For production of pME4609, the *sclB* 5’ (primer pair kt209/603) and 3’ regions were amplified from *A*. *nidulans* genomic DNA. The *sclB* ORF was amplified with primer pair kt254/233 from *A*. *fumigatus* genomic DNA and the three fragments were together with the natRM cassette cloned into pBluescript SK(+). The construct was excised from pME4609 using *Mss*I and transformed into AGB1007, resulting in AGB1042.

### Transformation

*A*. *nidulans* was transformed by polyethylene glycol-mediated protoplast fusion as described before [135,136]. *E*. *coli* transformations were carried out as described in [137,138]. Plasmids used in this study are given in S5 Table and oligonucleotides can be found in S6 Table. Successful transformation of constructs into *A*. *nidulans* was verified by Southern hybridization [139] employing the AlkPhos Direct Labelling and Detection System according to manufacturer’s instructions (GE Healthcare).

### Spore viability assay and spore survival assay

Conidiospores were harvested in 0.96% NaCl solution containing 0.002% Tween 80 after 2 days and counted with a hemocytometer (Marienfeld Superior). Conidiospores were diluted with 0.96% NaCl solution containing 0.002% Tween 80, and kept at 4°C. Aliquots of 200 spores of these dilutions were plated after zero and seven days and plates were incubated for two days at 37°C in the light. This test was performed in triplicates per experimental day.

For spore survival in the presence of 100 mM H_2_O_2_, spores were diluted with 0.96% NaCl solution containing 0.002% Tween 80 in 15 ml reaction tubes and 100 mM H_2_O_2_ was added. Reaction tubes were kept in the dark at RT under constant gyration to prevent sedimentation of spores. 200 spores were plated at indicated time points and plates were incubated as mentioned above.

Statistical analyses were conducted with t-tests using standard deviations of wildtype data against indicated mutant data sets.

### Secondary metabolite extraction

For extraction of secondary metabolites from asexually grown cultures 1*10^6^ spores were plated and grown for 3 or 7 days in light. Spores were completely washed off and the agar was cut into small pieces. Subsequently, secondary metabolites were extracted from agar pieces with 300 ml ethyl acetate by shaking at 160 rpm at 30°C for 30 min followed by 15 min ultra-sonication at highest level. Ethyl acetate was evaporated and the crude extract was kept at -20°C. For extraction from vegetatively grown cultures, 1*10^7^ spores were grown in submerged cultures for 48 h at 37°C on a rotary shaker and mycelia were removed with Miracloth filters. Extraction procedure was followed according to Gerke and co-workers [38]. Samples were stored at -20°C.

### Secondary metabolite analysis by high-performance liquid chromatography (HPLC) coupled with a UV/VIS diode array detector (UV/VIS-DAD) and an evaporative light scattering detector (ELSD)

Analytical HPLC/UV-DAD/ELSD measurements were performed using the following system: HPLC pump 420, SA 360 autosampler, Celeno UV-DAD HPLC detector, ELSD-Sedex 85 evaporative light-scattering detector (ERC)) with a Nucleodur 100–5 C18 end-capped (ec) column (250 mm x 3 mm) and the solvent system: A = H_2_O + 0.1% (v/v) trifluoroacetic acid (TFA), B = acetonitrile + 0.1% (v/v) TFA (Goebel Instrumentelle Analytik GmbH). Secondary metabolite extracts were dissolved in 500 μl methanol and an injection volume of 20 μl was analyzed under gradient conditions (20% B to 100% B in 20 minutes) with a flow rate of 0.5 ml/min.

HPLC data was analyzed with the Geminyx III software (Goebel Instrumentelle Analytik GmbH).

### UHPLC-UV and UHPLC-ESI-HRMS/MS analysis of secondary metabolites

For UHPLC-UV and UHPLC-ESI-HRMS/MS analysis crude extracts were solved in 1 ml methanol and analyzed using a Dionex Ultimate 3000 system (Thermo Scientific) connected to an Impact II qTof mass spectrometer (Bruker). 5 μl of each sample was injected for separation on an UHPLC reversed phase column (Acquity UPLC BEH C18 1.7 lmRP 2.1 x50 mm column (Waters) with an Acquity UPLC BEH C18 1.7 lmRP 2.1 x 5 mm pre-column (Waters)) applying a linear acetonitril/0.1% formic acid in H_2_O/0.1% formic acid gradient (from 20% to 95% acetonitril/0.1 formic acid in 20 min) with a flow rate of 0.4 ml/min at 40°C. For internal mass calibration a 10 mM sodium formate solution was used. Data analysis and sum formula predictions were performed with Bruker Compass DataAnalysis 4.3.

### Expression and purification of GST tagged VosA

GST tagged VosA protein was expressed and purified, as described by Ahmed and collaborators [2]. Purification was executed and monitored on an Äkta Explorer10 system (GE Healthcare). Amicon Ultra Centrifugal Filter Units (Millipore) were used for concentration after size exclusion chromatography.

### EMSA

EMSAs were performed as described earlier [2]. Briefly DNA probes were generated by annealing a reverse-complementary oligonucleotide pair. Protein and DNA was mixed and incubated 15 min at RT and dispersed according to molecular weight on a 6% polyacrylamide gel in 0.5% running buffer prior to staining with ethidium bromide.

### Microscopy

Photomicrographs were obtained with an Axiolab microscope (Carl Zeiss Microscopy) and a SZX12-ILLB2-200 binocular microscope (Olympus). Fluorescence microscopy was performed with a Zeiss AxioObserver Z.1 inverted confocal microscope, equipped with Plan-Neofluar 63x/0.75 (air) and Plan-Apochromat 100x/1.4 oil objectives (Zeiss). The SlideBook 6.0 software (Intelligent Imaging Innovations) was used for picture processing.

Strains were grown in 8-well borosilicate cover glass system (Thermo Scientific) in 400 μl MM supplemented as mentioned above, when needed, or on glass slides covered with 1 ml solid MM supplemented as mentioned above, when needed, at 37°C or 30°C. GFP-signals were normalized against wildtype background signal to subtract fungal auto fluorescence. Nuclei were visualized by ectopic integration of ^*p*^*gpdA*::*rfp*::*h2A* into the respective strains or through staining with 0.1% 4’,6’-diamidino-2phenylindole (DAPI).

### Conidiospore and cleistothecia quantification

Conidiospore numbers were determined with a Coulter Z2 particle counter (BECKMAN COULTER GMBH, Krefeld, Germany) or with a Thoma cell counting chamber (hemocytometer) (Marienfeld Superior). For quantifying cleistothecia, agar plugs of 5 mm^2^ were cut out from plated using the larger side of a 200 μl pipette tip and cleistothecia were individualized on a fresh agar plate and counted with help of a binocular microscope SZX12-ILLB2-200 binocular microscope (Olympus).

ANOVA and t-test statistical analyses were conducted using standard deviations. Mutant samples were always compared to wildtype data for two-sample comparison through t-test.

### RNA isolation and cDNA synthesis for quantitative real-time-PCR

For RNA isolation strains were grown vegetatively or asexually. Mycelia was harvested through sterile filters (Miracloth) and immediately frozen in liquid nitrogen. Frozen mycelia were ground with a table mill (Retsch) directly before RNA extraction. RNA was extracted with the RNeasy Plant Miniprep Kit (Qiagen) according to manufacturer’s instructions. cDNA was transcribed from 0.8 μg RNA with the QuantiTect Reverse Transcription Kit (Qiagen).

### Quantitative real-time-PCR

To measure gene expression real-Time-PCR was performed by using MESA GREEN qPCR MasterMix Plus for SYBR Assay (Eurogentec) in a CFX Connect Real-Time System (BioRad) and analysed with the CFX Manager software (BioRad). Expression of the housekeeping genes *gpdA* (*A*. *nidulans* and *A*. *fumigatus*), *h2A* (*A*. *nidulans* and *A*. *fumigatus*) and *15S rRNA* (*A*. *nidulans*) were used for normalization.

For measurement of the expression of oxidative-stress related genes, strains were grown in submerged cultures at 37°C on a rotary shaker for 24 h. Subsequently, 5 mM H_2_O_2_ was added. Control strains were left untreated. Incubation was prolonged for another 30 min shaking on the rotary shaker and mycelia were harvested as described above.

### Genome-wide transcriptional analysis

Total RNA of strains grown under submerged culture conditions for 24 h at 37°C under constant agitation on a rotary shaker was isolated using the Direct-zol Miniprep Kit (Zymo Research) according to manufacturer’s conditions. RNA quality control was performed on a Bioanalyzer 2100 Fragment Analyzer using a Pico Chip (RNA) (Agilent).

RNA sequencing was performed at the Core Unit, the Transcriptome and Genome Analysis Laboratory, University Medical Center Göttingen. RNA integrity was assessed using the Fragment Analyzer (Advanced Analytical) and only samples exhibiting RNA integrity number (RIN) > 8 were selected for sequencing. Libraries were performed starting with 800 ng of total RNA using the TruSeq Stranded Total RNA Sample Prep Kit from Illumina (Cat. No. RS-122-2201). Library sizing (295–320 bp) and quality was performed using the Fragment Analyzer (Advanced Analytical). Library quantitation was performed by using Promega’s QuantiFluor dsDNA System. RNA-sequencing was performed using the Illumina HighSeq-4000 platform (SR 50 bp; >30 Mio reads /sample). Demultiplexig was done using bcl2fastq2.

Raw reads were aligned using STAR version STAR_2.4.1a [140] against EnsemblFungi [141] revision 37 *Aspergillus nidulans* genome. Differential expression analysis was performed using edgeR [142].

Information gathered from the Aspergillus Genome Database (AspGD) [64] and Fungal and Oomycete Genomic Resources Database (FungiDB) [73] were used to categorize genes according to putative functions of their products. AspGD and FungiDB were employed for updated respective descriptions. For genetic ORFs, which were merged into a new ORF in FungiDB (FungiDB 36; released 19. Feb. 2018), the new merged ORF was taken into consideration for all downstream analyses.

Genome wide transcriptome data was submitted to EBI ArrayExpress under accession E-MTAB-6996.

### Protein isolation

Strains were grown under vegetative conditions and mycelia were harvested through sterile filter (Miracloth) and directly frozen in liquid nitrogen. Frozen mycelia were ground in liquid nitrogen with a table mill and approximately 200 mg was mixed with 300 μl B^+^ buffer (300 mM NaCl, 100 mM Tris pH 7.5, 10% glycerol, 1 mM EDTA, 0.1% NP-40) supplemented with 1.5 mM DTT, complete EDTA-free protease inhibitor cocktail (ROCHE), 0.001 mM PMSF, phosphatase inhibitor mix (1 mM NaF, 0.5 mM sodium-orthovanadate, 8 mM ß-glycerolphosphate disodium pentahydrate) and 1.5 mM benzamidine, and centrifuged for 15 min at 13000 rpm at 4°C. Supernatant was transferred into fresh test tubes and protein concentration was measured with a NanoDrop ND-1000 spectrophotometer.

### GFP-Trap

Protein pulldowns employing GFP-trap_A beads (Chromotek) were conducted as described earlier [98,143] with some alterations. *A*. *nidulans* strains were inoculated in a concentration of 5*10^8^ spores in 500 ml MM. Mycelia were harvested and immediately frozen in liquid nitrogen. Frozen mycelia were ground with a table mill in liquid nitrogen. Ground mycelia were mixed with B^+^ buffer in a ratio of 1:1 and centrifuged twice for 20 min at 4000 rpm at 4°C. Supernatant was filtered through 20 μm sterile filters (Sartorius) and mixed with 1:100 GFP-trap_A beads (Chromotek) and incubated o/n at 4°C.

### Western hybridization analyses

Equal amounts of protein were loaded on 10% SDS gels (separation gel: 2.8 ml H_2_O, 3.75 ml 1 M Tris pH 8.8, 100 μl 10% (w/v) SDS, 3.3 ml 30% (v/v) acrylamide, 10 μl TEMED, 50 μl 10% (w/v) APS; stacking gel: 3.67 ml H_2_O, 625 μl 1 M Tris pH 6.8, 30 μl 10% (w/v) SDS, 650 μl 30% (v/v) acrylamide, 5 μl TEMED, 25 μl 10% (w/v) APS) and separated at 200V. Proteins from SDS gels were blotted for 1h at 100 V ice cooled or at 35 V o/n at RT to nitrocellulose membranes (Whatman). Membranes were blotted with 5% skim milk powder dissolved in TBST buffer (10 mM Tris-HCl pH8.0, 150 mM NaCl, 0.05% Tween 20) for 1 h at RT and subsequently probed with 1:250 diluted GFP antibody (sc-9996, Santa Cruz Biotechnology). Following, membranes were washed three times in TBST and horseradish peroxidase coupled mouse antibody (115-035-003, Jackson Immuno Research) was applied as secondary antibody in a dilution of 1:2000.

### Dephosphorylation assay

Crude cell extracts were prepared as described above. B^+^ buffer was not supplemented with phosphatase inhibitor mix for this experiment. Crude cell extract were mixed with or without lambda phosphatase (NEB) according to manufacturer’s conditions and with or without phosphatase inhibitor mix in excess, and incubated for 30 min. at 30°C prior to boiling for 10 min. at 95°C together with loading dye. Subsequently, western hybridization experiments were performed as described above.

### Protein digestion with trypsin and protein identification with LC-MS/MS

Trypsin digestion of proteins was performed as published by Shevchenko and collaborators using Sequencing Grade Modified Trypsin (Promega) [144]. Following this procedure peptides were purified using the StageTip purification method [145,146]. Purified peptides were separated by reversed-phase liquid chromatography employing an RSLCnano Ultimate 3000 system (Thermo Scientific) followed by mass analysis with an Orbitrap Velos ProHybrid mass spectrometer (Thermo Scientific) as described [98,143,147,148]. For further details see [149].

MS/MS2 data processing for peptide analysis and protein identification was performed either with the MaxQuant 1.5.1.0 and Perseus 1.5.3 or the Proteome Discoverer 1.4 software (Thermo Scientific) and the Mascot and SequestHT search algorithms. Phosphosite probabilities were calculated with the phosphoRS search algorithm [150,151].

Three unique peptides [152] and three MS/MS counts were demanded for positive protein identification. Furthermore, only proteins identified from at least two out of three biological repetitions were considered further. Proteins also identified from the control strain (AGB596) were regarded as false-positives and excluded from further consideration.

## Supporting information

S1 Fig SclB of *A. nidulans* has orthologs in Aspergilli and other fungal groups. The C6 domain of SclB from *A*. *nidulans* was used for an *in silico* BLAST database search and C6 domains were aligned [59] for orthologs among the fungal kingdom. Phylogenetic analyses were conducted using a set of phylogeny programs comprising MUSCLE, Gblocks, PhyML and TreeDyn [57,58].
(TIF)

S2 Fig SclB is conserved between *A. nidulans* and *A. fumigatus*. A) Loss of *sclB* does not result in an obvious conidiation phenotype in *A*. *fumigatus* (left side). Integration of the *sclB* ORF of *A*. *fumigatus* into an *A*. *nidulans* Δ*sclB* strain results in complementation of the wildtype phenotype (Δ*sclB*::*AfusclB*, right side). Strains were grown on solid MM for 3 days at 37°C. B) Schematic depiction of the *sclB* ORF from *A*. *nidulans* and *A*. *fumigatus* and their respective gene products. Grey boxes represent introns, bp = base pairs, Zn = C6 domain, NLS = nuclear localization sequence, NES = nuclear export signal, aa = amino acids. An alignment of the C6 domains (highlighted in orange) and adjacent residues of both proteins is shown in the middle. Asterisks indicate the six conserved cysteine residues of the C6 domain. The C6 domain (highlighted in orange) is highly conserved between both fungi with only two exceptions (in grey).
(TIF)

S3 Fig SclB is a positive regulator of conidiation. A) qRT-PCR shows no differences in gene expression of *sfgA*, *nsdD* and *vosA* between *sclB* mutants and the wildtype during vegetative growth, indicating that SclB does not regulate conidiation through repression of conidiation-repressors. RNA was extracted from submerged cultures. B) *sclB* is epistatic towards *abaA*. Strains were point inoculated and grown for 3 days in light or dark at 37°C.
(TIF)

S4 Fig *flb* knock out phenotypes are epistatic to the *sclB* OE phenotype. *sclB* was overexpressed in *flb* knock out mutants. Strains were point inoculated on solid MM and grown for 3 days in light. *sclB* OE is not sufficient to rescue Δ*flb* phenotypes, showing that SclB does not act downstream of the *flb* factors. PMG = photomicrograph, bars = 200 μm.
(TIF)

S5 Fig SclB regulates secondary metabolite production. Full chromatogram of the compounds extracted from asexually grown cultures after three days growth is shown. 1 = austinol, 2 = dehydroaustinol, employed detector = ELSD.
(TIF)

S6 Fig **SclB regulates biosynthesis of emericellamide A, C and D.** Emericellamide A (A), C and D (B) were identified from HPLC-MS data according to their masses, fragmentation pattern and UV/VIS spectra [85]. B) For a better overview fragmentation pattern are not presented in the same intensity (WT and Δ*sclB* comp are 4-fold and Δ*sclB* 10-fold zoomed in compared to *sclB* OE).
(TIF)

S7 Fig SclB regulates genes of the oxidative stress defense in *A. nidulans*. qRT-PCR indicates that expression of *grlA* might be indirectly regulated by *sclB* (***P<0.001). *sclB* OE is able to induce *trxA* expression in response to H_2_O_2_ (***P<0.001). *napA* is not regulated by *sclB* in response to H_2_O_2_. Strains were grown vegetatively for 24 h and subsequently liquid cultures were incubated for 30 min with (grey boxes) or without (black boxes) 5 mM H_2_O_2_.
(TIF)

S8 Fig GFP-fusion proteins of SclB are functional and phosphorylated and Bi-FC controls are negative. A) Strains expressing SclB either N- or C-terminally tagged with sGFP in Δ*sclB* background, Δ*sclB* and wildtype (WT) were point inoculated on solid MM and grown for 4 days in light. B) SclB-GFP and GFP-SclB fusion proteins expressed under native promoter are visualized in a western hybridization assay employing an α-GFP antibody (GFP) and Ponceau staining as loading control (Pnc). The black arrow indicates bands corresponding to full-length fusion proteins (*in silico* prediction 87.46 kDa). C) Protein crude extracts of GFP-SclB grown vegetatively were mixed with phosphatase inhibitor cocktail (-/PhoI), with Lambda phosphatase (λ/-), or Lambda phosphatase and phosphatase inhibitor cocktail (λ/PhoI). A control sample was left untreated (-/-). A subsequent western hybridization assay employing α-GFP antibody visualizes protein bands. D) Two strains, either expressing *sclB*::*cyfp* and the free second half of the split YFP (*nyfp*; upper part), or free *cyfp* and *rcoA*::*nyfp* (lower part), under control of a bi-directional nitrate promoter were constructed. Strains were inoculated in liquid MM and analyzed with fluorescence microscopy after 36 h at 30°C.
(TIF)

S9 Fig SclB is phosphorylated at S327, T464 and S506 during vegetative growth. A) Phosphopeptides of SclB identified by LC-MS/MS. Mascot ionscores, SequestHT xcorr scores and phosphoRS site probabilities are given. Peptide sequences indicate identified b and y ions. B) Strains were created in Δ*sclB* background, in which the three identified residues of SclB, which are phosphorylated during vegetative growth, and two adjacent serines S504 and S505, are exchanged to alanine (*sclB*^S327A,T464A,S504-506A^) or aspartic acid (*sclB*^S327D,T464D,S506D^). Phenotypic analyses of strains grown for 3 days in light and dark show that both phosphorylation mutant strains complement wildtype phenotype.
(TIF)

S1 Table Differentially regulated genes with more than twofold induction (upregulated) or reduction (downregulated) in *ΔsclB* compared to wildtype. Only genes with a false discovery rate (fdr; Benjamini-Hochberg corrected p-value) < 0.5 were accepted.
(XLS)

S2 Table Comprehensive list of proteins identified with LCMS form GFP-trap pull-downs with sGFP-tagged SclB (sGFP-SclB and SclB-sGFP) as bait. Proteins were identified in at least two out of three biological replicates with a threshold of 3 ≥ MS/MS counts and 3 ≥ unique peptides [152], and sorted according to functional groups. Proteins identified solely in vegetative samples are highlighted in blue, proteins identified solely in developmental samples are given in green, proteins identified in vegetative and developmental samples are given in orange. Sys. Name = systematic name, std. name = standard name, ident. in = identified in, v = vegetative, a = asexual growth promoting conditions, s = sexual growth promoting conditions, unchar. = uncharacterized ^1^ = SclB was used as bait. Protein descriptions given are derived from AspGD [64].
(DOCX)

S3 Table Fungal strains used in this study. Most strains were constructed by employing of recyclable marker cassettes (see material and methods section in the main text), which leaves only a small *six* site (100 nucleotides) as scar after recycling of the marker off the genome. FGSC = Fungal genetics stock center, Kansas, USA.
(DOCX)

S4 Table *E. coli* strains used in this study. (DOCX)

S5 Table Plasmids used in this study. natRM = nourseothricin recyclable marker cassette, phleoRM = phleomycin recyclable marker cassette, ptrARM = pyrithiamine recyclable marker cassette, AN = *A*. *nidulans*, Afu = *A*. *fumigatus*.
(DOCX)

S6 Table Oligonucleotides used in this study. Primers listed in this table are given with description of their purpose. Primers designed for usage with a seamless cloning kit (SCK). *Mss*I sites, introduced by respective 5’ FW and 3’ rev primers, were chosen the way that no scar occurs after transformation into *A*. *nidulans* (i. e. primers were designed according to naturally occurring halves of the *Pme*I sites). FW = forward, rev = reverse, RT = qRT-Primer.
(DOCX)

S7 Table SclB shares its C6 architecture with 5.7% of all *A. nidulans* C6 proteins. The table summarizes all C6 architectures present in *A*. *nidulans* according to Wortman and collaborators, updated with two additional proteins found in database searches in AspGD and FungiDB [63,64,73]. Characterized representatives of the individual architectural groups are given and SclB’s architectural group is highlighted in yellow.
(XLSX)
