# Correction: Velvet domain protein VosA represses the zinc cluster transcription factor SclB regulatory network for *Aspergillus nidulans* asexual development, oxidative stress response and secondary metabolism

**DOI:** 10.1371/journal.pgen.1007638

**Published:** 2018-08-29

**Authors:** Karl G. Thieme, Jennifer Gerke, Christoph Sasse, Oliver Valerius, Sabine Thieme, Razieh Karimi, Antje K. Heinrich, Florian Finkernagel, Kristina Smith, Helge B. Bode, Michael Freitag, Arthur F. J. Ram, Gerhard H. Braus

There is an error in panel D of S8 Fig. Specifically, the upper panel should read ‘SclB-cYFP + nYFP’, not ‘SclA-cYFP + nYFP’. The authors have provided a corrected version here.

## Supporting information

S8 Fig GFP-fusion proteins of SclB are functional and phosphorylated and Bi-FC controls are negative. A) Strains expressing SclB either N- or C-terminally tagged with sGFP in Δ*sclB* background, Δ*sclB* and wildtype (WT) were point inoculated on solid MM and grown for 4 days in light. B) SclB-GFP and GFP-SclB fusion proteins expressed under native promoter are visualized in a western hybridization assay employing an α-GFP antibody (GFP) and Ponceau staining as loading control (Pnc). The black arrow indicates bands corresponding to full-length fusion proteins (*in silico* prediction 87.46 kDa). C) Protein crude extracts of GFP-SclB grown vegetatively were mixed with phosphatase inhibitor cocktail (-/PhoI), with Lambda phosphatase (λ/-), or Lambda phosphatase and phosphatase inhibitor cocktail (λ/PhoI). A control sample was left untreated (-/-). A subsequent western hybridization assay employing α-GFP antibody visualizes protein bands. D) Two strains, either expressing *sclB*::*cyfp* and the free second half of the split YFP (*nyfp*; upper part), or free *cyfp* and *rcoA*::*nyfp* (lower part), under control of a bi-directional nitrate promoter were constructed. Strains were inoculated in liquid MM and analyzed with fluorescence microscopy after 36 h at 30°C.
(TIF)
