# Supplementary figures and images for: Velvet domain protein VosA represses the zinc cluster transcription factor SclB regulatory network for Aspergillus nidulans asexual development, oxidative stress response and secondary metabolism
Source: PLoS Genet. 2018 Jul 25;14(7):e1007511. doi: 10.1371/journal.pgen.1007511 (PMC6078315; doi:10.1371/journal.pgen.1007511)

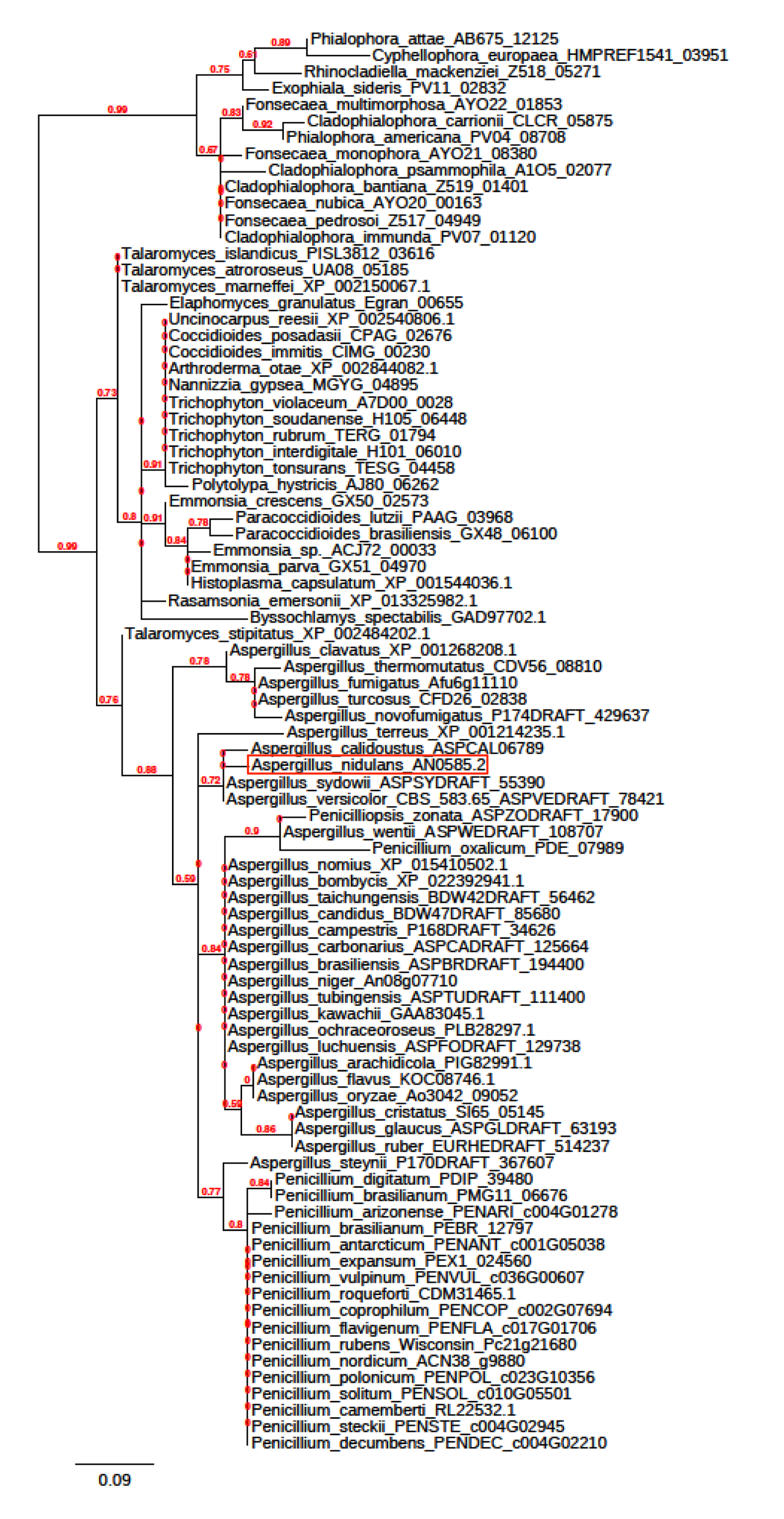

Supplement: S1 Fig — The C6 domain of SclB from A. nidulans was used for an in silico BLAST database search and C6 domains were aligned [59] for orthologs among the fungal kingdom. Phylogenetic analyses were conducted using a set of phylogeny programs comprising MUSCLE, Gblocks, PhyML and TreeDyn [57,58]. (TIF) [file pgen.1007511.s001.tif]

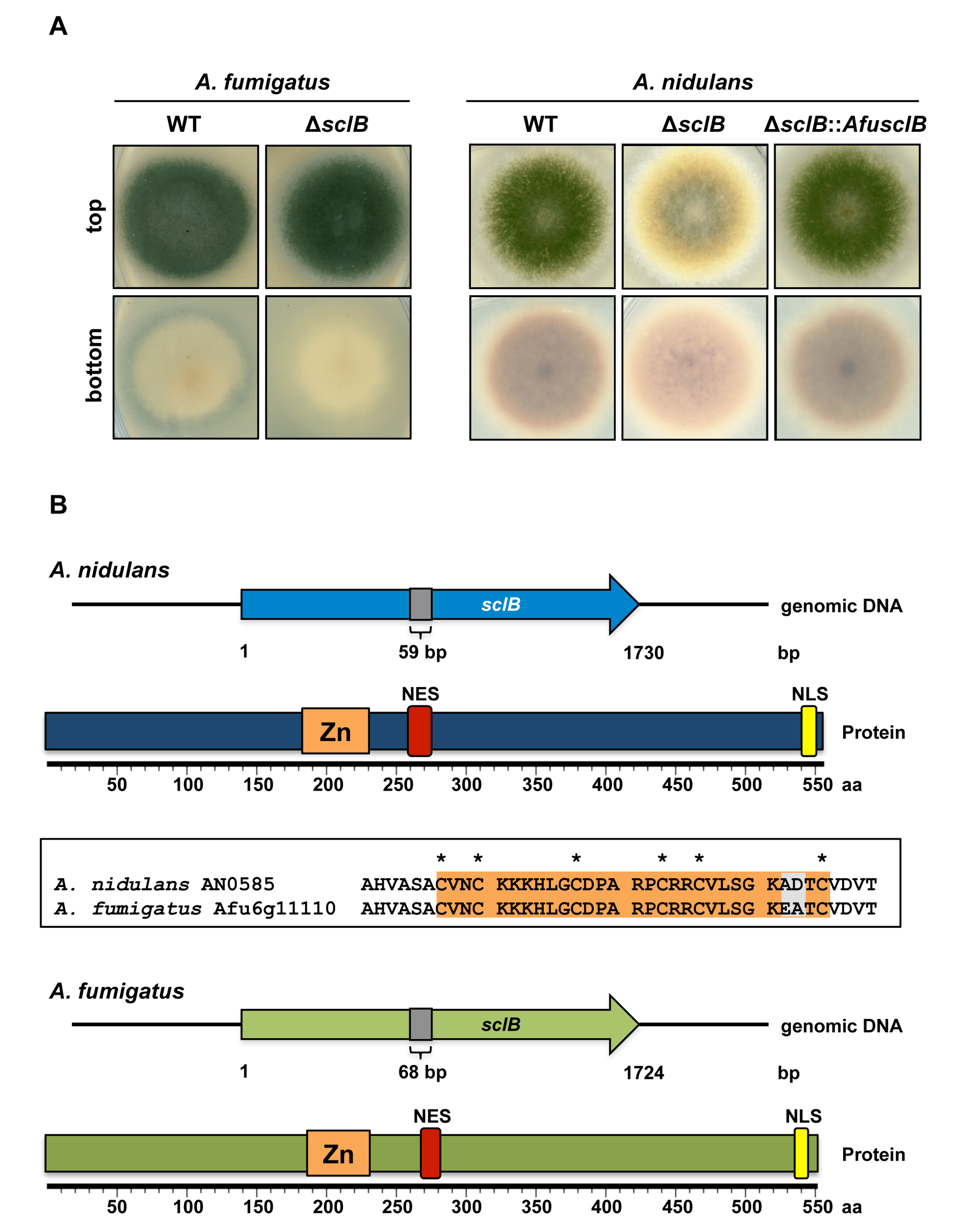

Supplement: S2 Fig — A) Loss of sclB does not result in an obvious conidiation phenotype in A. fumigatus (left side). Integration of the sclB ORF of A. fumigatus into an A. nidulans ΔsclB strain results in complementation of the wildtype phenotype (ΔsclB::AfusclB, right side). Strains were grown on solid MM for 3 days at 37°C. B) Schematic depiction of the sclB ORF from A. nidulans and A. fumigatus and their respective gene products. Grey boxes represent introns, bp = base pairs, Zn = C6 domain, NLS = nuclear localization sequence, NES = nuclear export signal, aa = amino acids. An alignment of the C6 domains (highlighted in orange) and adjacent residues of both proteins is shown in the middle. Asterisks indicate the six conserved cysteine residues of the C6 domain. The C6 domain (highlighted in orange) is highly conserved between both fungi with only two exceptions (in grey). (TIF) [file pgen.1007511.s002.tif]

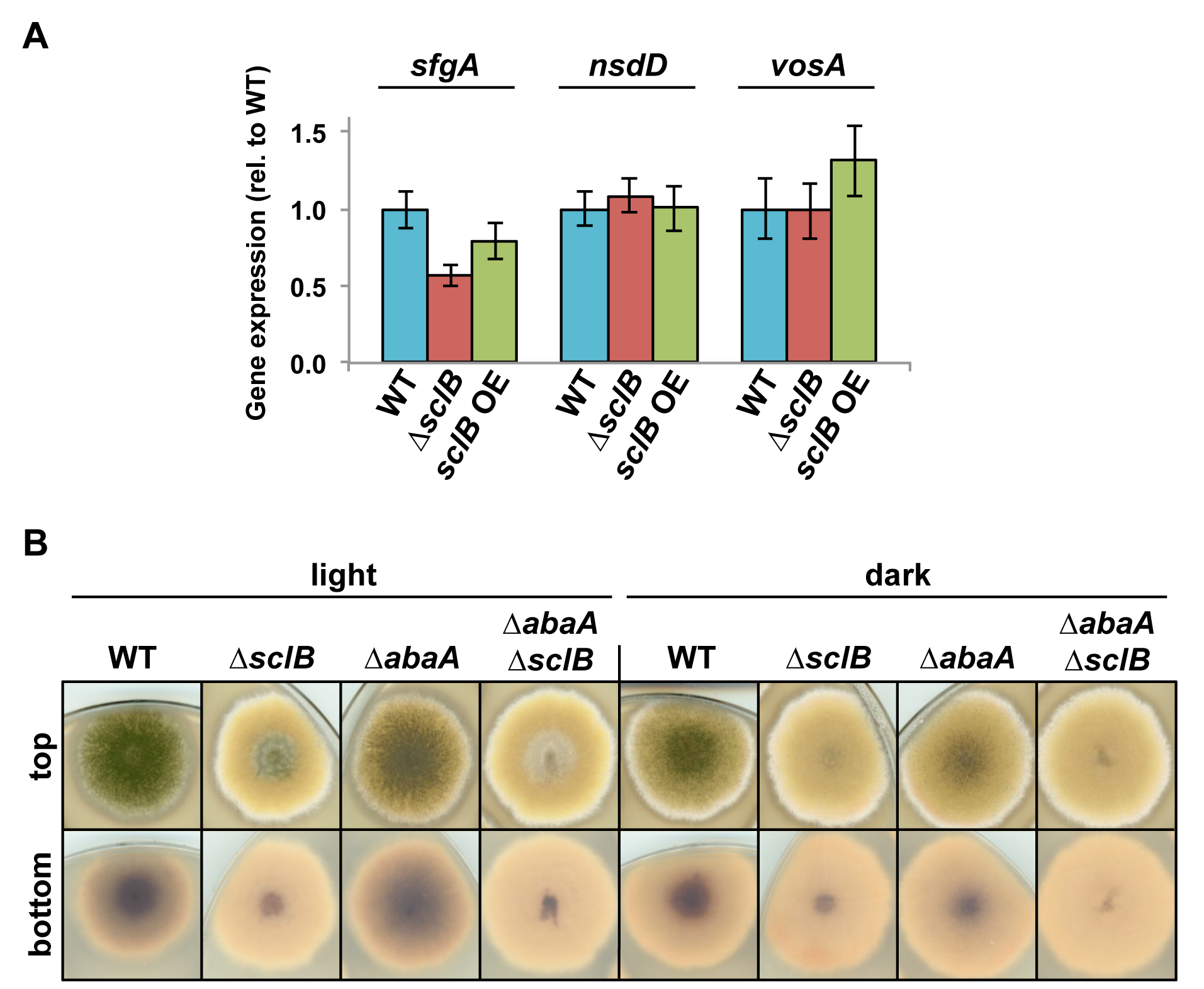

Supplement: S3 Fig — A) qRT-PCR shows no differences in gene expression of sfgA, nsdD and vosA between sclB mutants and the wildtype during vegetative growth, indicating that SclB does not regulate conidiation through repression of conidiation-repressors. RNA was extracted from submerged cultures. B) sclB is epistatic towards abaA. Strains were point inoculated and grown for 3 days in light or dark at 37°C. (TIF) [file pgen.1007511.s003.tif]

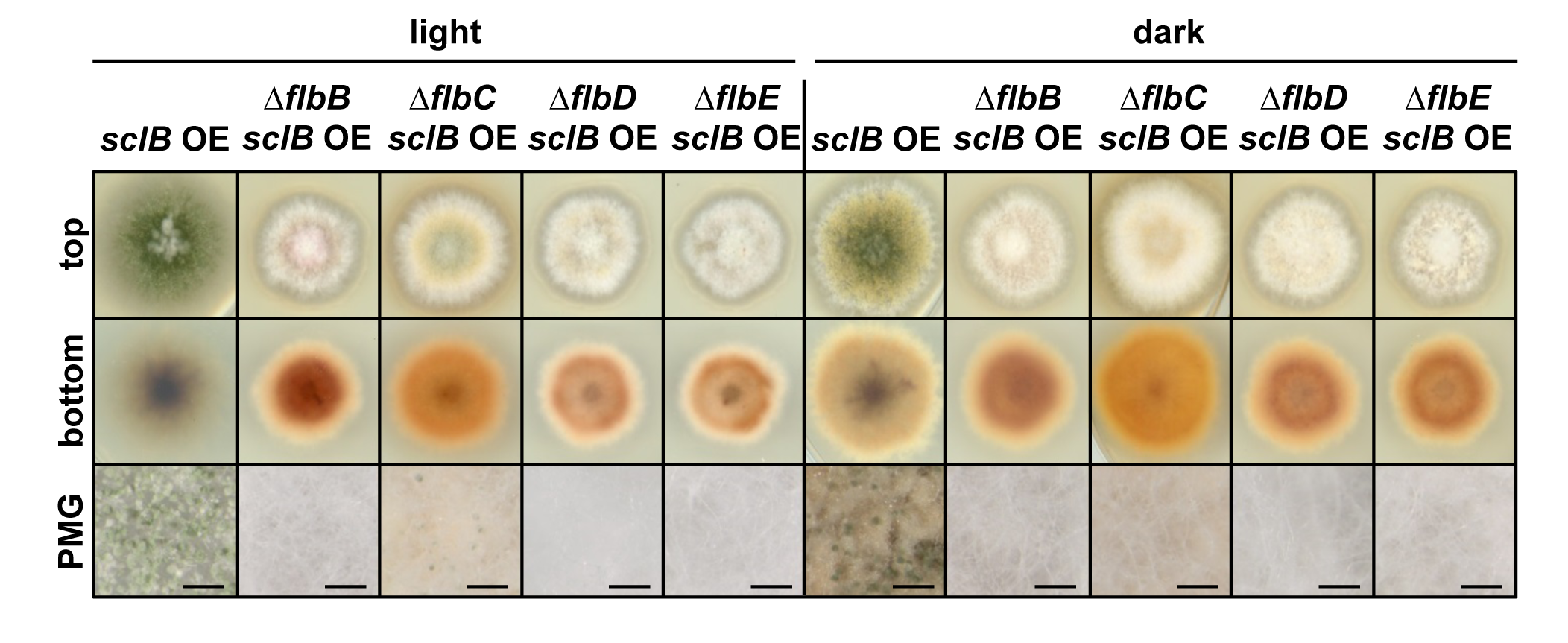

Supplement: S4 Fig — sclB was overexpressed in flb knock out mutants. Strains were point inoculated on solid MM and grown for 3 days in light. sclB OE is not sufficient to rescue Δflb phenotypes, showing that SclB does not act downstream of the flb factors. PMG = photomicrograph, bars = 200 μm. (TIF) [file pgen.1007511.s004.tif]

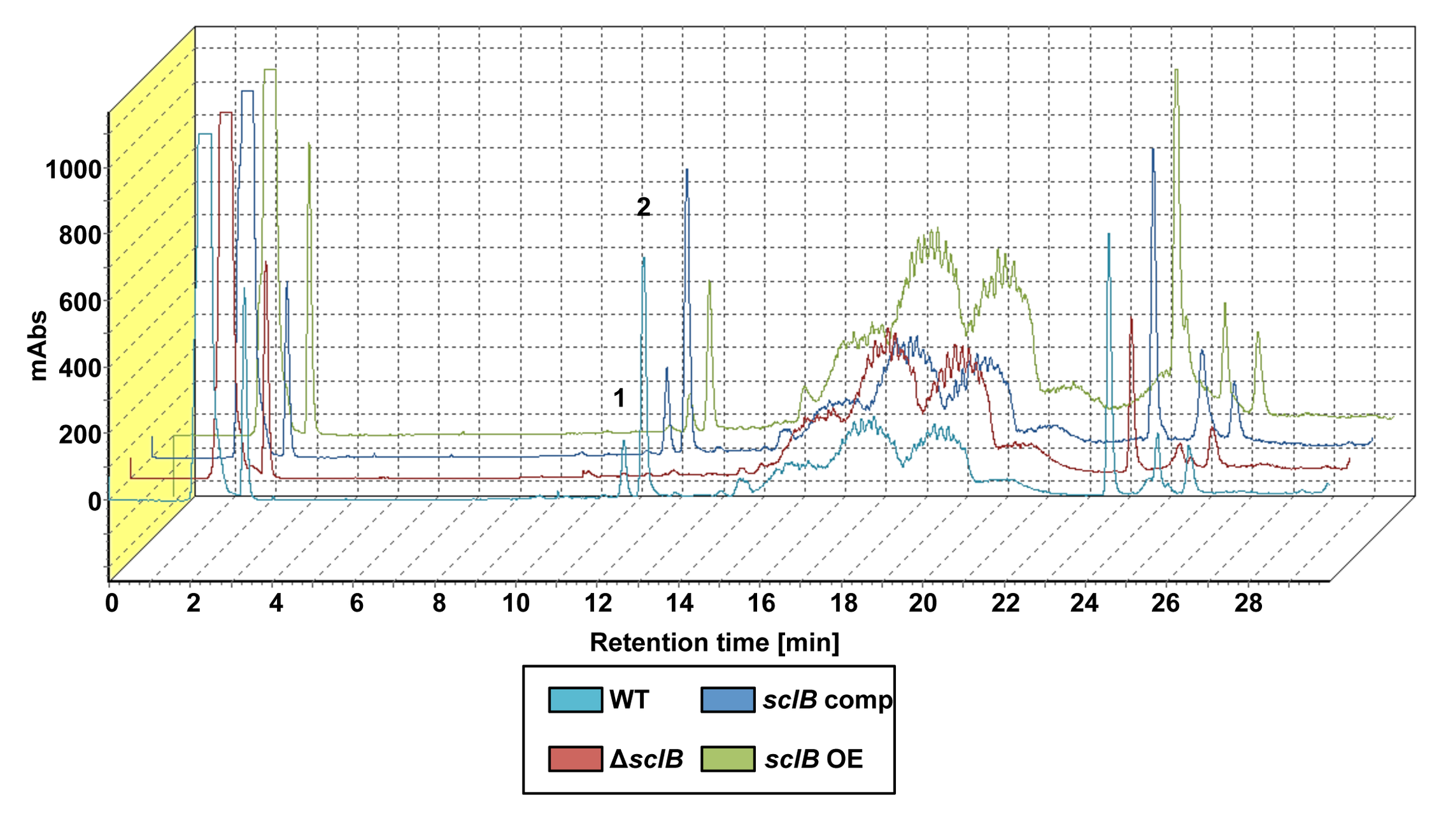

Supplement: S5 Fig — Full chromatogram of the compounds extracted from asexually grown cultures after three days growth is shown. 1 = austinol, 2 = dehydroaustinol, employed detector = ELSD. (TIF) [file pgen.1007511.s005.tif]

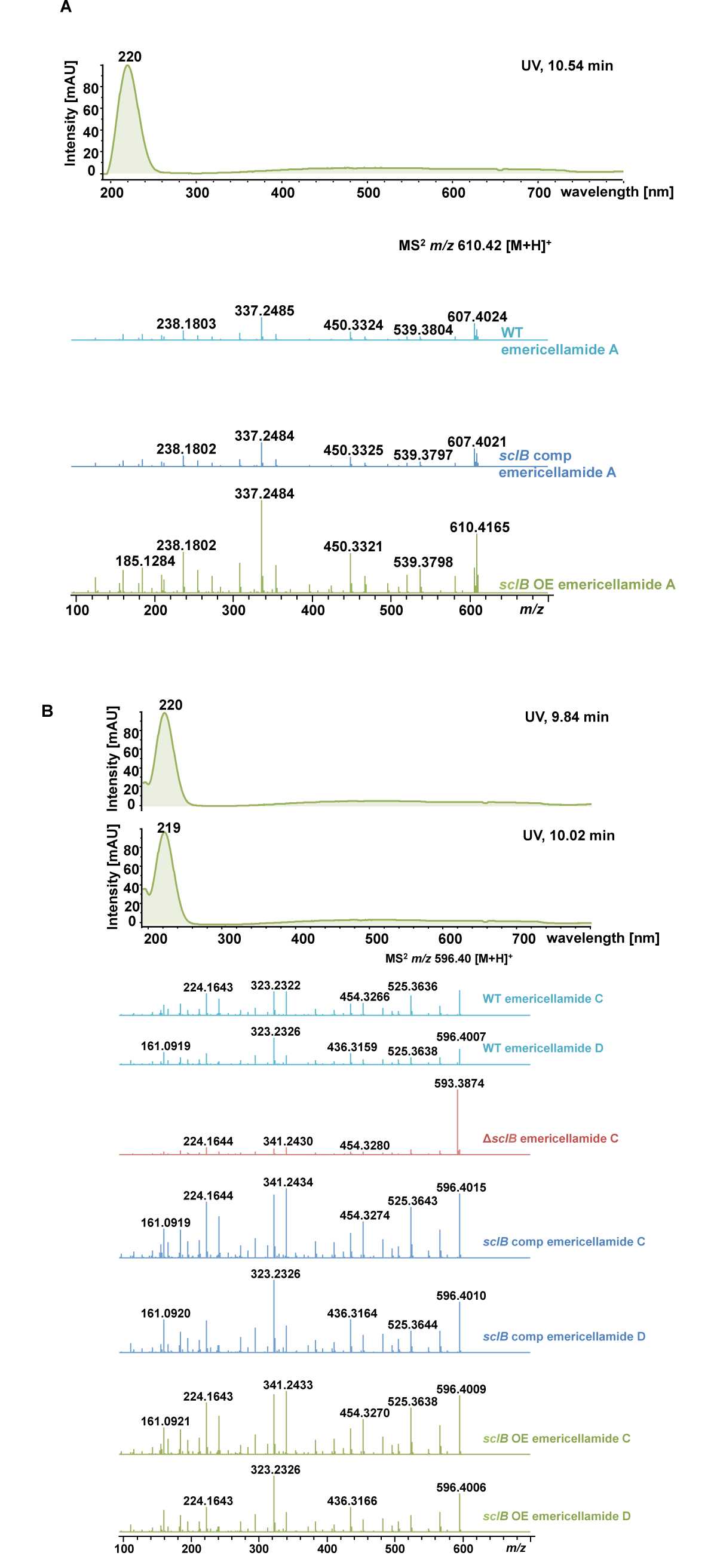

Supplement: S6 Fig — SclB regulates biosynthesis of emericellamide A, C and D. Emericellamide A (A), C and D (B) were identified from HPLC-MS data according to their masses, fragmentation pattern and UV/VIS spectra [85]. B) For a better overview fragmentation pattern are not presented in the same intensity (WT and ΔsclB comp are 4-fold and ΔsclB 10-fold zoomed in compared to sclB OE). (TIF) [file pgen.1007511.s006.tif]

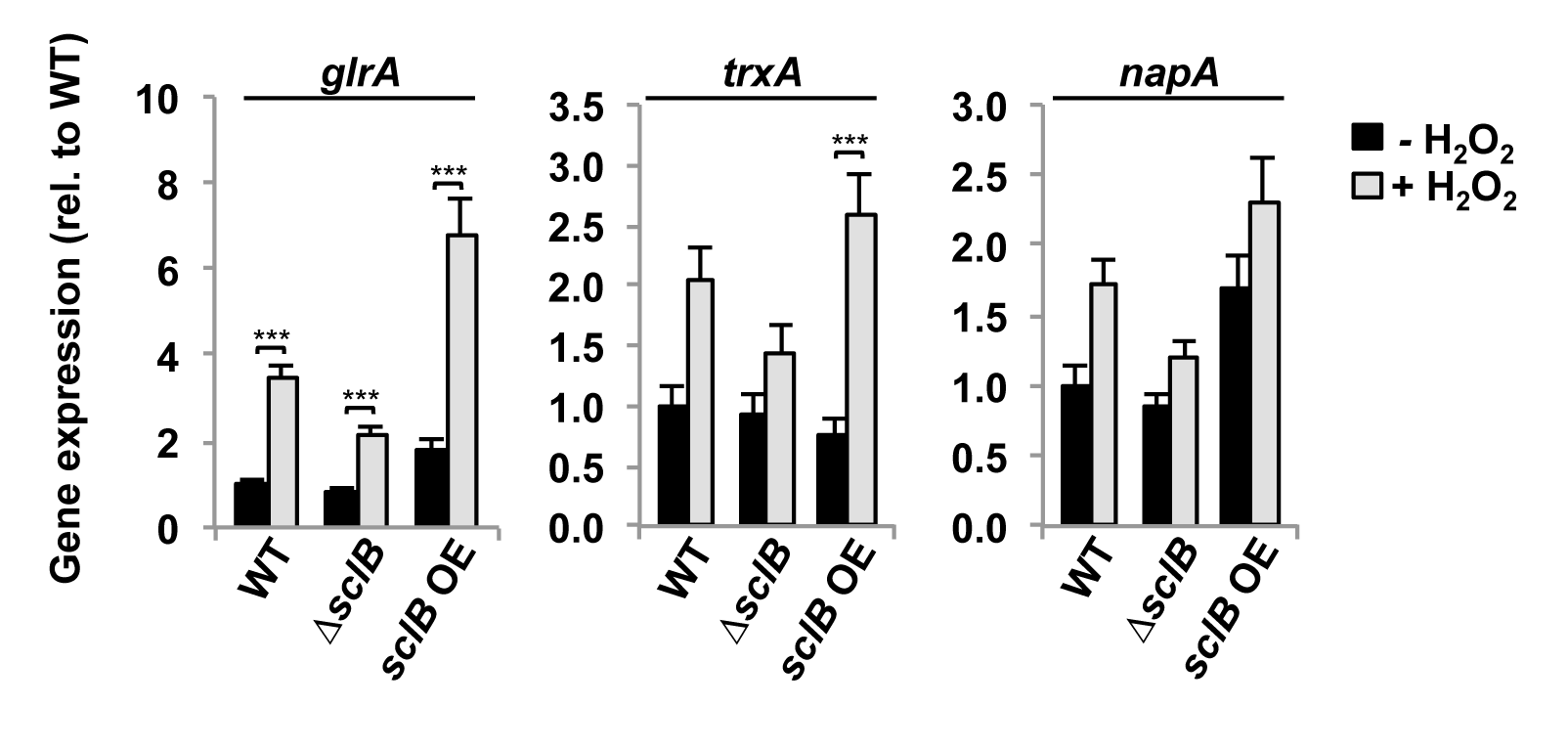

Supplement: S7 Fig — qRT-PCR indicates that expression of grlA might be indirectly regulated by sclB (***P<0.001). sclB OE is able to induce trxA expression in response to H2O2 (***P<0.001). napA is not regulated by sclB in response to H2O2. Strains were grown vegetatively for 24 h and subsequently liquid cultures were incubated for 30 min with (grey boxes) or without (black boxes) 5 mM H2O2. (TIF) [file pgen.1007511.s007.tif]

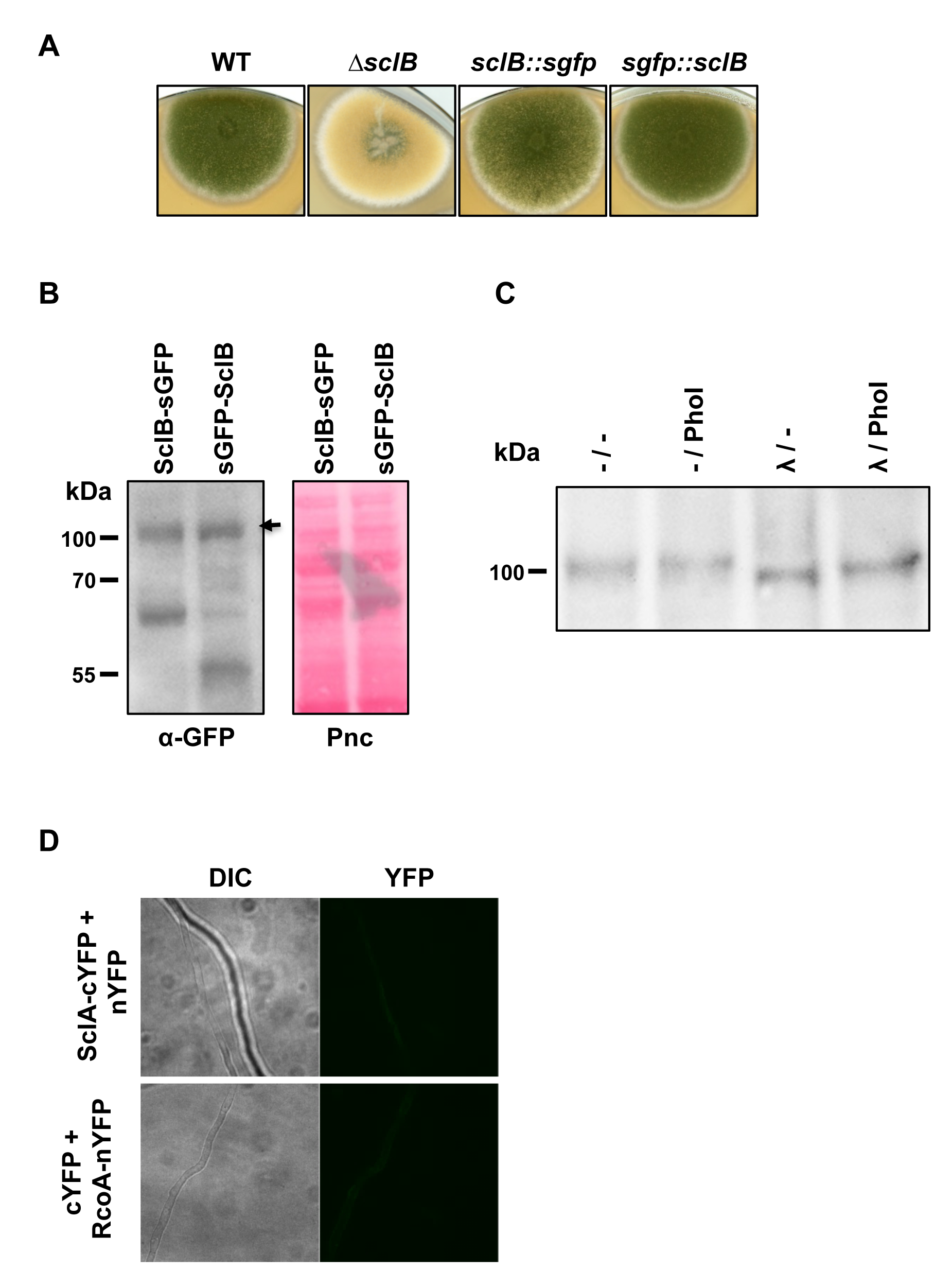

Supplement: S8 Fig — A) Strains expressing SclB either N- or C-terminally tagged with sGFP in ΔsclB background, ΔsclB and wildtype (WT) were point inoculated on solid MM and grown for 4 days in light. B) SclB-GFP and GFP-SclB fusion proteins expressed under native promoter are visualized in a western hybridization assay employing an α-GFP antibody (GFP) and Ponceau staining as loading control (Pnc). The black arrow indicates bands corresponding to full-length fusion proteins (in silico prediction 87.46 kDa). C) Protein crude extracts of GFP-SclB grown vegetatively were mixed with phosphatase inhibitor cocktail (-/PhoI), with Lambda phosphatase (λ/-), or Lambda phosphatase and phosphatase inhibitor cocktail (λ/PhoI). A control sample was left untreated (-/-). A subsequent western hybridization assay employing α-GFP antibody visualizes protein bands. D) Two strains, either expressing sclB::cyfp and the free second half of the split YFP (nyfp; upper part), or free cyfp and rcoA::nyfp (lower part), under control of a bi-directional nitrate promoter were constructed. Strains were inoculated in liquid MM and analyzed with fluorescence microscopy after 36 h at 30°C. (TIF) [file pgen.1007511.s008.tif]

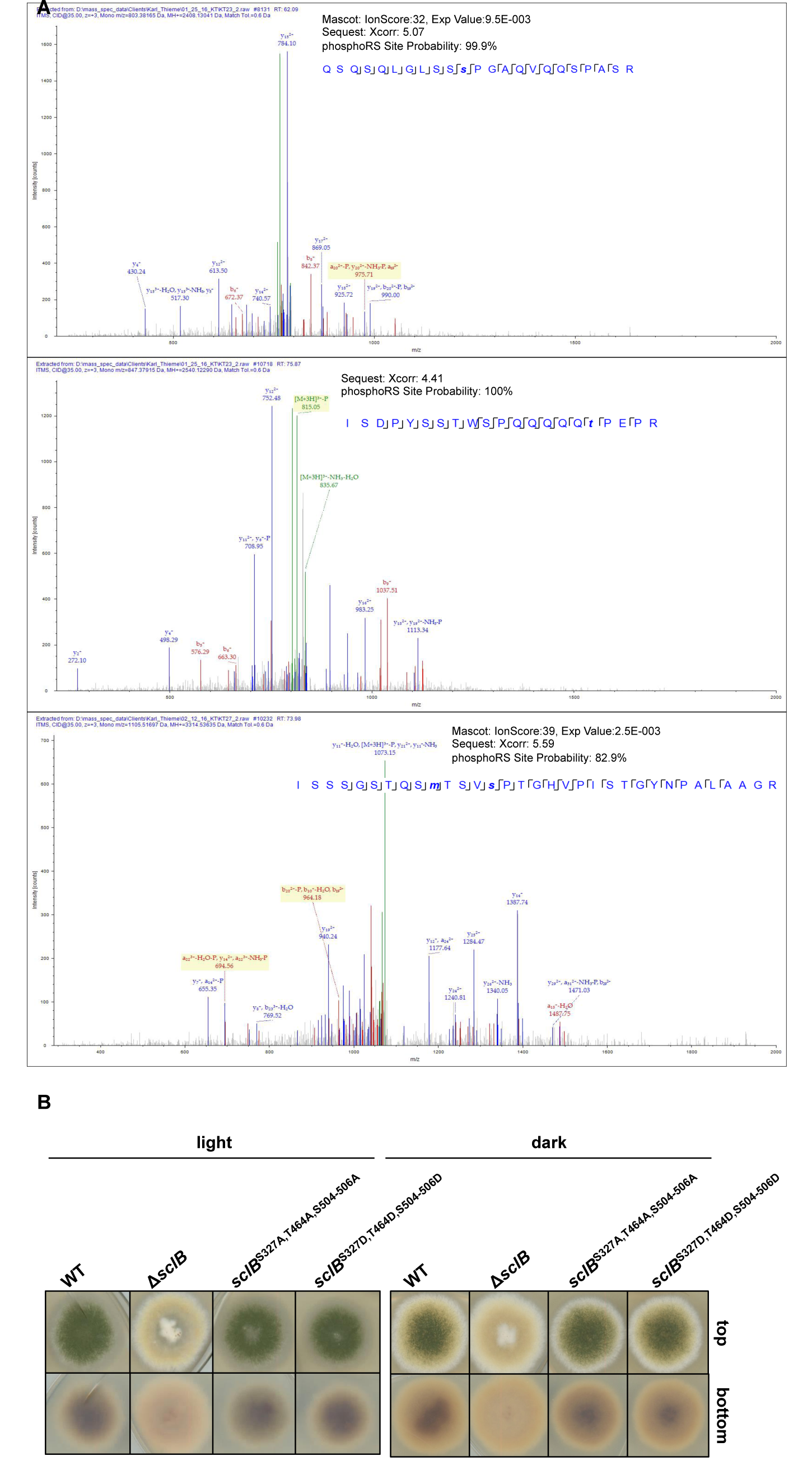

Supplement: S9 Fig — A) Phosphopeptides of SclB identified by LC-MS/MS. Mascot ionscores, SequestHT xcorr scores and phosphoRS site probabilities are given. Peptide sequences indicate identified b and y ions. B) Strains were created in ΔsclB background, in which the three identified residues of SclB, which are phosphorylated during vegetative growth, and two adjacent serines S504 and S505, are exchanged to alanine (sclBS327A,T464A,S504-506A) or aspartic acid (sclBS327D,T464D,S506D). Phenotypic analyses of strains grown for 3 days in light and dark show that both phosphorylation mutant strains complement wildtype phenotype. (TIF) [file pgen.1007511.s009.tif]
