# Supplementary material for: Velvet domain protein VosA represses the zinc cluster transcription factor SclB regulatory network for Aspergillus nidulans asexual development, oxidative stress response and secondary metabolism
Source: PLoS Genet. 2018 Jul 25;14(7):e1007511. doi: 10.1371/journal.pgen.1007511 (PMC6078315; doi:10.1371/journal.pgen.1007511)
Supplement: S2 Table — Proteins were identified in at least two out of three biological replicates with a threshold of 3 ≥ MS/MS counts and 3 ≥ unique peptides [152], and sorted according to functional groups. Proteins identified solely in vegetative samples are highlighted in blue, proteins identified solely in developmental samples are given in green, proteins identified in vegetative and developmental samples are given in orange. Sys. Name = systematic name, std. name = standard name, ident. in = identified in, v = vegetative, a = asexual growth promoting conditions, s = sexual growth promoting conditions, unchar. = uncharacterized 1 = SclB was used as bait. Protein descriptions given are derived from AspGD [64]. (DOCX) [file pgen.1007511.s011.docx]

**S2 Table**. Comprehensive list of proteins identified with LCMS form GFP-trap pull-downs with sGFP-tagged SclB (sGFP-SclB and SclB-sGFP) as bait. Proteins were identified in at least two out of three biological replicates with a threshold of 3 ≥ MS/MS counts and 3 ≥ unique peptides [152], and sorted according to functional groups. Proteins identified solely in vegetative samples are highlighted in blue, proteins identified solely in developmental samples are given in green, proteins identified in vegetative and developmental samples are given in orange. Sys. Name = systematic name, std. name = standard name, ident. in = identified in, v = vegetative, a = asexual growth promoting conditions, s = sexual growth promoting conditions, unchar. = uncharacterized ^1^ = SclB was used as bait. Protein descriptions given are derived from AspGD [64].

|  |  | |  | | | | | | | |  | |
| --- | --- | --- | --- | --- | --- | --- | --- | --- | --- | --- | --- | --- |
| **Sys. name** | **Std.name** | | **Description** | | | | | | | | **Ident. in** | |
|  |  | |  | | | | | | | |  | |
| **Bait** |  | |  | | | | | | | |  | |
| AN0585 | SclB^1^ | | Activator of asexual development and secondary metabolism | | | | | | | | v, a, s | |
|  |  | |  | | | | | | | |  | |
| **Nuclear transport** | | |  | | | | | | | |  | |
| AN6734 | KapF | | Essential karyopherin (importin) | | | | | | | | v | |
| AN2120 | KapJ | | Karyopherin (importin) | | | | | | | | v, a | |
| AN5717 | KapI | | Non-essential karyopherin family protein; required for normal hyphal growth and conidial development | | | | | | | | a, s | |
| AN0906 | KapB | | Essential karyopherin (importin) | | | | | | | | a, s | |
|  |  | |  | | | | | | | |  | |
| **Transcription/chromatin** | | | | |  | | | | | |  | |
| AN2012 | RfeF | | Putative transcription factor | | | | | | | | v | |
| AN6505 | RcoA | | Tup1 homolog of *S. cerevisiae* repressor domain, WD40 repeat protein; required for sexual development and for sterigmatocystin production, RcoA-SsnF repressor complex member | | | | | | | | v, s | |
| AN6705 | unchar. | | Ortholog of *S. pombe* Ssr2 (subunit of chromatin remodeling complex) | | | | | | | | v | |
| AN5102 | unchar. | | Putative ortholog of *S. pombe* Spt16 (FACT complex subunit) | | | | | | | | s | |
| AN6687 | unchar. | | Putative ortholog of *S. pombe* Pob3 (FACT complex subunit) | | | | | | | | s | |
| **RNA processing** | | |  | | | | | | | |  | |
| AN10557 | unchar. | | Putative ATP-dependent RNA helicase, putative *A. fumigatus* ortholog Ded1 | | | | | | | | v | |
| AN5931 | unchar. | | Putative ATP-dependent RNA helicase, putative *S. cerevisiae* ortholog of Dbp2 | | | | | | | | v | |
| AN7659 | unchar. | | Putative ortholog of *S. pombe* Dbp5 (RNA helicase) | | | | | | | | v | |
| AN0646 | unchar. | | Putative ortholog of *S. pombe* Upf1 (ATP-dependent RNA helicase) | | | | | | | | s | |
| AN6004 | unchar. | | Protein with an RNA recognition motif, putative ortholog of *S. pombe* Vip1 | | | | | | | | v | |
| AN10257 | unchar. | | Putative ortholog of *C. albicans* Pbp2 (putative RNA binding protein) | | | | | | | | v | |
| AN2068 | unchar. | | Putative ortholog of *S. pombe* Vgl1 (RNA binding protein) | | | | | | | | v, a | |
| AN1408 | unchar. | | Putative U5 snRNP-specific protein, putative ortholog of *S. pombe* Cwf1 | | | | | | | | s | |
| AN0111 | unchar. | | Putative ortholog of *S. pombe* and *S. cerevisiae* Syf1 (pre-mRNA splicing factor) | | | | | | | | s | |
| AN0289 | unchar. | | Putative ortholog of *S. pombe* Cwf22 (pre-mRNA splicing factor) | | | | | | | | s | |
| AN1208 | unchar. | | Putative ortholog of *S. pombe* Prp5 (pre-mRNA splicing factor) | | | | | | | | s | |
| AN4523 | Prp8 | | Putative mRNA-splicing protein | | | | | | | | s | |
| AN0310 | unchar. | | Putative ortholog of *S. pombe* Pwp1 (RNA processing protein) | | | | | | | | s | |
| AN6906 | unchar. | | Putative ortholog of *S. pombe* Prp19 (pre-mRNA processing factor) | | | | | | | | s | |
| AN11052 | unchar. | | Putative ortholog of *S. pombe* Exo2 (exonuclease) | | | | | | | | v | |
|  |  | |  | | | | | | | |  | |
| **Translation** | |  |  | | | | | | | |  | |
| AN3413 | unchar. | | Putative ribosomal protein S2 and S5, putative ortholog of *S. cerevisiae* Rps2 | | | | | | | | a | |
| AN0074 | unchar. | | Putative ortholog of *S. cerevisiae* Ebp2 (required for 25S rRNA maturation and 60S ribosomal subunit assembly) | | | | | | | | s | |
| AN0247 | unchar. | | Putative ortholog of *S. pombe* Nat10 (ribosome biogenesis ATPase) | | | | | | | | s | |
| AN10740 | unchar. | | Ortholog of Afu2g07970 (60S ribosomal protein L19), putative ortholog of *S. pombe* Rpl1902 | | | | | | | | s | |
| AN1095 | unchar. | | Putative ortholog of *S. pombe* Mrpl10 (predicted ribosomal protein subunit L15) | | | | | | | | s | |
| AN3167 | Nop58 | | Putative ribosome biogenesis protein | | | | | | | | s | |
| AN6902 | unchar. | | Putative ribosomal protein, putative ortholog of *S. cerevisiae* Mrt4 | | | | | | | | s | |
| AN10182 | unchar. | | Putative translation initiation factor 3, subunit f (eIF-3f) | | | | | | | | v | |
| AN4038 | unchar. | | Putative translation initiation factor eIF5B | | | | | | | | v | |
| AN7540 | unchar. | | Putative ortholog of *S. pombe* Moe1 (translation initiation factor eIF3d) | | | | | | | | v | |
| AN6060 | unchar. | | Ortholog of *A. fumigatus* eukaryotic translation initiation factor subunit eIF-4F | | | | | | | | v | |
| AN7350 | unchar. | | Ortholog of *A. fumigatus* translation initiation factor 4B | | | | | | | | v | |
| AN1158 | unchar. | | Putative ortholog of *S. cerevisiae* Ssd1 (translational repressor) | | | | | | | | v, s | |
| AN10475 | unchar. | | Putative ortholog of *S. pombe* Wrs1 (tryptophan-tRNA ligase) | | | | | | | | v | |
| AN4086 | unchar. | | Putative ortholog of *S. pombe* Frs1 (phenylalanine-tRNA ligase) | | | | | | | | v | |
| AN8867 | unchar. | | Putative ortholog of *S. pombe* Srs1 (serine-tRNA ligase) | | | | | | | | v | |
| AN1913 | unchar. | | Putative lysyl-tRNA synthetase, putative ortholog of *S. pombe* Krs1 | | | | | | | | a | |
| AN8224 | unchar. | | Putative ortholog of *S. pombe* cytoplasmic glutamate-tRNA ligase Gus1 (predicted) | | | | | | | | a | |
| AN3702 | unchar. | | Putative ortholog of *S. pombe* Lrs1 (leucine-tRNA ligase) | | | | | | | | s | |
| AN0705 | unchar. | | Putative ortholog of *S. pombe* Irs1 (isoleucine-tRNA ligase) | | | | | | | | v, s | |
| AN10474 | unchar. | | Has domain(s) with predicted tRNA binding activity, putative ortholog of *S. cerevisiae* Arc1 | | | | | | | | v, s | |
|  |  | |  | | | | | | | |  | |
| **Protein folding/chaperons** | | | | | | | |  | | | | |
| AN0858 | Hsp104 | | Putative chaperone | | | | | | | | | v |
| AN5713 | Cct7/CctA | | Putative chaperonin complex component, TCP-1 eta subunit; ortholog of *S. cerevisiae* Cct7p | | | | | | | | | a |
| AN2149 | Cct1 | | Putative chaperonin complex component, TCP-1 alpha subunit; ortholog of *S. cerevisiae* Tcp1p | | | | | | | | | s |
| AN10351 | unchar. | | Putative ortholog of *S. pombe* Aap1 (aspartyl metalloaminopeptidase, chaperone-mediated protein folding) | | | | | | | | | v |
| AN3592 | ClxA | | Putative calnexin with a predicted role in protein folding and protein quality control on the ER membrane | | | | | | | | | v |
| AN4583 | Cyp7/Cpr6 | | Putative peptidyl-prolyl cis-trans isomerase D | | | | | | | | | v |
| AN8605 | Cyp1 | | Peptidyl-prolyl cis-trans isomerase (PPIase); cyclophilin | | | | | | | | | v |
|  |  | |  | | | | | | | | |  |
| **Protein degradation** | | |  | | | | | | | | |  |
| AN1700 | unchar. | | Putative 26S proteasome regulatory subunit (*S. pombe* Rpn2 ortholog) | | | | | | | | | v |
| AN1922 | unchar. | | Putative ortholog of *A. niger* RpnG (proteasome regulatory subunit) | | | | | | | | | v |
| AN2213 | unchar. | | Putative ortholog of *S. pombe* Rpt2 (proteasome regulatory subunit) | | | | | | | | | v |
| AN4236 | unchar. | | Putative 26S proteasome subunit (*S. pombe* Rpt5 ortholog) | | | | | | | | | v |
| AN4282 | unchar. | | Putative ortholog of *A. oryzae* AspB (lysine aminopeptidase) | | | | | | | | | v |
|  |  | |  | | | | | | | | |  |
| **Primary metabolism** | | |  | | | | | | | | |  |
| AN10901 | unchar. | | Putative ortholog of *S. pombe* Gcv2 (glycine cleavage complex subunit), one-carbon metabolic process | | | | | | | | | v |
| AN2873 | LysA | | Saccharopine dehydrogenase (NAD^+^, L-lysine-forming) | | | | | | | | | v |
| AN3031 | unchar. | | Putative threonine synthase, predicted role in glycine, serine, and threonine metabolism, putative ortholog of *S. cerevisiae* Thr4 | | | | | | | | | v |
| AN5610 | unchar. | | Putative L-aminoadipate-semialdehyde dehydrogenase, predicted role in lysine metabolism, putative ortholog of *S. pombe* Lys1 | | | | | | | | | v |
| AN6639 | McdB | | Putative 2-methylcitrate dehydratase, predicted role in lysine metabolism | | | | | | | | | v |
| AN0708 | AromA | | Putative pentafunctional AROM polypeptide with 3-dehydroquinate synthase, 3-dehydroquinate dehydratase, shikimate 5-dehydrogenase, shikimate kinase, and EPSP synthase activities, predicted role in aromatic amino acid biosynthesis | | | | | | | | | a |
| AN7451 | GdhB | | Putative NAD-glutamate dehydrogenase, predicted role in glutamate and glutamine metabolism | | | | | | | | | a |
| AN2964 | PdhX | | Pyruvate dehydrogenase complex component | | | | | | | | | v |
| AN3829 | unchar. | | Putative succinate-semialdehyde dehydrogenase [NAD(P)^+^], putative ortholog of *A. fumigatus* Uga2 | | | | | | | | | v |
| AN3894 | unchar. | | Putative aconitate hydratase, predicted role in the TCA cycle, putative ortholog of *S. cerevisiae* Aco2 | | | | | | | | | v |
| AN3901 | unchar. | | Putative lactic acid dehydrogenase, predicted role in energy metabolism, putative ortholog of *S. cerevisiae* Cyb2 | | | | | | | | | v |
| AN6525 | AciA | | Formate dehydrogenase, predicted role in oxalic acid metabolism | | | | | | | | | v |
| AN0034 | unchar. | | Putative glycerone kinase, predicted role in glycerol metabolism, putative ortholog of *N. crassa* Dak1 | | | | | | | | | a |
| AN0565 | PyrABCN | | Multifunctional enzyme with carbamoyl-phosphate synthase and aspartate carbamoyltransferase activities | | | | | | | | | a |
| AN0567 | unchar. | | Putative alcohol oxidase, predicted role in glycerol metabolism | | | | | | | | | a |
| AN5162 | PdhB | | Putative pyruvate dehydrogenase (lipoamide), predicted role in pyruvate metabolism | | | | | | | | | a |
| AN7895 | CipB | | Oxidoreductase; contains Zn-dependent alcohol dehydrogenase domain | | | | | | | | | v, a, s |
| AN5716 | unchar. | | Putative inosine-5'-monophosphate dehydrogenase, predicted role in purine metabolism, putative ortholog of *S. pombe* Mug70 | | | | | | | | | v |
| AN6541 | AdF/Ad9 | | Putative ligase, predicted role in purine metabolism | | | | | | | | | v |
| AN1015 | unchar. | | Putative phosphorylase, predicted role in glycogen degradation, putative ortholog of *S. cerevisiae* Gph1 | | | | | | | | | v, a |
| AN8010 | unchar. | | Putative glycogen (starch) synthase, predicted role in glycogen biosynthesis, putative ortholog of *N. crassa* Gsy-1 | | | | | | | | | v, a, s |
|  |  | |  | | | | | | | | |  |
| **Secondary metabolism** | | | |  | | | | | | | |  |
| AN5130 | unchar. | | Putative ortholog of *A. niger* HemF (coproporphyrinogen III oxidase) | | | | | | | | | v |
| AN11008 | ErgA | | Putative ortholog of *S. pombe* Erg1 (squalene monooxygenase) | | | | | | | | | v |
| AN2943 | RfeA | | Putative reguatory role in secondary metabolism, protein kinase domain | | | | | | | | | a |
| AN8435 | unchar. | | Putative ortholog of *A. oryzae* tyrosinase MelB | | | | | | | | | a |
| AN7897 | DbaB | | FAD-binding monooxygenase with a role in secondary metabolism; member of the dba gene cluster | | | | | | | | | s |
| AN7902 | DbaH | | FAD-binding monooxygenase with a role in secondary metabolism; member of the dba gene cluster | | | | | | | | | s |
|  |  | |  | | | | | | | | |  |
| **Cell compartments/cytoskeleton/septa** | | | | | | | | | |  | | |
| AN9149 | unchar. | | Putative ortholog of *S. pombe* Tcb3 (ER-plasma membrane tethering protein) | | | | | | | | | v |
| AN0261 | Sec23 | | COPII coat component; considered a prototypic marker of transitional ER (endoplasmic reticulum) | | | | | | | | | v, a |
| AN8233 | unchar. | | Putative ortholog of *S. cerevisiae* Sfh5 (phosphatidylinositol transfer protein) | | | | | | | | | v, a |
| AN3720 | unchar. | | Putative ortholog of *S. pombe* Sec24 (COP II cargo receptor) | | | | | | | | | v |
| AN6257 | unchar. | | Putative ortholog of *A. oryzae* Sec31 (subunit of vesicle coat complex COPII, ER to Golgi transport) | | | | | | | | | a |
| AN1177 | unchar. | | Putative ortholog of *S. pombe* Sec26 (coatomer subunit, ER-Golgi transport) | | | | | | | | | s |
| AN4547 | unchar. | | Putative ortholog of *S. pombe* Sec21 (coatomer subunit, ER-Golgi transport) | | | | | | | | | s |
| AN8023 | VpsA | | Putative ortholog of *S. pombe* Vps1 (dynamin), required for vacuole biogenesis | | | | | | | | | v |
| AN7687 | unchar. | | Putative ortholog of *S. pombe* Tom70 (translocase receptor) | | | | | | | | | v |
| AN3843 | unchar. | | Putative ortholog of *S. cerevisiae* Mic60 (mitochondrial complex member) | | | | | | | | | v |
| AN4064 | unchar. | | Putative ADP/ATP carrier protein | | | | | | | | | a |
| AN4402 | unchar. | | Putative ortholog of *S. cerevisiae* Por1 mitochondrial porin | | | | | | | | | a |
| AN5803 | FimA | | Predicted fimbrin protein | | | | | | | | | v |
| AN8862 | MyoV | | Myosin V | | | | | | | | | v |
| AN6838 | TubC | | Beta-tubulin | | | | | | | | | a |
| AN6341 | unchar. | | Protein with similarity to *S. cerevisiae* Crn1p; predicted role in actin patch assembly | | | | | | | | | v, a |
| AN4667 | AspA | | Septin, involved in development; prevents formation of inappropriate germ tubes and branches; required for formation of normal conidiophores | | | | | | | | | a |
| AN6688 | AspB | | Putative septin B; localizes to septa during early septum formation and to branch points during vegetative growth; localizes to the vesicle/metula, the metula/phialide and the phialide/conidium interfaces during conidiophore development | | | | | | | | | a |
| AN4695 | HexA | | Putative Woronin body protein; HapX-regulated gene | | | | | | | | | v, s |
| AN3026 | CopA | | Alpha-COP coatamer-related protein involved in the establishment and maintenance of polarized growth | | | | | | | | | v, s |
| AN7111 | FoxA | | Peroxisomal multifunctional enzyme | | | | | | | | | a |
|  |  | |  | | | | | | | | |  |
| **Membranes/cell wall** | | | | | | |  | |  | | | |
| AN0595 | unchar. | | Putative NADPH-cytochrome P450 reductase with a predicted role in energy metabolism, putative ortholog of *S. pombe* Ccr1 | | | | | | | | | v |
| AN2210 | unchar. | | Probable ABC-transporter, putative ortholog of *S. cerevisiae* Arb1 | | | | | | | | | v |
| AN3163 | StoA | | Putative stomatin ortholog, predicted to have scaffolding functions in maintenance of lipid microdomains in membranes | | | | | | | | | v |
| AN6287 | unchar. | | Putative F1F0-ATPase complex subunit, predicted role in energy metabolism, putative ortholog of *A. niger* Atp5 | | | | | | | | | v |
| AN0317 | unchar. | | Putative ortholog of *S. cerevisiae* Ede1 (scaffold protein involved in endocytosis) | | | | | | | | | v |
| AN0870 | unchar. | | Putative transmembrane transporter with a predicted role in small molecule transport, putative ortholog of *S. cerevisiae* Mir1 | | | | | | | | | a |
| AN6232 | VmaB | | Putative F1F0-ATPase complex subunit, nitrogen and amino acid metabolism | | | | | | | | | s |
| AN12492 | unchar. | | Putative dynamin | | | | | | | | | s |
| AN2532 | unchar. | | Putative ortholog of *A. oryzae* AoxA (amine oxidase) | | | | | | | | | v |
| AN1911 | unchar. | | Putative mannose-1-phosphate guanyltransferase, putative ortholog of *S. pombe* Mpg2 | | | | | | | | | v |
| AN2314 | unchar. | | Putative 1,4-alpha-glucan branching enzyme, predicted role in starch metabolism, putative ortholog of *A. niger* GbeA | | | | | | | | | a |
| AN4727 | UgeA | | UDP-glucose 4-epimerase, involved in galactose metabolism | | | | | | | | | a |
| AN7657 | GelA | | Putative 1,3-beta-transglycosidase with a predicted role in glucan processing; predicted glycosyl phosphatidylinositol (GPI)-anchor | | | | | | | | | v, a |
|  |  | |  | | | | | | | | |  |
| **Oxidative stress response** | | | | | |  | | | | | | |
| AN9339 | CatB | | Hyphal catalase | | | | | | | | | v |
|  |  | |  | | | | | | | | |  |
| **Unknown function** | | |  | | | | | | | | |  |
| AN0753 | unchar. | | Protein of unknown function | | | | | | | | | v |
| AN1378 | unchar. | | Protein of unknown function | | | | | | | | | v |
| AN5141 | unchar. | | Protein of unknown function | | | | | | | | | v |
| AN7710 | unchar. | | Protein of unknown function | | | | | | | | | v |
| AN5421 | unchar. | | Protein of unknown function | | | | | | | | | a |
| AN5741 | unchar. | | Protein of unknown function | | | | | | | | | s |
| AN7014 | unchar. | | Protein of unknown function | | | | | | | | | s |
| AN7836 | unchar. | | Protein of unknown function | | | | | | | | | s |
| AN5446 | unchar. | | Protein of unknown function | | | | | | | | | v, s |
| AN2954 | unchar. | | Protein of unknown function | | | | | | | | | a, s |
