# Supplementary material for: Velvet domain protein VosA represses the zinc cluster transcription factor SclB regulatory network for Aspergillus nidulans asexual development, oxidative stress response and secondary metabolism
Source: PLoS Genet. 2018 Jul 25;14(7):e1007511. doi: 10.1371/journal.pgen.1007511 (PMC6078315; doi:10.1371/journal.pgen.1007511)
Supplement: S3 Table — Most strains were constructed by employing of recyclable marker cassettes (see material and methods section in the main text), which leaves only a small six site (100 nucleotides) as scar after recycling of the marker off the genome. FGSC = Fungal genetics stock center, Kansas, USA. (DOCX) [file pgen.1007511.s012.docx]

**S3 Table. Fungal strains used in this study.** Most strains were constructed by employing of recyclable marker cassettes (see material and methods section in the main text), which leaves only a small *six* site (100 nucleotides) as scar after recycling of the marker off the genome. FGSC = Fungal genetics stock center, Kansas, USA.

|  |  |  |
| --- | --- | --- |
| Strain name | Genotype | Reference |
| FGSC A4 | *veA*^+^ | FGSC |
| AGB551 | ∆*nkuA*::*argB*, *pyrG89*, *pyroA4, veA*^+^ | [1] |
| AGB596 | ^P^*gpdA*::*sgfp-phleo*^R^; *pabaA1*, *yA2*, *veA*^+^ | [1] |
| AGB1007 | ∆*nkuA*::*argB*, *pyrG89*, *pyroA4*, *veA*^+^, ∆*sclB*::*six* | This study |
| AGB1008 | ∆*nkuA*::*argB*, *pyrG89*, *pyroA4, veA*^+^, *six*::^P^*niaD*::*sclB* | This study |
| AGB1009 | ∆*nkuA*::*argB*, *pyrG89*, *pyroA4*, *veA*^+^, *sclB*::*sgfp*::*six* | This study |
| AGB1010 | ∆*nkuA*::*argB*, *pyrG89*, *pyroA4*, *veA*^+^, *sgfp*::*sclB*::*six* | This study |
| AGB1011 | ∆*nkuA*::*argB,* *pyrG89*, *pyroA4*, *veA*^+^, *sclB*::*six* | This study |
| AGB1012 | AGB1009, transformed with pME3173 | This study |
| AGB1013 | AGB1010, transformed with pME3173 | This study |
| AGB1014 | AGB551, transformed with pME3173 | This study |
| AGB1015 | ∆*nkuA*::*argB*, *pyrG89*, *pyroA4*, *veA*^+^, ^P^*sclB*::*sclB*^S327A,T464A,S504-506A^::*six* | This study |
| AGB1016 | ∆*nkuA*::*argB*, *pyrG89*, *pyroA4*, *veA*^+^, ∆*fluG*::*six* | This study |
| AGB1017 | ∆*nkuA*::*argB*, *pyrG89*, *pyroA4*, *veA*^+^, ∆*fluG*::*six*, ∆*sclB*::*six* | This study |
| AGB1018 | ∆*nkuA*::*argB*, *pyrG89*, *pyroA4*, *veA*^+^, ∆*fluG*::*six*, *six*::^P^*niaD*::*sclB* | This study |
| AGB1028 | ∆*nkuA*::*argB*, *pyrG89*, *pyroA4*, *veA*^+^, ∆*abaA*::*six* | This study |
| AGB1029 | ∆*nkuA*::*argB*, *pyrG89*, *pyroA4*, *veA*^+^, ∆*abaA*::*six*, ∆*sclB*::*six* | This study |
| AGB1031 | ∆*nkuA*::*argB*, *pyrG89*, *pyroA4*, *veA*^+^, ∆*brlA*::*six* | This study |
| AGB1032 | ∆*nkuA*::*argB*, *pyrG89*, *pyroA4*, *veA*^+^, ∆*brlA*::*six*, ∆*sclB*::*six* | This study |
| AGB1035 | ∆*nkuA*::*argB*, *pyrG89*, *pyroA4*, *veA*^+^, ∆*flbB*::*six* | This study |
| AGB1036 | ∆*nkuA*::*argB*, *pyrG89*, *pyroA4*, *veA*^+^, ∆*flbB*::*six*, ∆*sclB*::*six* | This study |
| AGB1037 | ∆*nkuA*::*argB*, *pyrG89*, *pyroA4*, *veA*^+^, ∆*flbB*::*six*, *six*::^P^*niaD*::*sclB* | This study |
| AGB1039 | ∆*nkuA*::*argB*, *pyrG89*, *pyroA4*, *veA*^+^, ∆*flbC*::*six* | This study |
| AGB1040 | ∆*nkuA*::*argB*, *pyrG89*, *pyroA4*, *veA*^+^, ∆*flbC*::*six*, ∆*sclB*::*six* | This study |
| AGB1041 | ∆*nkuA*::*argB*, *pyrG89*, *pyroA4*, *veA*^+^, ∆*flbC*::*six*, *six*::^P^*niaD*::*sclB* | This study |
| AGB1042 | ∆*nkuA*::*argB*, *pyrG89*, *pyroA4*, *veA*^+^, ∆*sclB*::*AfusclB* | This study |
| AGB1043 | ∆*nkuA*::*argB*, *pyrG89*, *pyroA4*, *veA*^+^, ∆*flbD*::*six* | This study |
| AGB1044 | ∆*nkuA*::*argB*, *pyrG89*, *pyroA4*, *veA*^+^, ∆*flbD*::*six*, ∆*sclB*::*six* | This study |
| AGB1045 | ∆*nkuA*::*argB*, *pyrG89*, *pyroA4*, *veA*^+^, ∆*flbD*::*six*, *six*::^P^*niaD*::*sclB* | This study |
| AGB1047 | ∆*nkuA*::*argB*, *pyrG89*, *pyroA4*, *veA*^+^, ∆*flbE*::*six* | This study |
| AGB1048 | ∆*nkuA*::*argB*, *pyrG89*, *pyroA4*, *veA*^+^, ∆*flbE*::*six*, ∆*sclB*::*six* | This study |
| AGB1049 | ∆*nkuA*::*argB*, *pyrG89*, *pyroA4*, *veA*^+^, ∆*flbE*::*six*, *six*::^P^*nia*D::*sclB* | This study |
| AGB1051 | ∆*nkuA*::*argB*, *pyrG89*, *pyroA4*, *veA*^+^, *phleo*^R^, ^P^*niaD*::*sclB*::*eyfp-C*, ^P^*niiA*::*rcoA*::*eyfp-N* | This study |
| AGB1052 | AGB1051, transformed with pME3173 | This study |
| AGB1053 | ∆*nkuA*::*argB*, *pyrG89*, *pyroA4*, *veA*^+^, *phleo*^R^, ^P^*niaD*::*sclB*::*eyfp-C*, ^P^*niiA*::*eyfp-N* | This study |
| AGB1054 | ∆*nkuA*::*argB*, *pyrG89*, *pyroA4*, *veA*^+^, *phleo*^R^, ^P^*niaD*::*eyfp-C*, ^P^*niiA*::*rcoA*::*eyfp-N* | This study |
| AGB1057 | ∆*nkuA*::*argB*, *pyrG89*, *pyroA4*, *veA^+^*, ∆*vosA*::*six* | This study |
| AGB1058 | ∆*nkuA*::*argB*, *pyrG89*, *pyroA4*, *veA^+^*, ∆*vosA*::*six*, ∆*sclB*::*six* | This study |
| AGB1059 | ∆*nkuA*::*argB*, *pyrG89*, *pyroA4*, *veA^+^*, ∆*vosA*::*six*, *six*::^P^*niaD*::*sclB* | This study |
| AGB1062 | ∆*nkuA*::*argB*, *pyrG89*, *pyroA4*, *veA*^+^, ∆*velC*::*six* | This study |
| AGB1063 | ∆*nkuA*::*argB*, *pyrG89*, *pyroA4*, *veA*^+^, ∆*velC*::*six*, ∆*sclB*::*six* | This study |
| AGB1064 | ∆*nkuA*::*argB*, *pyrG89*, *pyroA4*, *veA*^+^, ∆*velB*::*six* | This study |
| AGB1065 | ∆*nkuA*::*argB*, *pyrG89*, *pyroA4*, *veA*^+^, ∆*velB*::*six*, ∆*sclB*::*six* | This study |
| AGB1066 | ∆*nkuA*::*argB*, *pyrG89*, *pyroA4*, ∆*veA*::*six* | This study |
| AGB1067 | ∆*nkuA*::*argB*, *pyrG89*, *pyroA4*, ∆*veA*::*six*, ∆*sclB*::*six* | This study |
| AGB1147 | ∆*nkuA*::*argB*, *pyrG89*, *pyroA4*, *veA*^+^, ^P^*sclB*::*sclB*^S327D,T464D,S504-506D^::*six* | This study |
| AfS35 | ∆*akuA*::*loxP* | Derivative of AfS28 [2] |
| AfGB129 | ∆*akuA*::*loxP*, ∆*sclB*::*six* | This study |

1. Bayram Ö, Sarikaya-Bayram Ö, Ahmed YL, Maruyama J-I, Valerius O, Rizzoli SO, et al. The Aspergillus nidulans MAPK module AnSte11-Ste50-Ste7-Fus3 controls development and secondary metabolism. PLoS Genet. 2012;8:e1002816.

2. Krappmann S, Sasse C, Braus GH. Gene targeting in *Aspergillus fumigatus* by homologous recombination is facilitated in a nonhomologous end-joining-deficient genetic background. Eukaryot Cell. 2006;5:212–215.
