# Supplementary material for: Velvet domain protein VosA represses the zinc cluster transcription factor SclB regulatory network for Aspergillus nidulans asexual development, oxidative stress response and secondary metabolism
Source: PLoS Genet. 2018 Jul 25;14(7):e1007511. doi: 10.1371/journal.pgen.1007511 (PMC6078315; doi:10.1371/journal.pgen.1007511)
Supplement: S4 Table — (DOCX) [file pgen.1007511.s013.docx]

**S4 Table. *E. coli* strains used in this study.**

| DH5α | Invitrogen |
| --- | --- |
| DH10ß | Invitrogen |
| One Shot® TOP10 | Invitrogen |
| Rosetta II (DE3) | Novagen |
