# Supplementary material for: Velvet domain protein VosA represses the zinc cluster transcription factor SclB regulatory network for Aspergillus nidulans asexual development, oxidative stress response and secondary metabolism
Source: PLoS Genet. 2018 Jul 25;14(7):e1007511. doi: 10.1371/journal.pgen.1007511 (PMC6078315; doi:10.1371/journal.pgen.1007511)
Supplement: S5 Table — natRM = nourseothricin recyclable marker cassette, phleoRM = phleomycin recyclable marker cassette, ptrARM = pyrithiamine recyclable marker cassette, AN = A. nidulans, Afu = A. fumigatus. (DOCX) [file pgen.1007511.s014.docx]

**S5 Table. Plasmids used in this study.** natRM = nourseothricin recyclable marker cassette, phleoRM = phleomycin recyclable marker cassette, ptrARM = pyrithiamine recyclable marker cassette, AN = *A. nidulans*, Afu = *A. fumigatus*.

|  |  |  |
| --- | --- | --- |
| **Plasmid** | **Description** | **Reference** |
| pBluescript SK(+) | Cloning vector, *amp*^R^ | Thermo Scientific |
| pME3173 | ^P^*gpdA*::intron::*mrfp*::*h2A* cDNA in *EcoR*V and ^P^*gpdA*::*natR* in *Sma*I of pBluescript II KS | [3] |
| pME3741 | BiFC vector; ^P^*niiA*::n*eyfp*::*culA*::*niiA*^t^ and ^P^*niaD*::*candA*-*N*::C::*ceyfp*::*niaD*^t^::*AfpyrG*::*wA*, *bla* | [130] |
| pME4292 | Plasmid contains *sgfp* | This study |
| pME4304 | *six*-^P^*xylP*::β-*rec*::*trpC^t^*-*nat*^R^-*six* (natRM) | This study |
| pME4305 | *six*-^P^*xylP*::β-*rec*::*trpC^t^*-*phleo*^R^-*six* (phleoRM) | This study |
| pME4313 | Plasmid for BiFC containing niaD^t^-SwaI-^P^niaD/^P^niiA-PmeI-niiA^t^ | This study |
| pME4574 | ANΔ*veA*::natRM | This study |
| pME4575 | ANΔ*sclB*::natRM | This study |
| pME4576 | ^P^*sclB*::AN*sclB*::*sgfp*::natRM | This study |
| pME4577 | AN*sclB*::phleoRM | This study |
| pME4578 | natRM::^P^*niaD*::AN*sclB* | This study |
| pME4579 | ^P^*sclB*::*sgfp*::AN*sclB*::phleoRM | This study |
| pME4580 | ^P^*sclB*::AN*sclB*^S327A,T464A,S504-506A^::phleoRM | This study |
| pME4581 | ANΔ*fluG*::phleoRM | This study |
| pME4587 | ANΔ*abaA*::phleoRM | This study |
| pME4589 | ANΔ*brlA*::phleoRM | This study |
| pME4591 | ANΔ*flbB*::phleoRM | This study |
| pME4593 | ANΔ*flbC*::phleoRM | This study |
| pME4595 | ANΔ*flbD*::phleoRM | This study |
| pME4597 | ANΔ*flbE*::phleoRM | This study |
| pME4599 | ^P^*niaD*::AN*sclB*::*ceyfp* in *Swa*I restriction site, ^P^*niiA*::AN*rcoA*::*neyfp* in *Pme*I restiction site of pJG137 | This study |
| pME4600 | ^P^*niaD*::AN*sclB*::*ceyfp*, ^P^*niiA*::*neyfp* | This study |
| pME4601 | ^P^*niaD*::*ceyfp*, ^P^*niiA*::AN*rcoA*::*neyfp* | This study |
| pME4602 | ANΔ*velC*::phleoRM | This study |
| pME4603 | ANΔ*vosA*::natRM | This study |
| pME4605 | ANΔ*velB*::natRM | This study |
| pME4606 | AfuΔ*sclB*::ptrARM | This study |
| pME4609 | ^P^AN*sclB*::Afu*sclB* | This study |
| pME4610 | ^P^*sclB*::AN*sclB*^S327D,T464D,S504-506D^::phleoRM | This study |
| pSK485 | *six*-^P^*xylP*::β-*rec*::*trpCt*-*ptrA*-*six* (ptrARM) | [72] |
| pNI47 | *vosA* in pGEX-5X-1 (Amersham) | [2] |
