# Supplementary material for: Velvet domain protein VosA represses the zinc cluster transcription factor SclB regulatory network for Aspergillus nidulans asexual development, oxidative stress response and secondary metabolism
Source: PLoS Genet. 2018 Jul 25;14(7):e1007511. doi: 10.1371/journal.pgen.1007511 (PMC6078315; doi:10.1371/journal.pgen.1007511)
Supplement: S6 Table — Primers listed in this table are given with description of their purpose. Primers designed for usage with a seamless cloning kit (SCK). MssI sites, introduced by respective 5’ FW and 3’ rev primers, were chosen the way that no scar occurs after transformation into A. nidulans (i. e. primers were designed according to naturally occurring halves of the PmeI sites). FW = forward, rev = reverse, RT = qRT-Primer. (DOCX) [file pgen.1007511.s015.docx]

**S6 Table. Oligonucleotides used in this study.** Primers listed in this table are given with description of their purpose. Primers designed for usage with a seamless cloning kit (SCK). *Mss*I sites, introduced by respective 5’ FW and 3’ rev primers, were chosen the way that no scar occurs after transformation into *A. nidulans* (i. e. primers were designed according to naturally occurring halves of the *Pme*I sites). FW = forward, rev = reverse, RT = qRT-Primer.

|  |  |  |
| --- | --- | --- |
| **Name** | **Description** | **Sequence** |
| JG846 | natR FW (SCK) | TAA TCC TTC TTG TTT GGC GGC TCT GAG GTG CAG TG |
| JG847 | natR rev (SCK) | TAG ATA TCA GAG TTT TCA GGG GCA GGG CAT GCT CAT |
| JG848 | phleoR FW (SCK) | TAA TCC TTC TTG TTT CTT TGC CCG GTG TAT GAA ACC G |
| JG849 | phleoR rev (SCK) | TAG ATA TCA GAG TTT GGA TTA CCT CTA AAC AAG TGT ACC |
| JG863 | *veA* 5'FW incl. *Mss*I site (SCK) | ATC GAT AAG CTT GAT GTT TAA ACT TAA CCT AAA TGC ACG CAC T |
| JG865 | Δ*veA* 3' FW | ATA ATA TGG CCA TCT AAG AAT TCT GCC GGC GTT TAT TT |
| JG866 | *veA* 3' rev incl. *Mss*I site (SCK) | CTG CAG GAA TTC GAT GTT TAA ACG GAT TGA ATG CGG ACG C |
| JG985 | *veA* 5'rev | ACC TAT AGG CCT GAG CTT GAT GGG ATA ACA CAA AAT GCT |
| kt145 | Δ*velC* 5' rev (SCK) | CTA TAG GCC TGA GTG TAA GAA GTC AAG AAG CGG TCA ATG |
| kt146 | *velC* 3' FW (SCK) | ATA ATA TGG CCA TCT ATG TTT TGA GGG ACT CCA ACT C |
| kt203 | *velC* 5' FW incl. *Mss*I site (SCK) | CTG CAG GAA TTC GAT GTT TAA ACG CTG AAG TTT GTG GGA G |
| kt204 | *velC* 3' rev incl *Mss*I (SCK) | ATC GAT AAG CTT GAT GTT TAA ACG CTG TAA GTT GGA TCA GC |
| kt208b | *sclB* (*AN0585*) 5' FW incl. *Mss*I site (SCK) | CTG CAG GAA TTC GAT GTT TAA ACC TGG TAT GAA CGA CTT TCC |
| kt209 | *sclB* (*AN0585*) 5' FW incl. *Mss*I site (SCK) | CTG CAG GAA TTC GAT GTT TAA ACC ATC GCT CTG GTA GCT TC |
| kt211 | *sclB* (*AN0585*) 3' FW (SCK) | ATA ATA TGG CCA TCT CTC AAC CGC CTA TCA CTC TAG |
| kt214 | Δ*sclB* (*AN0585*) 5' rev (SCK) | CTA TAG GCC TGA GTG CTG GTA GTC TTA CGG TGA GTT G |
| kt215 | *sclB* (*Afu6g11110*) 5' FW incl. *Mss*I site (SCK) | CTG CAG GAA TTC GAT GTT TAA ACA GGA TTC GGT GAT TTC TTT C |
| kt218 | *sclB* (*Afu6g11110*) 3' FW (SCK) | ATA ATA TGG CCA TCT TCG TCT CCT ACA GCA GGA C |
| kt219 | *sclB* (*Afu6g11110*) 3' rev incl. *Mss*I site (SCK) | CTG CAG GAA TTC GAT GTT TAA ACC AAA GAC CCA GCT AAA AAC |
| kt221 | Δ*sclB* (*Afu6g11110*) 5' rev (SCK) | CTA TAG GCC TGA GTG TTG GTA GGT TGA GGG TCC C |
| kt224 | *sclB* (*AN0585*) 3' rev incl. *Mss*I site (SCK) | ATC GAT AAG CTT GAT GTT TAA ACC CTA CTT TCA CAA CGA GG |
| kt225 | *sclB* (*AN0585*) 3' rev incl. *Mss*I site (SCK) | ATC GAT AAG CTT GAT GTT TAA ACC GGA GCG TAT CAC CTA TC |
| kt228 | *sclB* (*AN0585*) 5' rev for C’*-sgfp* fusion (SCK) | ACC ACC GCT ACC ACC GTC GTT GAC CAT ATC ATC CAA C |
| kt230 | *sclB* (*AN0585*) gene FW for N'-*sgfp* fusion (SCK) | GGT GGT AGC GGT GGT CAA TCA CTC GTC CTC CCT C |
| kt251 | ^P^*niiA* FW (SCK) | ATA ATA TGG CCA TCT GAT GGC GGG CGC GGT GAT T |
| kt300 | ^P^*niiA* rev (SCK) | GAT GGC GGG CGC GGT GAT |
| kt302 | ^P^*niiA* FW (SCK) | GTG AGA GTA TGG GAT AGG AAA ATA |
| kt307 | *sclB* (*AN0585)* 5' rev for N'-*sgfp* fusion (SCK) | GCC CTT GCT CAC CAT CTG GTA GTC TTA CGG TGA GTT G |
| kt337 | ^P^*niiA* FW (SCK) | ATA ATA TGG CCA TCT GTG AGA GTA TGG GAT AGG AAA ATA |
| kt340 | *fluG* 5' FW incl*. Mss*I site (SCK) | CTG CAG GAA TTC GAT GTT TAA ACT CGC CCG TAC GAT TCA GAA ATG |
| kt342 | Δ*fluG* 5' rev (SCK) | CTA TAG GCC TGA GTG GGC GAT GAA CCA GCA AAC TAA AGG AC |
| kt343 | *fluG* 3' FW (SCK) | ATA ATA TGG CCA TCT GTC TAA TCT TTC TCC TGA GCG TAT TCA C |
| kt345 | *fluG* gene FW for OE (SCK) | ACC GCG CCC GCC ATC ATG GCC ACT CTC TCT TCA CTC CG |
| kt347 | *aflR* 5' FW incl. *Mss*I site (SCK) | CTG CAG GAA TTC GAT GTT TAA ACA GCC CCC TGA GTC CCT GAT G |
| kt349 | Δ*aflR* 5' rev (SCK) | CTA TAG GCC TGA GTG GAT ATT TGC ATA TGA TAC AGG CCC GCA TTG |
| kt350 | *aflR* 3' FW (SCK) | ATA ATA TGG CCA TCT GGT TGA ATA ATC TGG AAT GAT ATT TAT GCG ATC |
| kt353 | *aflR* gene FW for OE (SCK) | ACC GCG CCC GCC ATC ATG GAG CCC CCA GCG ATC AG |
| kt354 | *abaA* 5' FW incl. *Mss*I site (SCK) | CTG CAG GAA TTC GAT GTT TAA ACC CTG GTC AGA CAC TGA GCA TG |
| kt355 | Δ*abaA* 5' rev (SCK) | CTA TAG GCC TGA GTG GGA GCA GAC CCC AAG ATT CGC TC |
| kt356 | *abaA* 3' FW (SCK) | ATA ATA TGG CCA TCT CCT CCT TTA CCA TGT CTA TGA ACA GAC G |
| kt361 | *aflR* 3' rev incl. *Mss*I site (SCK) | ATC GAT AAG CTT GAT GTT TAA ACA AAA TAT GAT CGT GCT TCG GCA CTT GG |
| kt364 | *fluG* 3' rev incl. *Mss*I site (SCK) | ATC GAT AAG CTT GAT GTT TAA ACA CGC CGC CGC TAA GCG |
| kt379 | EMSA *sclB* 5' with *vosA* binding site FW | GAC TTT CCT CCG CGG ACG CCG CGT CGA TTT TAG |
| kt380 | EMSA *sclB* 5' *vosA* binding site rev | CTA AAA TCG ACG CGG CGT CCG CGG AGG AAA GTC |
| kt407 | *sclB* (*AN0585*) FW for BI-FC | CAT ACT CTC ACA TTT ATG CAA TCA CTC GTC CTC CC |
| kt409 | *rcoA* FW for BI-FC | CGC CCG CCA TCG TTT ATG CGC AGC ATT GAC CAA CC |
| kt416 | *eyfp*-C FW for BI-FC | TGC GAA CCC GTA TTT TCA CTT GTA CAG CTC GTC CAT |
| kt417 | *eyfp*-C rev for BI-FC | CGC CCG GCC TGC AAG ATC |
| kt418 | *rcoA* rev for *eyfp*-N Bi-FC | CGT GGC GAT GGA GCG CCG TCC AGT GTA CGC GGA G |
| kt420 | *eyfp*-N FW for BI-FC | CGC TCC ATC GCC ACG GTG |
| kt422 | *eyfp*-N rev for BI-FC | TAT CCT CGT CAG TTT TCA CAT GAT ATA GAC GTT GTG GCT |
| kt423 | *sclB* (*AN0585*) rev for BI-FC for *eyfp*-N | CGT GGC GAT GGA GCG GTC GTT GAC CAT ATC ATC C |
| kt430 | Rev *sclB* dephosphorylation mutant site 1 | GTG CGA CTG AAG TCA TTG AC |
| kt431 | FW *sclB* dephosphorylation mutant from site 1 on | TGA CTT CAG TCG CAC CAA CGG GAC ATG TCC CAA TC |
| kt432 | Rev *sclB* dephosphorylation mutant site 2 | CTC TGG AGC TTG CTG CTG C |
| kt433 | FW *sclB* dephosphorylation mutant from site 2 on | CAG CAA GCT CCA GAG CCC CGC CAG GGC TTC ATG |
| kt434 | Rev *sclB* dephosphorylation mutant site 3 | GGC GGC CAA GCC TAG TTG GCT TTG GCT TTG GCG C |
| kt442 | FW ztfA dephosphorylation mutant from site 3 on | CCA ACT AGG CTT GGC CGC CGC G |
| kt487 | *brlA* 5' FW incl. *Mss*I site (SCK) | CTG CAG GAA TTC GAT GTT TAA ACG CCC AAC CCC ACA CTG |
| kt488 | Δ*brlA* 5' rev (SCK) | CTA TAG GCC TGA GTG GTC TTC GAG CGA CGG GGC |
| kt489 | *brlA* 3' FW (SCK) | ATA ATA TGG CCA TCT AAC AGA AAC AAA GAG GGC TGA TC |
| kt490 | *brlA* 3' rev incl. *Mss*I site (SCK) | ATA AGC TTG ATG TTT AAA CAA GAA CGT AAC CTA CCG TAA G |
| kt515 | *flbB* 5' FW incl. *Mss*I site (SCK) | CTG CAG GAA TTC GAT GTT TAA ACG CTC CTT CTT CCA CTT C |
| kt516 | Δ*flbB* 5' rev (SCK) | CTA TAG GCC TGA GTG GGT GGT CGA GCT GTG AAT AG |
| kt517 | *flbB* 3' FW (SCK) | ATA ATA TGG CCA TCT CCT GAC AGC TCG CTT TTT TTC |
| kt518 | *flbB* 3' rev incl*. Mss*I site (SCK) | ATC GAT AAG CTT GAT GTT TAA ACA TAG TGT ATG ACA CGC CC |
| kt519 | *flbC* 5' FW incl. *Mss*I site (SCK) | CTG CAG GAA TTC GAT GTT TAA ACC CAC TGC TCA AGC TCA G |
| kt520 | Δ*flbC* 5' rev (SCK) | CTA TAG GCC TGA GTG TGA GGA TAG TCG TTT TGA AAG AG |
| kt521 | *flbC* 3' FW (SCK) | ATA ATA TGG CCA TCT TCG TTT CAA TCG ACC TGC CC |
| kt522 | *flbC* 3' rev incl. *Mss*I site (SCK) | ATC GAT AAG CTT GAT GTT TAA ACG GCG TCG AGA AGG C |
| kt523 | *flbD* 5' FW incl. *Mss*I site (SCK) | CTG CAG GAA TTC GAT GTT TAA ACG AAC AAG TGC CGA CTC |
| kt524 | Δ*flbD* 5' rev (SCK) | CTA TAG GCC TGA GTG TTG CGA AAC TGT GTT GGT GAT G |
| kt525 | *flbD* 3' FW (SCK) | ATA ATA TGG CCA TCT ACG ATC ACA CGA CTC TCT TC |
| kt526 | *flbD* 3' rev incl*. Mss*I site (SCK) | ATC GAT AAG CTT GAT GTT TAA ACA CCG TAG ACT TGT CCA G |
| kt527 | *flbE* 5' FW incl. *Mss*I site (SCK) | CTG CAG GAA TTC GAT GTT TAA ACG ACT TGT TTG CTC GTC TC |
| kt528 | Δ*flbE* 5' rev (SCK) | CTA TAG GCC TGA GTG GGT AAG GCG ACG ACG GC |
| kt529 | *flbE* 3' FW (SCK) | ATA ATA TGG CCA TCT TTG CTG TAC GAG TTA TAT TAC GAC |
| kt530 | *flbE* 3' rev incl*. Mss*I site (SCK) | ATC GAT AAG CTT GAT GTT TAA ACT AGT GAG ACC TAC CAG C |
| kt603 | AN*sclB* 5’ rev with overhang for Afu*sclB* | GGA AGG ACG AGT GAT TGC ATC TGG TAG TCT TAC GGT GAG |
| kt651 | Rev *sclB* phosphorylation mutant site 1 | GGT CGA CTG AAG TCA TTG AC |
| kt652 | FW *sclB* phosphorylation mutant from site 1 on | TGA CTT CAG TCG ACC CAA CGG GAC ATG TCC CAA TC |
| kt653 | Rev *sclB* phosphorylation mutant site 2 | CTC TGG ATC TTG CTG CTG C |
| kt654 | FW *sclB* phosphorylation mutant from site 2 on | CAG CAA GAT CCA GAG CCC CGC CAG GGC TTC ATG |
| kt655 | Rev *sclB* phosphorylation mutant site 3 | GTC GTC CAA GCC TAG TTG GCT TTG GCT TTG GCG C |
| kt657 | FW *sclB* phosphorylation mutant from site 3 on Sec Vers | CTA GGC TTG GAC GAC GA |
| kt696 | *sclB* ORF rev | ATA TGG CCA TCT CAC TTA GTC GTT GAC CAT ATC ATC C |
| SR05 | *velB* 5' FW incl*. Mss*I site (SCK) | CTG CAG GAA TTC GAT GTT TAA ACC GTG CAG TCA GTC TAC CTA C |
| SR06 | Δ*velB* 5' rev (SCK) | CTA TAG GCC TGA GTG AAC CGC GAT CGT TCG CGG |
| SR07 | *velB* 3' FW (SCK) | ATA ATA TGG CCA TCT AGA CCG TAT ATT GTT TCA TAA ATC C |
| SR08 | *velB* 3' rev incl. *Mss*I site (SCK) | ATC GAT AAG CTT GAT GTT TAA ACC CGC TGT ACA TGT AAT GTC CG |
| SR11 | *vosA* 5' FW incl. *Mss*I site (SCK) | CTG CAG GAA TTC GAT GTT TAA ACA TCT GGC TAG GCA AGG TAG GCT TTC AGG |
| SR12 | Δ*vosA* 5' rev (SCK) | CCT ATA GGC CTG AGT CAA GGC GAG CAC TAT GAG AG |
| SR13 | *vosA* 3' FW (SCK) | ATA ATA TGG CCA TCT GGA TTC TCG TTT GTG GAA CAC |
| SR14 | *vosA* 3' rev incl. *Mss*I site (SCK) | ATC GAT AAG CTT GAT GTT TAA ACC ATG AGC CAT GCT TCT GTA AC |
| SR193 | *nyfp*-N FW with ATG for Bi-FC control (SCK) | CGC CCG CCA TCG TTT ATG GTG AGC AAG GGC GAG |
| SR195 | *eyfp*-C FW with ATG for Bi-FC control (SCK) | ACT CTC ACA TTT ATG GCC GAC AAG CAG AAG AAC G |
| JG787 | *flbA* A RT | CCC TTC TTC TTC TTC CCC TCC T |
| JG788 | *flbA* B RT | AAA ACT GGG TGT GGT TGT GGT G |
| JG793 | *aflR* A RT | GAA GGC AGG ACC ACC AGT TAC A |
| JG794 | *aflR* B RT | CCC TCA AGA AGC GAA GGA GAA A |
| JG814 | *veA* A RT | CAA CGA GCA TCA GCA CAA ACA T |
| JG815 | *veA* B RT | AGC AGG AAT CGG CGT AGA AGA T |
| JG816 | *velB* A RT | CCC CTC CGT GTA TCC GTC TAA T |
| JG817 | *velB* B RT | AGC CGA GTG CTT CAC AAG ATT T |
| JG818 | *vosA* A RT | CTT CCA TTC CAC CGT CTA CTG C |
| JG819 | *vosA* B RT | CGT CCG TCT TTC GCA TTT CA |
| JG824 | *stcU* A RT | TTG AGC ACT TCG GAT ACC TGG A |
| JG825 | *stcU* B RT | TTG GAA CTT GTG AGG ATG ATG C |
| JG1482 | *easA* A RT | ATC ACC AGC GAA CCT CTC TTA G |
| JG1483 | *easA* B RT | AGG CTT TCA ATC ACC AGA CTC C |
| JG1484 | *easB* A RT | TTC GTC AAG TTT AGT GGC GTT A |
| JG1485 | *easB* B RT | CGT TGT GGG TCA AGA AGT AGG T |
| JG1486 | *easC* A RT | ACC TTC ATT GGA AAC ATC AAC G |
| JG1487 | *easC* B RT | TAG GGT CGT CAG GGA TTC TG |
| JG1488 | *easD* A RT | AGC GAC TTC CAC CAT TAC AGT G |
| JG1489 | *easD* B RT | AGT TTC TGC TTC CCT GAT GTT C |
| kt272 | *sclB* (*AN0585*) A RT | TTC AGT CTC ACC AAC GGG ACA T |
| kt273 | *sclB* (*AN0585*) B RT | GAT ACG CGA GTT TGG GTT TTC C |
| kt274 | *brlA* A RT | CAG GAT CAC TCC CCA ACA ACA C |
| kt275 | *brlA* B RT | GTA AGC GAG TCC TTG AGC GAC A |
| kt278 | *15s rNRA* A RT | GAT CCG CGA AAA ACC TTA CCA C |
| kt279 | *15s rNRA* B RT | TGG CAC GTC TAT AGC CCA CAG T |
| kt308 | *gpdA* A RT | AAC GCT TCT TGC ACC ACC AA |
| kt309 | *gpdA* B RT | ACC AGT GGA GGA GGG GAT GA |
| kt310 | *velC* A RT | CCA ATC GAC TCC GCT CCT CT |
| kt311 | *velC* B RT | AGA AGC ATG CCG GTG GTT TT |
| kt312 | *h2A.X* A RT | TCT CGA GCT TGC TGG AAA CG |
| kt313 | *h2A.X* B RT | CAC CCT GGG CAA TAG TGA CG |
| kt316 | *h2A.X* ^af^ A RT | TGG AGT ATC TCG CTG CTG AA |
| kt317 | *h2A.X* ^af^ B RT | GGA GAT GGC GAG GAA TGA TA |
| kt320 | *sclB* (*Afu6g11110*) ^af^ A RT | CAG CAG CAG ACT AGG GGT TC |
| kt321 | *sclB* (*Afu6g11110*) ^af^ B RT | TGA TGC GGA GCT ACT TCT CC |
| kt332 | *brlA* ^af^ A RT | TCA TCA AGC AGG TGC AGT TC |
| kt333 | *brlA* ^af^ B RT | TTG GAG TGG CTC TTC ATG TG |
| kt397a | *flbB* A RT | AGT TCG ACT TCT CGT CAG TTC C |
| kt398a | *flbB* B RT | TGG GGA TTG TCT TCA AAT ATC C |
| kt399a | *flbC* A RT | ATC TCA TCT GCA GGC TCT TAC C |
| kt400a | *flbC* B RT | GTT GTT GAG CTG TAA TCG GTG A |
| kt401a | *flbD* A RT | CAA CAA AGC ATC AAC AGC TCT C |
| kt402a | *flbD* B RT | GGT CCA TGA GGT ATA GGG TCT G |
| kt404 | *fluG* A RT | GAC ATC AAT CTG CTG AAA TCC A |
| kt405 | *fluG* B RT | TCG CGT GTA TAT GGG TAA GAT G |
| kt436 | *ausA* A RT | AGG TGG AGA ACT GCT CAG GA |
| kt437 | *ausA* B RT | CGA AGG AAA CGG ACT GAG AG |
| kt438 | *ausF* A RT | TGT CCA CCA CAC GAG AAA AG |
| kt439 | *ausF* B RT | TGC GAA TGG AGA GAA TTT CC |
| kt440 | *ausH* A RT | GGA CTT CCA AGG GCT AAA GG |
| kt441 | *ausH* B RT | ACT CGG TCT CAA ATC GAC CA |
| kt491 | *nsdD* A RT | TCA TCT CAC CAG CCA CAA TTA C |
| kt492 | *nsdD* B RT | CAG AGG TCA TAA CAG TGC TTG C |
| kt531 | *catB* A RT | TTA ATC GAA TCT CGA ACG ACC T |
| kt532 | *catB* B RT | GGT CGT GTT GTC GTG GTA GTA A |
| kt533 | *trxR* A RT | CCC TAG AGG CTA ACG GTC TTT T |
| kt534 | *trxR* B RT | ATG TAT CCG TCC TCA TCG AGT T |
| kt548 | *flbE* A RT | TGA CGA AGA TGA GGA TGG TAT G |
| kt549 | *flbE* B RT | TGT TAC TAG ACG ACC CAT CAC G |
| kt550 | *sfgA* A RT | ACT TTT AGC GCT CTT CGA GAT G |
| kt551 | *sfgA* B RT | AGG GTG ATT CAT TTC AGC AAC T |
| kt578 | *catA* A RT | AGG AAG TTC TGG GCA ATG TG |
| kt579 | *catA* B RT | GTC CTT GAG CAC CTT GAA GC |
| kt584 | *napA* (*AN7513*) A RT | CCG GCA TCT TAC GAC ATT CT |
| kt585 | *napA* (*AN7513*) B RT | ACT TTG TGG CAG GGT TGT TC |
| kt586 | *rsmA* A RT | ATC GCT GGC AGT CAT TAT CC |
| kt587 | *rsmA* B RT | TAA TTC CGA TTC CGT CCT TG |
| kt588 | *glrA* A RT | CCG AAG TTG AGG ATT TGC AT |
| kt589 | *glrA* B RT | TCG ACG TTG GTG TTT TGG TA |
| kt590 | *trxA* A RT | GAA ATT CGC CCA GAC CTA CA |
| kt591 | *trxA* B RT | CCA ACC ACA TCG CTA ACC TT |
| kt721 | DNA probe for EMSA sclB | GAA AAA TAA GAA GGG ACA AAA AAG GGA AAA AAG GAA AAA A |
| kt781 | DNA probe for EMSA sclB | CTT TTT ATT CTT CCC TGT TTT TTC CCT TTT TTC CTT TTT T |
| kt790 | DNA probe for EMSA sclBΔ1 | GAA AAA TAA GAA GGG ACA AAA AAG GAA AAA A |
| kt791 | DNA probe for EMSA sclBΔ1 | CTT TTT ATT CTT CCC TGT TTT TTC CTT TTT T |
| kt792 | DNA probe for EMSA sclBΔ2 | GAA AAC AAA AAA GGG AAA AAA GGA AAA AA |
| kt793 | DNA probe for EMSA sclBΔ2 | CTT TTG TTT TTT CCC TTT TTT CCT TTT TT |
| kt794 | DNA probe for EMSA sclBΔ1Δ2 | AAA GGG AAG AAA ACA AAA AAG GAA AAA AGG AA |
| kt795 | DNA probe for EMSA sclBΔ1Δ2 | TTT CCC TTC TTT TGT TTT TTC CTT TTT TCC TT |
