# Supplementary figures and images for: Correction: Velvet domain protein VosA represses the zinc cluster transcription factor SclB regulatory network for Aspergillus nidulans asexual development, oxidative stress response and secondary metabolism
Source: PLoS Genet. 2018 Aug 29;14(8):e1007638. doi: 10.1371/journal.pgen.1007638 (PMC6114284; doi:10.1371/journal.pgen.1007638)

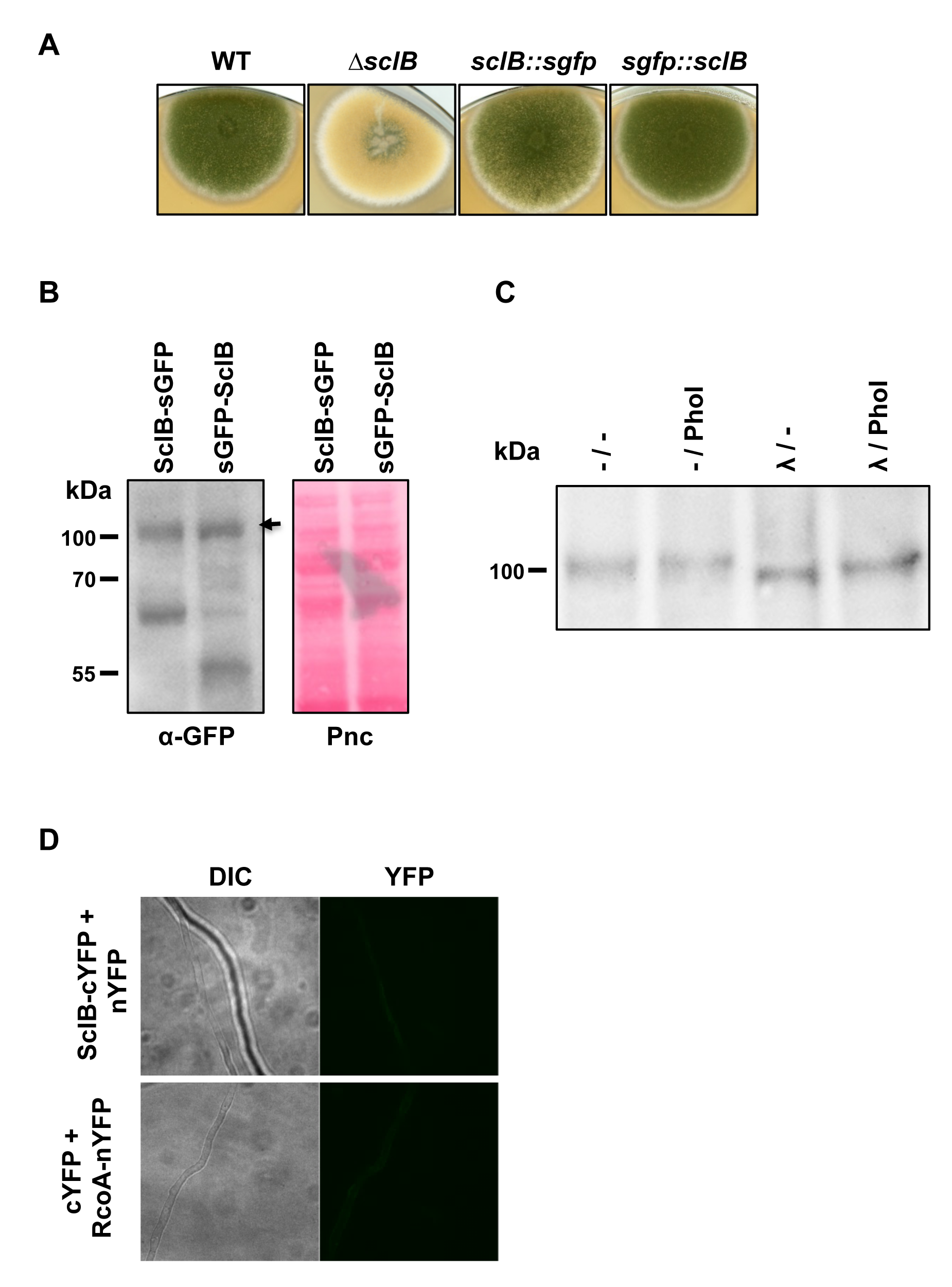

Supplement: S8 Fig — A) Strains expressing SclB either N- or C-terminally tagged with sGFP in ΔsclB background, ΔsclB and wildtype (WT) were point inoculated on solid MM and grown for 4 days in light. B) SclB-GFP and GFP-SclB fusion proteins expressed under native promoter are visualized in a western hybridization assay employing an α-GFP antibody (GFP) and Ponceau staining as loading control (Pnc). The black arrow indicates bands corresponding to full-length fusion proteins (in silico prediction 87.46 kDa). C) Protein crude extracts of GFP-SclB grown vegetatively were mixed with phosphatase inhibitor cocktail (-/PhoI), with Lambda phosphatase (λ/-), or Lambda phosphatase and phosphatase inhibitor cocktail (λ/PhoI). A control sample was left untreated (-/-). A subsequent western hybridization assay employing α-GFP antibody visualizes protein bands. D) Two strains, either expressing sclB::cyfp and the free second half of the split YFP (nyfp; upper part), or free cyfp and rcoA::nyfp (lower part), under control of a bi-directional nitrate promoter were constructed. Strains were inoculated in liquid MM and analyzed with fluorescence microscopy after 36 h at 30°C. (TIF) [file pgen.1007638.s001.tif]
